# Temporal drift of sleep-wake representations in hypothalamic neuronal ensembles

**DOI:** 10.1038/s41467-026-75792-1

**Published:** 2026-07-28

**Authors:** Yudong Yan, Niccolò Calcini, Thomas Rusterholz, Carolina Gutierrez, Antoine Adamantidis

**Affiliations:** 1 Zentrum für Experimentelle Neurologie, Department of Neurology, Inselspital University Hospital Bern, University of Bern, Bern, Switzerland; 2 Department of Biomedical Research, University of Bern, Bern, Switzerland; 3 Present Address: Department of Neuroscience, Yale School of Medicine, Yale University, New Haven, CT USA

**Keywords:** Hypothalamus, Sleep

## Abstract

Correlative and causal evidence implicates distinct genetically-defined and evolutionary-conserved hypothalamic neurons in regulating wakefulness, non-rapid eye movement (NREM), and rapid eye movement (REM) sleep. The prevailing view is that these circuits govern sleep-wake states by recruiting stable, invariant neuronal substrates, yet, this remains unknown. Here, we show that inhibitory, excitatory, hypocretins/orexins-, and melanin concentrating hormone-expressing neurons in hypothalamus did not exhibit stable state-specific activities using longitudinal single cell calcium imaging in freely-moving, sleeping male mice. Instead, their activity patterns shift across sleep-wake states over time, while the distribution of active neurons in each sleep state remains stable. While sleep deprivation minimally affected the selectivity of these activity patterns, we found that the sleep-promoting drug diazepam recruited NREM sleep-active cells that were previously inactive or wake-active. These findings indicate that while individual neurons exhibit dynamic, state-dependent shifts of their activity, the overall organization of sleep-wake neural populations remains stable.

## Abstract (web summary)

Hypothalamic neurons encode sleep–wake states, but their long-term stability is unclear. Here, authors show that although single-cell state selectivity drifts over days, the overall ensemble representations remain stable and are altered by diazepam.

## Introduction

The hypothalamus is a core brain structure involved in maintaining physiological functions including food intake^1,2^, reproduction^3,4^, fight-or-flight responses^5,6^ and sleep-wake states^7–9^. The lateral hypothalamic area (LH) encompasses diverse neuron populations characterized by distinct biological and chemical markers, including vesicular glutamate transporters 2 (Vglut2, a marker for glutamatergic neurons), γ-Aminobutyric acid (GABA, Vgat), as well as neuropeptides hypocretins/orexins (Hcrt/OX) and melanin-concentrating hormone (MCH)^10^. Inhibitory LH-Vgat neurons and excitatory LH-Vglut2 neurons show high discharge rate during both wakefulness and REM sleep as compared to NREM sleep, and their activation promotes wakefulness^11–14^. Similarly, the activity of LH-Hcrt/OX neurons, which co-release glutamate^13,15,16^, correlate with sensory inputs, emotional responses, locomotion, and sleep-to-wake transitions^17–19^, while their optogenetic activation causally induced awakening from both NREM and REM sleep^20^. In contrast, the firing rate of LH-MCH neurons is highest during REM sleep and relatively low during both wakefulness and NREM sleep^21^. Inhibitory neurons in the preoptic area (POA) of hypothalamus comprise sleep-active populations that preferentially discharge during NREM and/or REM sleep^22–24^ and have been implicated in the regulation of sleep homeostasis^25,26^.

The prevailing model posits that hypothalamic neurons that govern sleep-wake states are clustered into wake-, NREMs- or REMs-active groups, consistent with findings from classical c-fos staining^23,27^, electrophysiology^21,28^, or population-based calcium activity recordings^29,30^, suggesting that neurons within these functional clusters are highly stable across repeated sleep-wake transitions.

To test this hypothesis, we conducted longitudinal in vivo single-cell calcium (Ca²⁺) imaging of genetically targeted wake- and sleep-promoting neuronal subpopulations in the LH and POA (LH-Vgat, LH-Vglut2, LH-Hcrt/OX, LH-MCH, and POA-Vgat) across successive sleep-wake state transitions in freely sleeping mice. By categorizing and tracking neuron activity across sleep states over multiple days, as well as across sleep deprivation (SD), we show that while the overall number of state-dependent functional populations remains stable, a significant proportion of individual neurons shift their activity between categories at different times of day. Sleep deprivation does not evidently disturb neuronal population stability. However, administration of the sleep medication, diazepam (DZP), strongly alters single-cell state selectivity and activity, as well as the stability of neuronal populations encoding NREM sleep in the POA.

## Results

### LH subpopulations show different sleep state-dependent activity

**Fig. 1:**
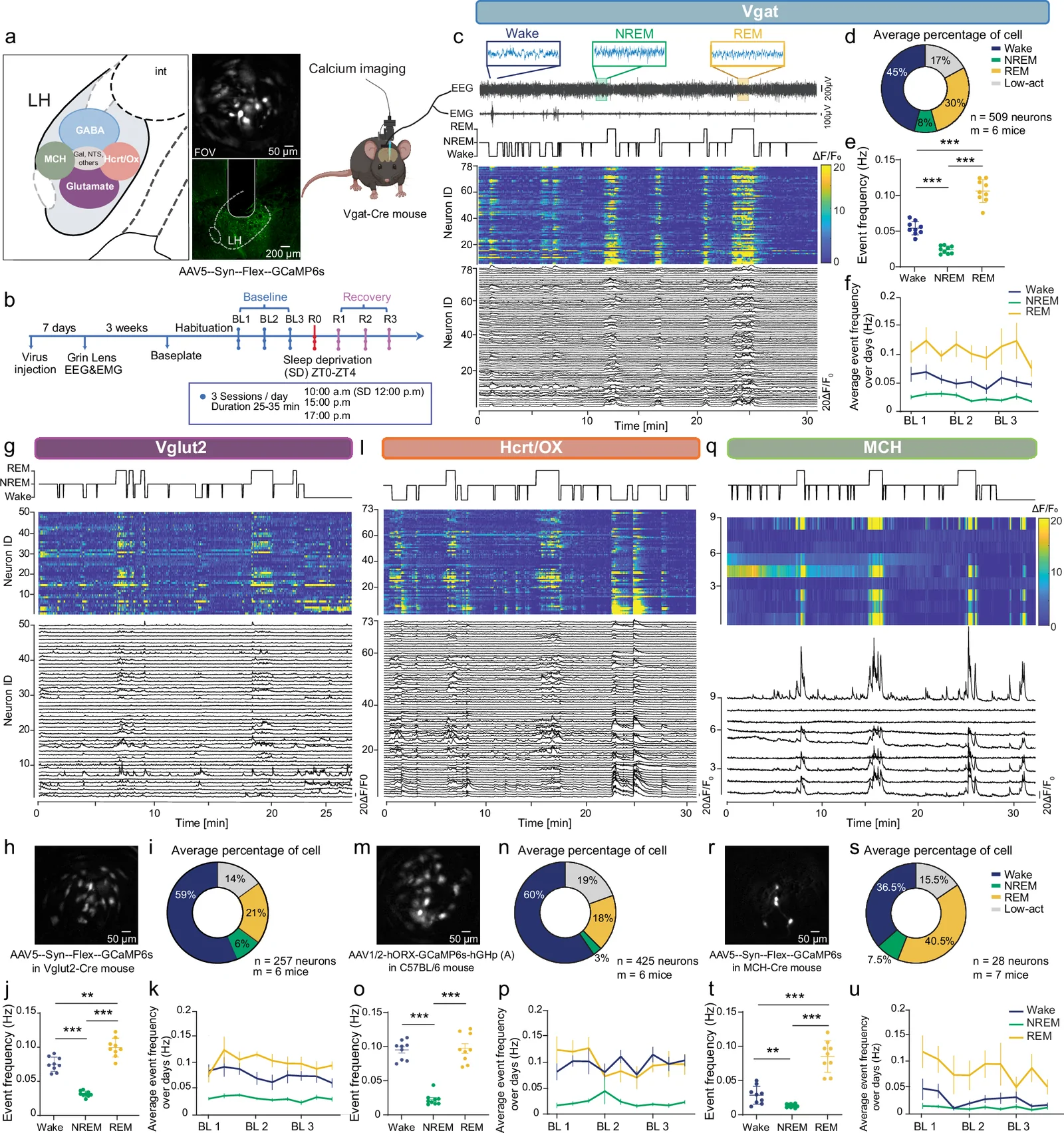
LH neuronal subpopulations exhibit state-dependent activity across sleep-wake cycles during baseline (BL) recordings. **a** (Left) Anatomical illustration depicting four major neuronal populations within the LH: glutamatergic, GABAergic, hypocretin/orexin (Hcrt/OX), and melanin-concentrating hormone (MCH) neurons. GABAergic and glutamatergic neurons can co-express Hcrt/OX or MCH. (Middle) Representative field of view of LH-Vgat neurons acquired in vivo using miniscope (scale bar: 50 µm), along with GCaMP6s expressing in situ (Scale bar: 200 µm). (Right) Schematic of the experimental setup combining calcium imaging and EEG/EMG recordings in a Vgat-Cre mouse (Adapted from BioRender, Yan, Y. (2026) https://BioRender.com/3r5iisx). **b** Timeline of surgical procedures and longitudinal imaging protocol. **c** Representative EEG (with magnified wake, NREM, and REM traces), EMG, and Ca^2+^ transients from LH-Vgat neurons across sleep-wake states. From top to bottom: EEG, EMG, hypnogram, color-coded and raw Δ*F*/*F*_0_ traces from 78 single neurons. **d** Average proportions of LH-Vgat neurons that are low-activity or show maximal Ca²⁺ activity during wake, NREM, or REM sleep across baseline recordings (*n *= 509 neurons from *m* = 6 mice). **e** Session mean ± SEM of Ca²⁺ event frequency of LH-Vgat neurons during wake, NREM, and REM sleep in baseline recordings (*n* = 9 sessions). **f** Mean ± SEM Ca²⁺ event frequency of LH-Vgat neurons in each baseline recording (*n* = 509 neurons). **g**, **l**, **q** Representative hypnogram, color-coded, and raw Δ*F*/*F*_0_ traces from LH-Vglut2, LH-Hcrt/OX, and LH-MCH neurons. **h**, **m**, **r** Representative in vivo miniscope fields of view of LH-Vglut2, LH-Hcrt/OX, and LH-MCH neurons, scale bar: 50 µm. **i**, **n**, **s** Average proportions of LH-Vglut2 neurons (*n* = 257 neurons from *m* = 6 mice), LH-Hcrt/OX neurons (*n* = 425 neurons from *m* = 6 mice), and LH-MCH neurons (*n* = 28 neurons from *m* = 7 mice) that are low-activity or show maximal activity during wake, NREM, or REM sleep. **j**, **o**, **t** Session mean ± SEM Ca²⁺ event frequency (Hz) of LH-Vglut2, LH-Hcrt/OX, and LH-MCH neurons during wake, NREM, and REM sleep (*n* = 9 sessions). **k**, **p**, **u** Mean ± SEM Ca²⁺ event frequency (Hz) of LH-Vglut2 (*n* = 257 neurons), LH-Hcrt/OX (*n* = 425 neurons), and LH-MCH (*n *= 28 neurons) neurons during wake, NREM, and REM sleep in each baseline recording. **P < 0.01; ***P < 0.001. Statistical analyses and source data are listed in the Source Data file.

We first characterized the single-cell activity of GABAergic (LH-Vgat), glutamatergic (LH-Vglut2), hypocretins/orexins (LH-Hcrt/OX), and melanin-concentrating hormone (LH-MCH) neurons in the LH across sleep-wake states using longitudinal one-photon Ca²⁺ imaging combined with simultaneous electroencephalogram (EEG) and electromyogram (EMG) recordings in freely-behaving mice (Fig. 1a and b; Supplementary Fig. 1).

To characterize the dynamics of single cell LH-Vgat neurons, we stereotactically injected a Cre-dependent AAV-Syn-flex-GCaMP6 virus into the LH of Vgat-Cre mice, resulting in specific expression in LH-Vgat neurons (Fig. 1a; Supplementary Fig. 1a). During 3-day baseline sleep recordings, LH-Vgat neurons exhibited a significant increase in the frequency of Ca²⁺ events during REM sleep as compared to NREM sleep and wakefulness (Fig. 1c, e). We classified LH-Vgat neurons into wake-max, NREM-max, and REM-max clusters based on their peak activity during each vigilance state. The calcium transients of each cluster preceding sleep-wake state transitions confirmed the validity of the clustering method (Supplementary Fig. 2). LH-Vgat neurons were preferentially activated during either REM sleep (30%) or wakefulness (45%), while a minor subpopulation of cells remained active during NREM sleep (8%) or showed no activity modulation across states (17%; Fig. 1d). Ca²⁺ activity in each vigilance state was consistent across each of the baseline recording sessions (Fig. 1f).

We next examined the activity of LH-Vglut2 neurons by expressing Cre-dependent AAV-Syn-flex-GCaMP6 virus in the LH of Vglut2-Cre mice (Fig. 1g, h; Supplementary Fig. 1a). We found that the majority of neurons were preferentially active during wakefulness (59%) or REM (21%) sleep, while a small subset of cells was active during NREM sleep (6%) or low-activity (14%) during baseline sleep (Fig. 1i). Similar to LH-Vgat neurons, LH-Vglut2 neurons showed the highest frequency of Ca²^+^ events during REM sleep as compared to NREM sleep and wakefulness. Their activity during wakefulness was greater than during NREM sleep, though lower than during REM sleep (Fig. 1g, j). This pattern of neuronal activity was consistent across all baseline recording sessions (Fig. 1k).

The expression of GCaMP6s in LH-Hcrt/OX neurons was achieved by stereotactic injection of AAV1/2-hORX-GCaMP6s-hGHp(A) into the LH of C57BL/6 mice (Fig. 1l, m; and Supplementary Fig. 1b). The majority of LH-Hcrt/OX neurons were preferentially active during wakefulness (60%) while few remained active during REM sleep (18%; Supplementary Fig. 2c, f, h) or NREM sleep (3%) (Fig. 1n). The frequency of Ca²⁺ events in LH-Hcrt/OX neurons was similar during wakefulness and REM sleep, and both were significantly higher than during NREM sleep (Fig. 1l, o). This activity pattern was consistent across all baseline sessions (Fig. 1p).

To image the activity of LH-MCH neurons, we stereotactically injected AAV-Syn-flex-GCaMP6 in the LH of MCH-Cre mice (Fig. 1q, r; Supplementary Fig. 1b). Our results showed that a large subset of MCH neurons were predominantly active during REM sleep (40.5%), while others were maximally active during wakefulness (36.5%) and to a lesser extent NREM sleep (7.5%; Fig. 1s). LH-MCH neurons displayed significantly elevated Ca²⁺ event frequencies at the transition to (Supplementary Fig. 2d, f), and during REM sleep, while event frequencies remained very low during both wakefulness and NREM sleep across all baseline recording sessions (Fig. 1q, t-u).

### Sleep state specificity of single neurons drifts over time

Although the overall event frequencies in each vigilant state within LH subpopulations remained stable over time, we further investigated whether individual neurons maintained consistent state-specific activity or instead exhibited dynamic shifts. To address this question, we tracked and assessed the selectivity of single-neuron activity within each LH subpopulation over 7 days of longitudinal imaging (Fig. 1b). The stability of both the regions of interest (ROIs) and field of view (FOV) across days was validated (Supplementary Fig. 3).

**Fig. 2:**
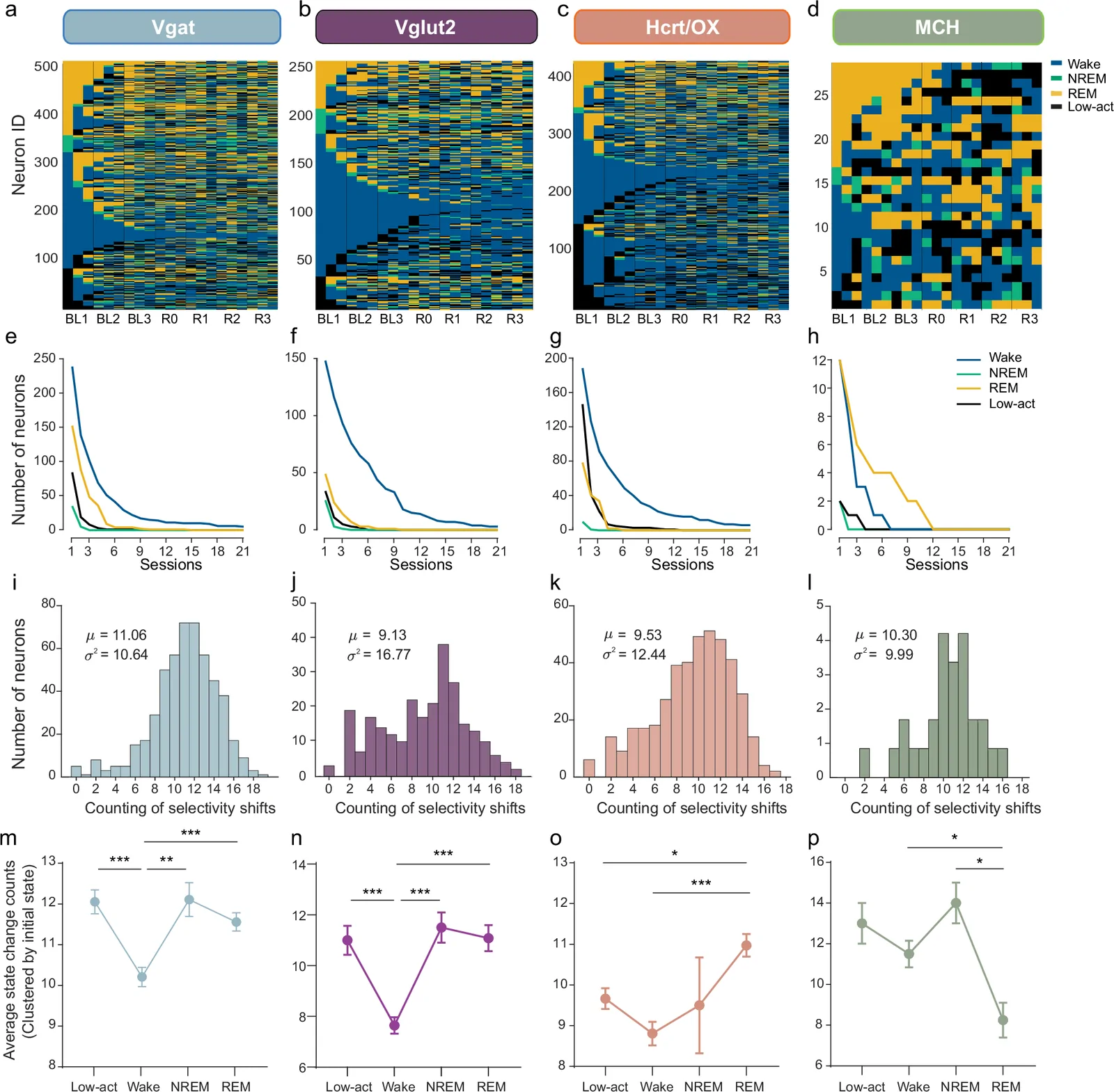
Single-neuron sleep state specificity decreases over time. **a–d** Heatmaps showing sleep-state specificity of individual neurons over 7 days (21 recording sessions) in LH-Vgat (*n* = 509 neurons, *m* = 6 mice), LH-Vglut2 (*n* = 257 neurons, *m* = 6 mice), LH-Hcrt/OX (*n* = 425 neurons, *m* = 6 mice), and LH-MCH mice (*n* = 28 neurons, *m* = 7 mice), respectively. Neurons are sorted based on their sleep-state selectivity in the first recording session, determined by their maximal event frequency during wake, NREM, or REM sleep. The selectivity of these neurons is tracked over time. Neurons that did not exhibit sufficient activity in any of the sleep-wake states were labeled as low-activity. **e–h** Survival curves depicting the proportion of neurons retaining their initial state-specific classification (wake, NREM, REM, or low-act) across sessions in each neuronal population. **i–l** Distribution of neurons according to the number of times their state selectivity changed across 21 sessions in LH-Vgat, LH-Vglut2, LH-Hcrt/OX, and LH-MCH mice, respectively. Each bar represents a shift frequency; μ and σ² indicate the mean and variance, respectively. **m–p** Average (mean ± SEM) frequencies of selectivity shifts within low-act-, wake-, NREM-, and REM-classified neuronal clusters. Neurons are classified according to their state selectivity in the first recording session. Sample sizes for each neuronal population apply to all corresponding panels in (**a–p**).*P < 0.05, **P < 0.01, ***P < 0.001. Statistical analyses and source data are provided in the Source Data file.

Interestingly, we observed that 58.0% of wake-active, 14.3% of NREM-active, and 57.9% of REM-active neurons remained in the same cluster between the first and second imaging sessions; however, a large majority of individual LH-Vgat neurons did not. Instead, their peak activity shifted to a different vigilant state between recording sessions, showing a dynamic reorganization of state-dependent single-cell activity over time (Fig. 2a; Supplementary Fig.4a). A similar pattern of state-dependent shifts was observed in LH-Vglut2, LH-Hcrt/OX and LH-MCH neurons (Fig. 2b-d; Supplementary Fig. 4b-d). Given that most neurons are not exclusively active in a single sleep–wake state, we further substantiated this reorganization by defining neuronal state selectivity using complementary analytical approaches: a threshold-based method that classified neurons as single-state– or multi-state–selective based on comparisons of activity across vigilance states (Supplementary Fig. 5a), and a category-independent analysis of Ca²⁺ event rates across all vigilance states without imposing fixed labels on individual neurons (Supplementary Fig. 5b,c; Movie S1).

To assess whether neurons display uniform or state-specific selectivity, a single-neuron selectivity index was calculated across sessions using a triangular state-space framework (see Methods) in LH-Vgat and LH-Hcrt/OX populations. Both neuronal types displayed graded rather than binary state selectivity, with most neurons occupying intermediate positions within Wake–NREM–REM space (Supplementary Fig. 6a, d; Movie S2). Selectivity showed moderate state bias for these cell types, while across-session variability revealed continuous drift in state representation (Supplementary Fig. 6b–f).

A survival analysis revealed that only a small fraction of neurons (4/509 in LH-Vgat, 1/257 in LH-Vglut2, 6/425 in LH-Hcrt/OX and 0/28 in LH-MCH) retained their initial state-dependent activity after 7 days of recording, suggesting a rapid reorganization of state-specific dynamics across all LH cell types recorded (Fig. 2e-h). On average, each neuron shifted its predominant state selectivity approximately 10 times across the 21 recording sessions (LH-Vgat: 11.06, LH-Vglut2: 9.13, LH-Hcrt/OX: 9.53 and LH-MCH: 10.30; Fig. 2i–l). To determine whether shifts in preferred state across sessions are stochastic or follow structured patterns, and whether the persistence of a minority of stable neurons exceeds statistical expectations, we computed transition probability matrices across consecutive sessions (see Methods). In all examined subpopulations, we observed that transition probabilities between clusters differed from fully randomized state assignments, which produced near-uniform probabilities (Supplementary Fig. 7a, c), indicating that these transitions were not purely stochastic. However, transition patterns were largely preserved after within-neuron permutation of session order (Supplementary Fig. 7a, b), suggesting that transitions do not follow strong temporal sequences but are likely driven primarily by overall state occupancy.

In line with this, we observed that neurons initially identified as wake-active, particularly those from the LH-Vgat, LH-Vglut2, and LH-Hcrt/OX populations, exhibited greater stability and underwent less frequent shifts in state-dependent activity compared to neurons classified as REM- or NREM sleep-active (Fig. 2m-o). In contrast, within the LH-MCH population, neurons originally classified as REM sleep-active displayed significantly higher stability than neurons classified as wake- or NREM-active (Fig. 2p). This is consistent with the observation that cells belonging to larger clusters tend to remain within these clusters for a longer period of time (Fig. 2e-h) and exhibit a comparatively higher probability of remaining in the same state (Supplementary Fig. 7).

Since wakefulness encompasses behaviorally heterogeneous substrates associated with different levels of arousal and physical activity that influence neuronal event rates, we examined whether variations in the proportion of active- versus quiet wake across sessions account for the observed neuronal shift of cluster selectivity. To do this, active wake episodes (i.e., wake with an EMG threshold; see Methods; Supplementary Fig. 8a) were excluded from the selectivity analysis using LH-Vgat mice as a representative population. Across all the recording sessions, active wake represented 70.8–97% of total wake time (Supplementary Fig. 8b). Eliminating active wake decreased the average neuronal activity during wakefulness as expected, while leaving neuronal activity during NREM and REM sleep unaffected (Supplementary Fig. 8c). The number of neurons classified as (quiet) wake-active neurons reduced accordingly and was redistributed to other clusters (Supplementary Fig. 8d). However, distinguishing active wake did not significantly alter the distribution or the average number of selective-state shifts per neuron compared with the original 3-stage classification (Mean number of transitions: 11.58 vs 11.06, Supplementary Fig. 8e). These results suggest that the observed dynamic reorganization is not attributable to behavioral heterogeneity within wakefulness. Hence, we considered a single state of wakefulness without distinction between quiet and active wake in all analyses.

### Sleep state-dependent neuronal populations remain stable over time

**Fig. 3:**
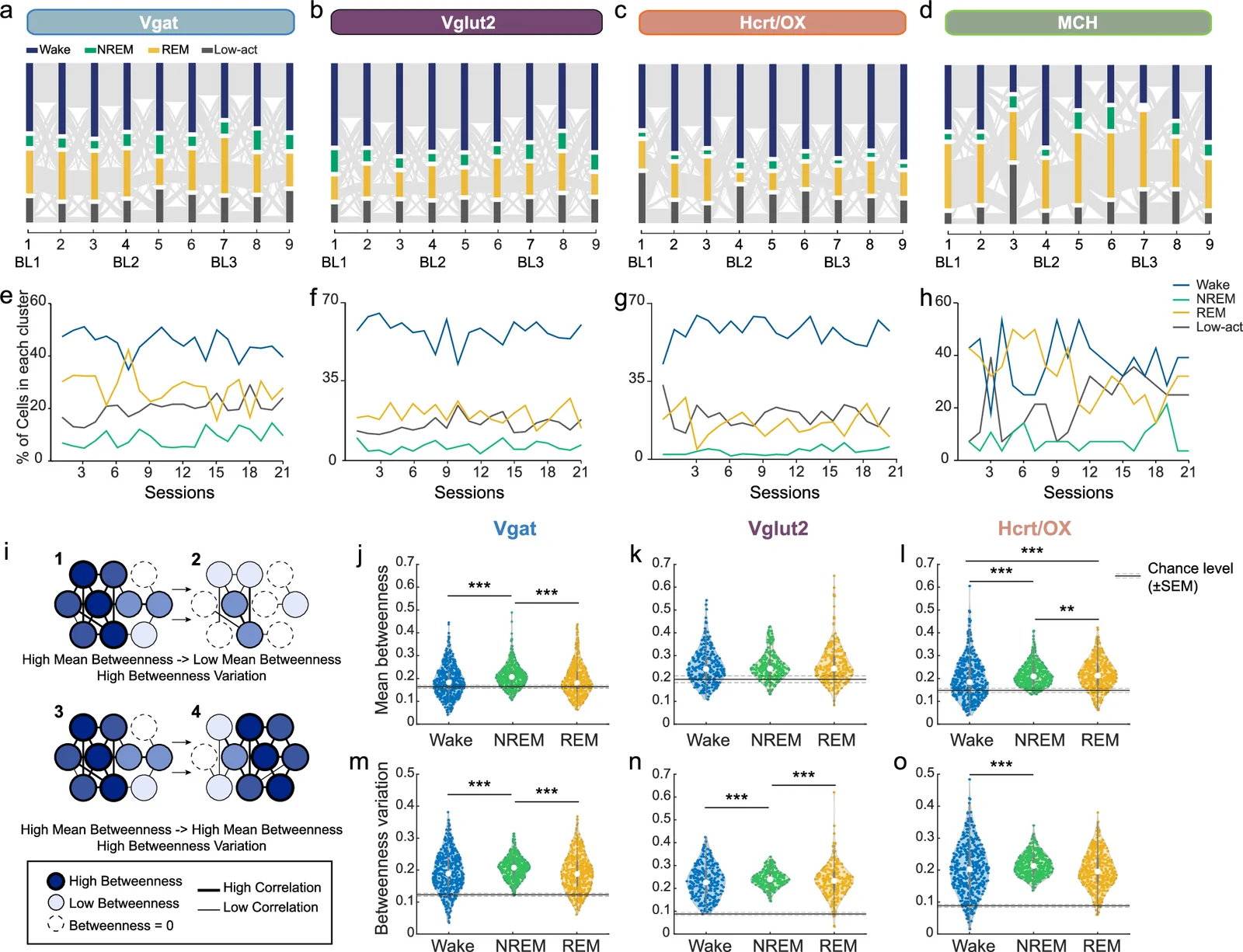
Sleep state-dependent neuron populations within the LH remain stable over time. **a–d** River plots showing the evolution of sleep-state selectivity in LH-Vgat (*n *= 509 neurons, *m* = 6 mice), LH-Vglut2 (*n* = 257 neurons, *m* = 6 mice), LH-Hcrt/OX (*n* = 425 neurons, *m* = 6 mice), and LH-MCH mice (*n* = 28 neurons, *m* = 7 mice) neurons across 9 baseline recording sessions. Each column represents the proportion of neurons classified as low-activity or selectively active during wake, NREM, or REM sleep. Light gray flows indicate transitions between subclusters over time. **e–h** Percentages of neurons assigned to each state-selective cluster across all 21 recording sessions in LH-Vgat, LH-Vglut2, LH-Hcrt/OX, and LH-MCH mice, irrespective of individual neuron selectivity. **i** Schematic illustrating the concept of betweenness centrality and betweenness variation. Betweenness centrality quantifies how often a neuron serves as a bridge within a network, representing its role in facilitating communication. Thicker lines indicate neurons with higher correlation. The color gradient reflects betweenness magnitude. Betweenness = 0 indicates connectivity with fewer than two neurons. Betweenness variation captures temporal fluctuations in connectivity, reflecting network reorganization over time. At the population level, changes in the overall mean betweenness (e.g., from **i1** to **i2**) or shifts in the distribution pattern of betweenness (e.g., from **i3** to **i4**) can both contribute to increased betweenness variation, suggesting shifts in the network’s structure or function. **j–l** Normalized betweenness value of active neurons during wake, NREM, and REM sleep in LH-Vgat, LH-Vglut2 and LH-Hcrt/OX mice. **m–o** Betweenness variation in wake, NREM, and REM sleep, assessing network stability in LH-Vgat, LH-Vglut2, and LH-Hcrt/OX mice. Violin plots display individual data points, with boxes indicating the median (white dots), interquartile range, and whiskers representing 1.5× the IQR. Solid and dashed lines represent the mean ± SEM of expected chance levels derived from shuffled datasets. Sample sizes for each mouse line are the same across all corresponding panels. **P < 0.01, ***P < 0.001. Statistical analyses and source data are listed in the Source Data file.

Next, we examined whether the distribution of wake-, NREM-, and REM sleep-active neurons within each recorded LH neuronal subtype remained stable over time at the population level, despite the rapid shifts observed at the single-cell level. Strikingly, although individual neurons frequently shifted their predominant activity between wake, NREM, and REM states (Fig. 3a), we found that the overall distribution of LH-Vgat neurons that are preferentially active during wakefulness (44.3 ± 4.4%; mean ± SD), NREM (8.6 ± 3.2%), and REM sleep (27.4 ± 5.8%) remained remarkably stable across all 21 recorded sessions (Fig. 3e). This population-level stability was also true for other LH subpopulations, including LH-Vglut2, LH-Hcrt/OX, and LH-MCH neurons (Fig. 3b-d, f-h), indicating that stable ensemble-level organization persists despite dynamic drifts in individual neuronal activity.

To further characterize the structure and variability of these neuronal activity patterns across sleep-wake states, we analyzed their functional interconnectivity between hypothalamic cell clusters by calculating the betweenness centrality across different sleep states (see Methods). Betweenness centrality is a metric used to assess the importance of a neuron within a network by measuring how often it acts as a ‘bridge’ between other neurons^31^. In our analysis, hypothalamic neurons with higher betweenness centrality showed a stronger functional correlation with other neurons (Fig. 3i). A network with a high betweenness indicates a more interconnected and structured organization, where specific neurons play a key role in facilitating communication. Additionally, betweenness variation reflects the stability of these connections over time; shifts in overall network betweenness (Fig. 3i, states 1 to 2) or specific connectivity patterns (Fig. 3i, states 3 to 4) can both lead to increased variation, suggesting fluctuations in the hypothalamus network structure.

When we computed this for LH-Vgat, LH-Vglut2, and LH-Hcrt/OX neuron activities across sleep-wake states (LH-MCH was excluded due to an insufficient number of recorded neurons), we found that betweenness centrality remained relatively comparable across wake, NREM, and REM sleep for these hypothalamic networks (Fig. 3j-l). Although we observed statistically significant differences between certain vigilance states, the distributions largely overlapped, with median centrality values remaining within a similar range and close to chance level. Notably, LH-Vglut2 (wake and REM), LH-Hcrt/OX (wake), and LH-Vgat (REM and NREM) populations exhibited positively skewed centrality distributions, with a small subset of neurons displaying relatively high betweenness values. This suggests the presence of limited hub-like neurons within a functional hypothalamic network. However, the majority of neurons exhibited centrality values not significantly different from chance (Fig. 3j–l), suggesting a limited degree of interconnectedness within these populations and an overall lack of persistent functional organization of these hypothalamic neuronal populations across sleep–wake states.

Furthermore, betweenness variation in LH-Vgat, LH-Vglut2, and LH-Hcrt/OX neuron populations also displayed a uniform distribution pattern across wake, NREM, and REM sleep states, despite some statistically significant differences between states (Fig. 3m–o). In all three LH subpopulations, median betweenness variation exceeded chance level, reflecting ongoing reconfiguration of the neuronal network over time, regardless of the behavioral state. These findings are consistent with the dynamic single-neuron drifts observed in Fig. 2 and further support a high degree of flexibility of state-dependent activity patterns across recurrent sleep-wake states.

To determine whether the neuronal activity of LH subpopulations is affected by sleep homeostasis, we subjected animals to a 4-h total sleep deprivation (SD) protocol (see Methods). In vivo imaging of single-cell activity was performed during the subsequent sleep rebound period (Fig. 1b and Supplementary Fig. 9a, b). In all recorded LH neuronal subtypes, SD led to increased NREM sleep duration and reduced wakefulness during the recovery period (Supplementary Fig. 9c), along with enhanced slow wave activity (SWA; 0.5–4 Hz) and increased peak-to-peak amplitude (Supplementary Fig. 9d). At the single-neuron level, SD induced statistically significant state-dependent changes in Ca²⁺ event rates across different LH subpopulations (Supplementary Fig. 9e). However, when event rates were averaged at the animal level (mean event rate across all neurons per animal), these differences were no longer significant, with the exception of reduced REM sleep-associated Ca²⁺ activity in LH-Vgat mice (Supplementary Fig. 9f). Furthermore, the integral of Ca²⁺ transients, as well as the proportion of neurons that active across sleep-wake states remained largely unchanged following SD (Supplementary Fig. 9g, h). Together, these findings indicate that population-level activities within LH neuronal subtypes remain stable after SD, while single-cell activities showed a heterogeneous, state-dependent modulation, suggestive of network resilience to sleep homeostasis.

### POA GABAergic neurons showed similar dynamics to LH neurons

**Fig. 4:**
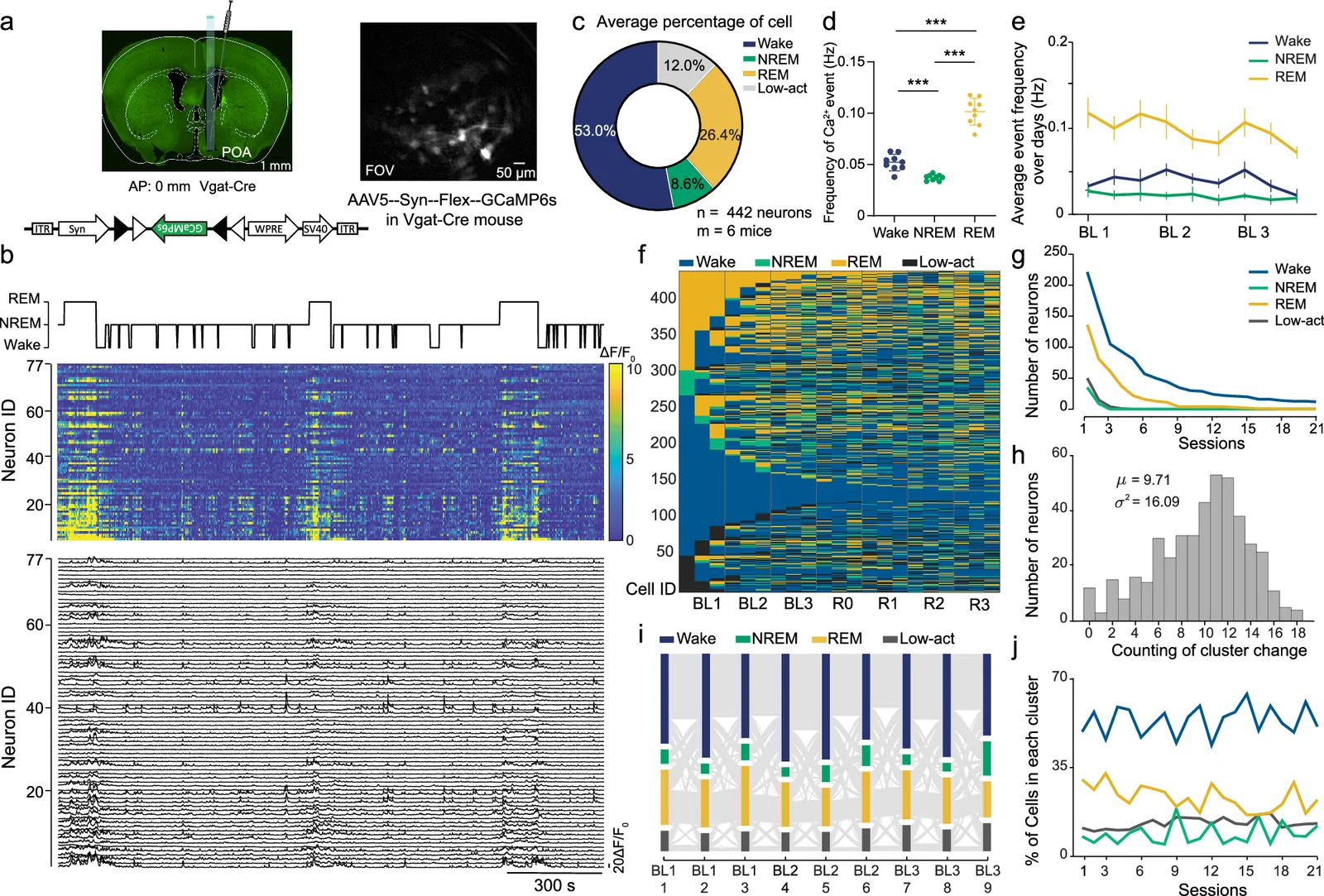
POA-Vgat neurons exhibit similar stability patterns across longitudinal sleep-wake cycles. **a** (Left) Expression of GCaMP6s in the preoptic area (POA) of Vgat-Cre mice with GRIN lens implanted in situ (Scale bar: 1 mm). (Right) Representative field of view of POA-Vgat neurons in vivo using a miniature microscope (Scale bar: 50 μm). **b** Representative hypnogram, color-coded and raw Δ*F*/*F*_0_ traces of POA-Vgat mouse. **c** Average percentages of POA-Vgat neurons (*n* = 442 neurons, *m* = 6 mice) classified as low-activity or showing maximal event rates during wake, NREM, or REM sleep across 3-day baseline recordings. **d** Session mean ± SEM of Ca²⁺ event frequency of POA-Vgat neurons during wake, NREM, and REM sleep in baseline recordings (*n* = 9 sessions). **e** Mean ± SEM Ca²⁺ event frequency of POA-Vgat neurons in each baseline recording. **f** Heatmap showing sleep-state specificity of individual POA-Vgat neurons over 7 days (21 sessions). Neurons are sorted by their state selectivity (wake, NREM, REM) in the first session; low-activity neurons are shown in black. **g** Survival curves depicting the retention of initial state-specific classification (wake, NREM, REM, or low-act) across sessions. **h** Distribution of neurons according to the number of times their state selectivity changed across 21 sessions in POA-Vgat neurons. *μ* represents the mean frequency, and *σ*² indicates the variance. **i** The evolution of sleep-state selectivity in POA-Vgat neurons across baseline recordings. **j** Aggregate percentage of neurons in each state-selective cluster across all recording sessions, regardless of individual neuron selectivity, in POA-Vgat mice. For (**e–j**), *n* = 442 neurons from six mice. ***P < 0.001. Statistical analyses and source data are listed in the Source Data file.

We next tested whether similar single-cell dynamics and population-level stability are also present in sleep-promoting neurons located in the anterior hypothalamus. To this end, we stereotactically injected AAV-Syn-flex-GCaMP6 into the preoptic area (POA) of Vgat-Cre (POA-Vgat) mice (Fig. 4a; Supplementary Fig. 1c). Ca^2+^ transients and EEG/EMG signals (Fig. 4b) were monitored using the same longitudinal imaging protocol described in Fig. 1b.

Lateral POA-Vgat neurons were predominantly wake-active, with 53% of neurons displaying maximal event rate during wakefulness; 26.4% were selectively active during REM sleep, 8.6% during NREM sleep, and 12% remained low-activity (Fig. 4c). Despite neuronal classification, POA-Vgat neurons displayed significantly higher Ca^2+^ event frequency during REM sleep, while remaining low during wakefulness and NREM sleep, with wake-related activity being higher than that during NREM. (Fig. 4d, e). Similar to LH neurons, we found that lateral POA-Vgat neurons exhibited a rapid loss of initial state selectivity, with only a small fraction of wake-selective neurons (12/442 cells) remaining in this cluster after 7 days of recording (Fig. 4f, g; Supplementary Figs. 4e and 5). On average, individual POA-Vgat neurons shifted their state selectivity 9.7 times across the 21 recording sessions (Fig. 4h). Despite this dynamic reorganization at the single-cell level, the overall distribution of neuronal activity profiles across sleep-wake states remained stable at the population level over time (Fig. 4i, j). These results suggested that the observed drift of state-selectivity is not confined to specific hypothalamic subregions or neuronal subtypes, but rather reflects a broader organizational feature of the hypothalamic networks examined here.

### DZP increased POA-Vgat neuron activity and their selectivity for NREM sleep at ZT12

**Fig. 5:**
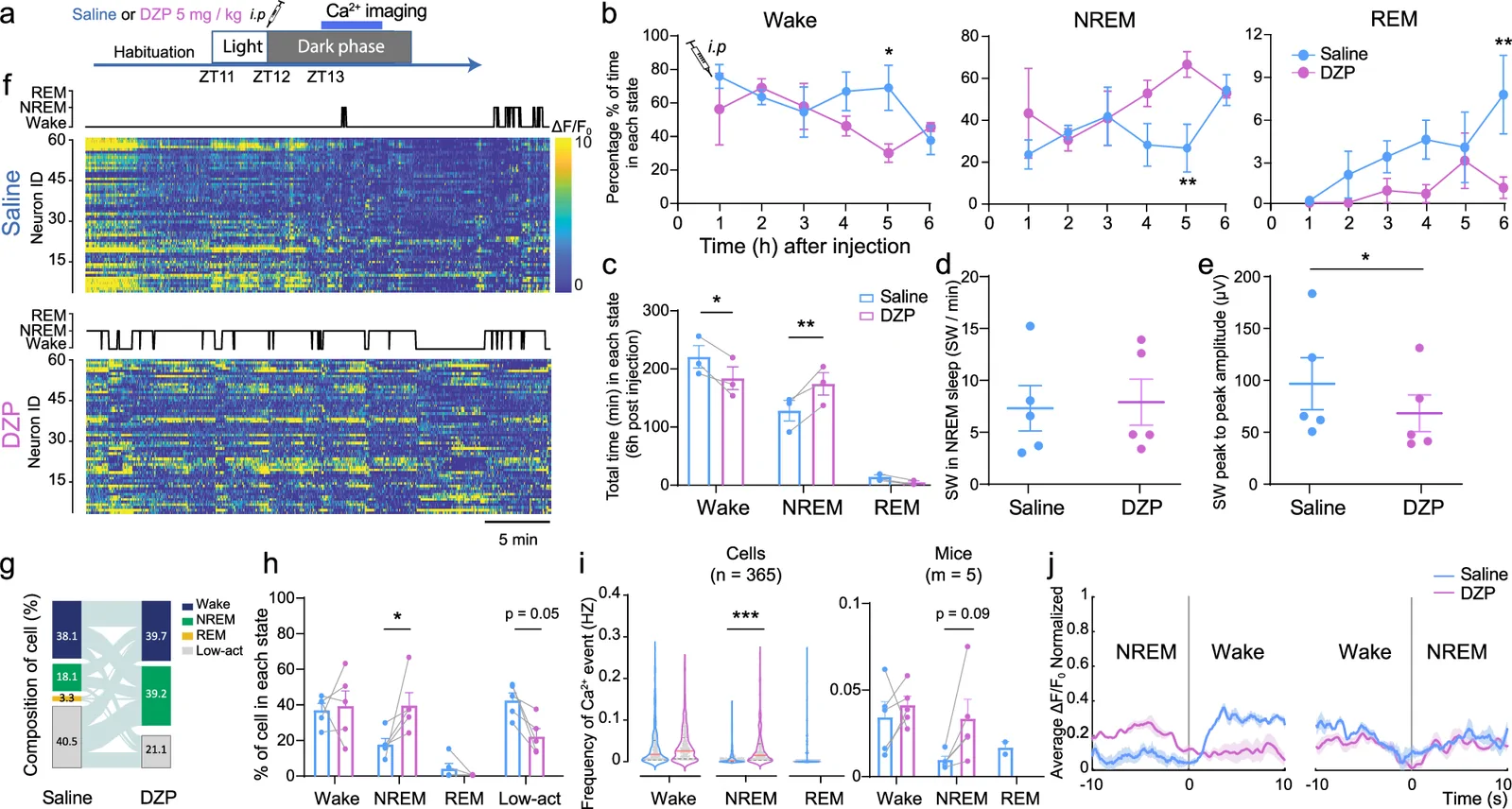
Diazepam (DZP) injection at ZT12 increases POA-Vgat neuronal activity and selectivity during NREM sleep. **a** Experimental schematic. Saline or DZP (5 mg/kg) was administered intraperitoneally at ZT12 (20:00), with calcium imaging recordings initiated within 1-h post-injection. **b** Time course of wake, NREM, and REM sleep percentages over 6 h following saline or DZP treatment. Data are presented as mean ± SEM (*n *= 3 mice; repeated measures in both groups). **c** Cumulative duration of wake, NREM, and REM sleep within 6 h post-saline or DZP treatment (*n* = 3 mice; repeated measures). Average (mean ± SEM) frequency (**d**) and peak-to-peak amplitude (**e**) of NREM slow waves under saline and DZP conditions (*n* = 5 mice; repeated measures). **f** Representative hypnograms and color-coded Δ*F*/*F*₀ traces from saline- and DZP-treated mice. **g** Composition and transition of state-selective clusters from saline to DZP conditions (*n* = 365 neurons, *m* = 5 mice). **h** Quantification of the percentage of neurons classified as low-activity or preferentially active during wake, NREM, or REM sleep under each condition (*n* = 5 mice; repeated measures). **i** Ca²⁺ event frequency (Hz) across sleep-wake states, averaged per neuron and per animal (*n* = 365 neurons, *m* = 5 mice). REM sleep was diminished under DZP condition. **j** Average (mean ± SEM) Ca^2+^ traces at state transitions: from NREM to wake and from wake to NREM (±10 s around transition onset), *n* = 365 neurons from *m* = 5 mice. In bar plots, data are presented as mean ± SEM; dots represent individual animals, and connecting lines indicate paired measurements before and after treatment. In violin plots, data are presented as median (red solid line) with 25th and 75th percentiles (gray dashed line). For (**g**–**j**), *P < 0.05, **P < 0.01, ***P < 0.001. Statistical analyses and source data are listed in the Source Data file.

To determine whether pharmacologically-induced changes in sleep architecture alter the state-dependent activity of lateral POA-Vgat neurons, we next recorded their response to Diazepam (DZP), a GABAergic sedative known to enhance NREM sleep^32^. As expected, a single dose of intraperitoneal (i.p) DZP (5 mg/kg) at ZT12, when sleep pressure is minimal, extended the duration of NREM sleep at the expense of wakefulness (Fig. 5a-c) and significantly reduced the peak-to-peak amplitude of slow waves without altering their number, compared to saline-injected control conditions (Fig. 5d, e). Importantly, we found that DZP significantly increased the proportion of lateral POA-Vgat neurons activated during NREM sleep as compared to the control group, by either recruiting previously low-activity neurons or switching a large fraction of the wake-active neurons into the NREM-active category (Fig. 5g, h). In addition, DZP significantly elevated the mean Ca²⁺ event rate of lateral POA-Vgat neurons during NREM sleep calculated at the cell level and showed a strong trend toward significance when averaged at the animal level (Fig. 5f, i). Furthermore, NREM-related activity was suppressed during transitions from NREM sleep to wakefulness following DZP administration compared to Saline (Fig. 5j).

In contrast, when DZP was administered at ZT0, which corresponds to a time of elevated sleep pressure (Supplementary Fig. 10a–c), we found that NREM-associated Ca²⁺ transients and the overall distribution of wake-, NREM-, and REM-active neurons did not differ significantly from controls (Supplementary Fig. 10d, e), aside from an increase of inactive neurons. Ca²⁺ event frequency during REM sleep was reduced following DZP treatment (Supplementary Fig. 10f, g); however, no significant change in NREM-related neuronal activity was observed. Notably, the characteristic single-cell dynamics and population-level stability remained intact across 3 consecutive days of DZP administration (Supplementary Fig. 10h, i).

Finally, we further investigated whether DZP similarly modulated neuronal activity in the LH, by selectively imaging LH-Vgat and LH-Hcrt/OX neurons upon administration of a single dose of DZP (5 mg/kg) at either ZT0 or ZT12, as described above (Supplementary Fig. 11). In LH-Vgat neurons, we found that DZP treatment resulted in a significant reduction in the proportion of REM-active neurons at ZT0, and to a lesser extent at ZT12, accompanied by a decrease of Ca²⁺ event frequency during REM sleep (Supplementary Fig. 11a-f). However, note that DZP markedly suppressed REM sleep duration in almost all animals. In contrast to POA-Vgat neurons, LH-Vgat neurons showed only a directional but non-significant increase in NREM-active neurons and NREM-associated Ca²⁺ activity at ZT12. LH-Hcrt/OX neurons exhibited minimal response to DZP at both time points (Supplementary Fig. 11g-l).

In summary, we showed that DZP selectively enhanced the ratio of NREM-active neurons in POA-Vgat neurons at ZT12, but not at ZT0, with a non-significant trend also observed in LH-Vgat neurons. An increase in low-activity neurons was observed following DZP administration at ZT0 in POA neurons and, to a lesser extent, in LH populations. These findings indicate a region- and state-dependent sensitivity to pharmacological modulation.

## Discussion

These results indicate that although individual hypothalamic neurons exhibit dynamic, state-dependent shifts in their encoding of sleep–wake states, the overall organization within each population remains stable. This shift is similar to the representational drift^33^, described in highly plastic brain structures including the hippocampus, visual and auditory cortex, where individual neuron responses to specific stimuli or behavioral tasks fluctuate over time, while the overall population-level pattern remains consistent^34–38^. Such a mechanism is thought to support functional stability through redundancy and adaptability during learning or sensory perception^33,39,40^. Our findings extend this mechanism to control innate behavior, possibly to ensure the stability of sleep-wake states upon external, or internal, challenges such as sleep homeostasis or pharmacological treatment. This is consistent with the relatively minor impact of experimental ablation of these hypothalamic systems on sleep-wake architecture^41–43^.

This drift in the cellular substrates of sleep and wakefulness likely reflects the heterogeneous inputs to Vgat, Vglut2, Hcrt/OX and MCH neurons^44–46^, as well as the molecular heterogeneity of hypothalamic neuronal populations recorded^13,30,47^. Indeed, accumulating studies suggest a higher functional heterogeneity amongst hypothalamic circuits, in particular LH-Hcrt/OX cells^30^, than previously thought^17,48^. Activity preceding sleep onset may shape subsequent neuronal ensemble recruitment^49^, while the sparse intrinsic connectivity among LH neurons could further support this functional flexibility in sleep regulation^50^. Beyond input heterogeneity, we hypothesize that these cellular dynamics reflect system plasticity that contributes to the reconfiguration of ensemble dynamics underlying sleep-wake regulation, as described for synaptic and dendritic spine plasticity^51–53^, activity-dependent gene expression^54,55^, protein synthesis and phosphorylation^56,57^, and axonal bouton dynamics^58,59^ across distributed brain networks.

Both sleep deprivation and diazepam increased NREM sleep, however, only DZP was associated with a reorganization of cellular activity patterns, including a recruitment of NREM active neurons and an increase of neuronal activity during NREM sleep, possibly resulting from a shift in excitation–inhibition balance^32,60^. Given the broad action of DZP in enhancing GABA_A_ receptor–mediated inhibition, these changes likely reflect distributed network modulation that involves local cellular and circuit disinhibition. Notably, the DZP effect was most pronounced under low sleep pressure, suggesting that selectivity of sleep-wake clusters show different sensitivity to pharmacological modulation depending on physiological state.

While GCaMP-based calcium imaging enables longitudinal monitoring of identified neuronal populations, it represents an indirect readout of spiking activity^61^. Calcium signals may not fully capture sparse firing and reflect only a subset of neurons within the recorded region. Nevertheless, the consistent state-associated drift observed across animals and experimental conditions support the existence of such ensemble dynamics in sleep–wake regulation. Advances in high-throughput electrophysiological approaches with single-cell resolution will be instrumental in further clarifying the relationship between spiking activity and ensemble-level encoding, and may provide additional mechanistic insight into the present findings.

Altogether, these results highlight a fine-tune balance between genetically encoded stability (or rigidity) and functional flexibility of hypothalamic neurons regulating sleep–wake states. Such flexibility likely supports the brain’s ability to maintain predictable and restorative sleep while adapting to changing internal and external demands^62,63^. In this context, a flexible encoding strategy may allow sleep-regulating cells to respond to changes, such as hormonal fluctuations, stress, temperature, or metabolic demands^64,65^, and to support multifunctional roles such as thermoregulation and energy balance^66–68^ while maintaining a stable sleep-wake architecture. Furthermore, this functional shift in the cellular encoding of sleep-wake states may ensure a network-level resilience of cellular circuits underlying homeostatic functions such as sleep in case of neuronal damage, dysfunction^69,70^ or disease. Future studies will be meaningful to determine whether similar ensemble dynamics extend to other hypothalamic-regulated behaviors, such as feeding and drinking, and whether such drift represents a more general principle of circuit organization underlying other innate behaviors.

## Methods

### Animal subjects

Adult male C57Bl6JRj, Vgat-ires-Cre (Strain #016962), Vglut2-ires-Cre (Strain #028863) and MCH-ires-Cre (Strain #014099) mice, aged 8–24 weeks, were used in the neuronal longitudinal tracking study. Both male and female C57BL/6JRj and Vgat-ires-Cre mice were included in the pharmacology study. Before surgeries, the mice were group-housed in ICV cages (Green Line, Techniplast), while post-surgery was single-housed in custom, open polyacryl cages (18 × 29 cm). In both conditions, animals stayed at constant temperature (22 ± 1 °C) and humidity (30–50%), under a circadian cycle of a 12-h light-dark cycle with lights on at 08:00. Food and water were available ad libitum. All procedures were conducted following protocols and guidelines approved by the Veterinary Office of the Canton of Bern, Switzerland (license numbers BE 129/2020, BE122/2023 and BE57/2024).

### Viral targeting

In this study, we utilized both male and female mice, aged between 8 and 12 weeks at the start of the experiments, for in vivo calcium imaging to examine specific neuron sub-populations in the brain. The mice were initially anesthetized using 5% isoflurane and subsequently maintained at a level of 1.5–2% throughout the procedure. During the experiments, each mouse was fixed in a digital stereotactic frame, and body temperature was maintained at 37 °C using a feedback-coupled heating device (Panlab/Harvard Apparatus). Eye hydration was preserved with Bepanthen ointment (Bayer), and pain management was handled using 100 µl of lidocaine (1%) locally and a subcutaneous injection of Carprofen (10 mg/kg) administered 30 min before surgery. The preparation for surgery included shaving the hair and disinfecting the skin over the skull with 70% modified ethanol and betadine. Following a precise skin incision, alignment of anatomical landmarks (bregma and lambda) was performed. A small hole was then drilled using a pneumatic dental drill (Foredom, K1030 Portable) for targeted injections into the brain.

Specific targeting involved unilateral injections into the lateral hypothalamus (LH) and the preoptic area (POA) to reach various neuronal types. To target GABAergic, glutamatergic, and MCH neurons in the LH, we administered unilateral injections of 0.6 µl of recombinant AAV5-Syn-flex-GCaMP6s (Addgene, 100845) into Vgat-Cre, Vglut2-Cre, and MCH-Cre mice, respectively. For targeting hypocretins/orexins neurons in the LH, 0.6 µl of AAV1/2-hORX-GCaMP6s-hGHP(A) (VVF ETHZ) was injected into C57Bl6JRj mice. The coordinates used for LH were from Bregma anterior-posterior (AP): −1.35 mm, medial-lateral (ML): ±0.9 mm, dorso-ventral (DV): −5.30 mm. Additionally, to target GABAergic neurons in the POA, Vgat-Cre mice received 0.6 µl injections of AAV5-Syn-flex-GCaMP6s (Addgene, 100845) at AP: 0 mm, ML: +0.7 mm, DV: −5.2 mm. The viral infusions were performed at a maximum rate of 100 nl/min using a micro-infusion pump (PHD Ultra, Harvard Apparatus) through a 28 G stainless steel cannula (Plastic One).

### GRIN lens and EEG/EMG electrodes implantation

Two weeks after virus injection, mice were anesthetized with an intraperitoneal (i.p.) injection of a mix containing medetomidine (0.5 mg/kg), midazolam (5 mg/kg) and fentanyl (0.05 mg/kg) in sterile NaCl 0.9% (MMF-mix). Analgesia was achieved by local application of 100 µl of lidocaine (lurocaine, 1%) and subcutaneous (s.c.) injection of Carprofen (10 mg/kg). Dexamethasone (0.5 mg/kg) was injected intramuscularly into the quadriceps to prevent potential inflammation that would be caused by the drilling and implantation. The animals were then shaved and placed in the stereotactic frame as described above. The skull bone was cleaned with 4% hydrogen peroxide and saline to remove remaining tissue. To ensure precise and controlled placement, the GRIN lens (diameter: 0.6 mm; length: 7.3 mm, Inscopix) was descended into the LH (−1.35 mm AP; +0.9 mm ML; −5.35 mm DV) or POA (0 mm AP; +0.7 mm ML; −5.25 mm DV) at 8 µm/s for the first two-thirds of DV and then 0.8 µm/s for the final third with the help of stereotaxic motorized manipulator (Scientifica IVM Single). The lens was then fixed to the skull with a three-component dental cement (C&B-Metabond, Parkell Inc.).

For polysomnographic recordings, four EEG electrodes made of stainless-steel screws were placed into the skull to record EEG signals (bilaterally into the frontal regions and parietal cortices, avoiding lens implanted region) and two EMG bare-ended wire loops were sutured to the trapezius muscle of the neck to record muscle activity. The EEG/EMG implants were then anchored to the skull with dental cement (Paladur, Patterson dental) and a cement plateau was built around the lens. The top of the GRIN lens was cleaned and covered with a plastic lid. To end the anesthesia, mice were injected subcutaneously with an antagonist mix (ABF-mix) containing atipamezole (2.5 mg/kg), buprenorphine (0.1 mg/kg) and flumazenil (0.5 mg/kg). After the surgery, mice underwent a recovery period with three days of subcutaneous administration of analgesic buprenorphine (0.1 mg/kg).

After a minimum of two weeks of recovery, the animals were anesthetized again with isoflurane and placed in the stereotactic frame. Black nail polish was applied around the lens to prevent light propagation during the imaging. The optimal imaging focus plane for the micro-endoscope (nVista 3, Inscopix) was determined. The baseplate attached to the micro-endoscope was fixed with UV glue and cement onto the existing cement layer, then black nail polish was applied around the cement again. The EEG/EMG cables were plugged into the EEG/EMG implants, taped with parafilm, and covered by aluminum foil. A tethered dummy micro-endoscope was connected to the baseplate for habituation to the recording device. After the procedures, the mice returned to their home cages. The cables from the dummy miniscope and EEG/EMG electrodes were securely taped to the top of the cage. This setup ensured that the animals could move freely within their environment.

### Calcium imaging and polysomnography data acquisition

After an additional week of recovery and habituation to the cables, the mice were restrained to remove the microscope dummy and attach the real miniature microscope to the baseplate 1-2 days before the start of the experiment. Mice were recorded using a longitudinal protocol consisting of three days of baseline (BL1-3), 4 h of sleep deprivation followed by acute sleep recovery (R0), and three days of chronic sleep recovery (R1-3) (Fig. 1b). Each day included three recording sessions, with each session lasting approximately 30 min. Before each recording session, mice were thoroughly acclimated to the experimenter and the recording environment to minimize stress.

At the beginning of the longitudinal calcium imaging protocol, the focus, gain, and illumination power of the microscope were carefully optimized to ensure clear visualization of neuronal activity as well as background vascular landmarks. For each animal, the focus of the miniature microscope remained unchanged throughout the study, and the same field of view (FOV) was consistently imaged across all sessions to track the same neurons over time. Corresponding FOVs across seven consecutive days were aligned using stable vascular landmarks to ensure accurate registration of the same neuronal population over time. Minor adjustments to the light power were occasionally made during later sessions to compensate for photo bleaching. The miniature microscope remained attached to the mouse throughout the entire recording period to ensure the stability of the FOV across all sessions.

Calcium imaging data were collected at a rate of 10 frames per second using Inscopix Data Acquisition Software (IDAS, version 1.4.0) with typical excitation light intensities ranging from 0.6 to 1.2 mW/mm² (30–60% of maximum power, nVista 3, Inscopix). Simultaneously, EEG and EMG signals were recorded using an AM Systems 3500 amplifier, sampled at 512 Hz, and digitized with a National Instruments USB X DAQ device. To synchronize the calcium imaging data with the polysomnography recordings, binary timestamps for each frame from the miniscope were integrated with the AM systems. Animal behavior was recorded as a reference using a FireFly MV camera (Point Grey Research) at 15 frames per second. All EEG, EMG, behavioral video data, and synchronization signals were compiled and analyzed using SleepScore software (ViewPoint, V1.9.1).

During the baseline sleep phase (days 1–3) and the sleep deprivation recovery phase (days 5–7), the morning recording session began at around 10:00 A.M. (ZT2), followed by two additional sessions at 3:00 P.M. (ZT7) and 5:00 P.M. (ZT9). Sleep deprivation was carried out from 8:00 A.M. to 12:00 P.M. (ZT0-ZT4) for a total of 4 h. The first recording session after sleep deprivation started as soon as the animal entered the first long NREM sleep bout, with subsequent sessions at 3:00 P.M. and 5:00 P.M. Data acquisition was performed while the mice remained in their home cages. The experimenter entered the room at least 30 min before the start of each session to allow the mice to naturally settle. Recordings began once the animals reached a stable NREM sleep state. Due to the large amount of data streaming, individual imaging sessions were limited to approximately 30 min.

### Sleep deprivation

Total sleep deprivation was performed manually using a gentle handling technique. Mice remained in their home cages with real-time EEG and EMG monitoring. When the experimenter observed signs of the mouse entering NREM sleep, gentle stimuli such as touching the tail or body with a cotton swab, offering enrichment materials for exploration, or adding fresh bedding to prompt nesting behavior—were used to wake the mouse. The aim was to keep the mouse awake without causing stress. This approach has been widely employed in sleep studies and has been shown to have minimal impact on stress hormone levels in a short period, particularly corticosterone, thereby minimizing confounding stress effects during the deprivation process^71,72^.

### Polysomnography data scoring

Three vigilance states—wakefulness, NREM sleep, and REM sleep—were identified using established criteria based on EEG and EMG characteristics, as outlined before^73^. Briefly, wakefulness was characterized by low-amplitude, desynchronized EEG patterns and high-activity EMG signals featuring phasic bursts. NREM sleep was defined by synchronized EEG oscillations within the slow- and delta-frequency bands (0.5–4 Hz) that exhibited high amplitude and low frequency, coupled with significantly reduced EMG activity. REM sleep was identified by pronounced theta oscillations (6–9 Hz) in the EEG, or an increased presence of desynchronized, high-frequency oscillations, alongside an almost complete absence of EMG tone, except for brief muscle twitches. Micro-arousals during NREM sleep were determined by the presence of at least one second of cortical fast rhythms and EMG bursts. All recordings were analyzed at an epoch length of one second using the Matlab-based SlipAnalysis software (v.2.9.98, Lyon Neuroscience Research Center). The scoring data were compiled into a hypnogram for further analysis, allowing alignment of neuronal activities with vigilance states.

### Automatic single slow wave detection

Individual slow waves were detected during NREM sleep epochs based on the single EEG channel of each mouse using the SWA-MATLAB toolbox^74^, with detection parameters adjusted for mice according to the settings described by Panagiotou et al.^75^. Slow waves were identified in various conditions, including baseline sleep, sleep recovery after sleep deprivation, and sleep following NaCl or DZP treatment. Briefly, the EEG data underwent initial processing to compute the negative envelope, which was then filtered within the 0.5–4 Hz frequency band using a Chebyshev Type II filter to isolate slow wave frequencies. Subsequent analysis involved consecutive zero-crossing detection, where transitions from negative to positive and vice versa were identified to mark potential starts and ends of slow waves. For a waveform segment to be classified as a slow wave, it had to meet two criteria: the duration between its negative-going zero-crossing and subsequent upward zero-crossing needed to be between 100 ms and 1 s, and the peak negative amplitude had to exceed a threshold of three standard deviations above the median amplitude of all negative peaks recorded. This approach provides a structured process for identifying slow wave events based on specific amplitude and duration parameters, helping to mitigate potential individual differences due to electrode reference type, distance to those references, and electrode depth, which could affect the recorded amplitude.

### Calcium traces extraction

Stacks of raw calcium imaging recordings were preprocessed to crop the redundant imaging area, correct time-invariant pixels, or reconstruct frames lost during the acquisition, and were then adjusted for motion artifacts in the *x*-*y* plane using Inscopix Data Processing software (Inscopix, V1.9.1). Subsequently, the image stacks were exported to Fiji (ImageJ, version 1.53t), where the imaging background was subtracted. A common set of regions of interest (ROIs) corresponding to individual neurons was manually defined for each mouse by scrolling through all recording sessions. The average calcium fluorescence intensity within each ROI was then extracted from each recording session and exported using Fiji, providing a single time-series of raw fluorescence.

Raw traces are further processed and normalized using custom-made scripts (Matlab R2019b, MathWorks) as described before^49^. In brief, while only minor signal loss due to photobleaching was observed, to adjust for potential changes in GCaMP6 fluorescence within a session, an (single) exponential curve was fitted and subtracted from the raw signal of each cell during each session to ensure consistency in the fluorescence measurements. The fit was computed over the entire length of a recording session, and the correction consequently applied to the entirety of the recording. Subsequently, *F*_0_ was determined using Gaussian mixture modeling, where the observed intensity values from each cell in each session were approximated as a mixture of Gaussian distributions via an expectation-maximization procedure. The mean of the distribution containing the lowest values was identified as the *F*_0_ for each neuron and session. Δ*F*/*F*_0_ was then calculated based on bleaching-corrected calcium traces and estimated F_0_, and normalized using envelope normalization. Normalized traces are referred to as ‘Δ*F*/*F*_0_’ throughout the paper.

### Image field and ROI longitudinal stability analysis

To address potential concerns regarding possible FOV shifts and longitudinal ROI registration stability across days, we performed two validation analyses based on maximum projection images from each recording session.

First, we computed a shuffle-based estimate of longitudinal ROI mismatch probability. For each ROI and pair of recording sessions, the local image similarity was quantified as the pixelwise correlation between the image content contained within the same ROI footprint across days. To estimate the probability of random mismatching, the local image correlation of the true longitudinal ROI match was compared against correlations obtained from shuffled ROI pairings between sessions. False-match probability was defined as the fraction of shuffled ROI comparisons producing equal or greater similarity than the true ROI match.

Second, consecutive-day image displacement was quantified by estimating the rigid translational shift required to align imaging fields between consecutive recording sessions. For each pair of consecutive days, translational registration was performed on the projection images, and the resulting x/y translation vectors were used to compute the total displacement magnitude in pixels. Displacement values were then pooled across animals to assess longitudinal field-of-view stability over time.

To further cross-validate neuronal and FOV stability across days, a subset of the longitudinal imaging dataset was independently analyzed using CaliAli^76^ in MATLAB R2025b. Alignment quality was assessed using the CaliAli ‘Evaluating Alignment Performance’ functions. CaliAli-derived neuronal components were compared across 7-day recordings before and after inter-session alignment. An intra-session control was generated by dividing a single recording session into seven segments, providing a positive control for within-session stability and allowing long-term cross-day tracking to be compared with short-timescale stability. FOV consistency was assessed using blood-vessel similarity scores. For this analysis, the intra-session control served as a positive control, whereas a between-animal control, generated from recording sessions belonging to different animals, served as a negative control. Residual non-rigid misalignment was further estimated relative to neuronal size. This analysis was used only as an independent validation and was not used for downstream dF/*F*_0_ extraction or state-selectivity analysis.

### Artifacts detection and removal

The Δ*F*/*F*_0_ traces were further pre-analyzed for the removal of any leftover movement artifacts. The measured raw EMG signal was used to determine sharp movements and ignore the corresponding frames. For this purpose, the EMG was thresholded on five times its mean + standard deviation, and the parts of the recordings that corresponded to peaks in absolute value higher than the aforementioned threshold were marked as motion artifacts and ignored by the analysis. Furthermore, since sudden movements of the field of view with respect to the drawn ROI usually correspond to brief, sharp decreases in the ROI average intensities, causing sudden decreases in the signal, as a second step of motion artifact detection, the ROI traces were averaged to obtain an overall population signal. Then, a negative peaks detection using Matlab’s ‘findpeaks’ function was run on the average trace, and the frames corresponding to negative peakswere removed from the analysis.

### Ca^2+^ event detections

Calcium transient events (‘events’ in short) were detected using custom software made in our group, with Matlab 2019b. Each ROI Δ*F*/*F*_0_ trace was analyzed separately after motion correction and artifacts detection. Traces were first upsampled via interpolation to be smoother with cubic spline interpolation (spline function in Matlab). The baseline median ‘M’ and noise level ‘E’ for each trace were estimated by computing the median and standard deviation of the signal in three iterations, the points exceeding two times the standard deviation were excluded from the estimation of M and E in the next iteration. Candidate calcium events were identified via the use of the findpeaks function in Matlab, using twice the estimated M + E as threshold for minimum peak height, and M + E as minimum peak prominence. A window of 6 s was considered before every identified peak and the maximum of the second derivative of the signal taken as the starting point of the candidate Calcium Event. The end point of the candidate event was taken as the point after the peak where the signal would return to the ‘local baseline’ value, computed as the average of the signal over the 6 s preceding the event start. The characteristics of the candidate Events, such as duration, peak amplitude, half-width, integral, duration of rise and decay phase were computed. Each measured characteristic was then compared with the minimum and maximum physiological characteristics, and if not respecting these, it was excluded. The candidate events were then filtered and confirmed as Ca^2+^ events if their measured characteristics satisfied some minimum physiological parameters. Physiological parameters used for filtering candidate events were: Minimum Duration of an Event = 1 [s]; Maximum Duration of an Event = 20 [s]; Maximum Ratio between rise and decay duration = ⅓; Minimum Integral = 1 [a.u. (Δ*F*/*F*) * s]; Minimum Peak Amplitude = 2 [a.u. (Δ*F*/*F*)] for Hcrt/OX and Vglut2 neurons, or = 1 for Vgat neurons.

### State selectivity and stability analysis

The calcium traces were analyzed for each mouse and each session separately, to determine their state selectivity and their changes in different recording sessions and across multiple days. Calcium transients were identified as described above and separated into each different wake-sleep cycle state (wake, non-REM, and REM), according to where the calcium peak would occur. States with a duration shorter than 20 s were removed from the analysis, as considered unstable.

The frequency of calcium transient events (frequency of events) was then computed per cell, for each state, as the number of events happened during all stable states of a certain type, divided by the total duration of the stable states of that type. Cells with event frequency in the lower 25 percentile of all cells across three states, were considered separately as ‘inactive’ or ‘low-activity’ cells, and given the lack of activity, their state selectivity could not be estimated. For the remaining active neurons, two approaches were used to assign state selectivity: 1) Maximal activity assignment: Each neuron was initially assigned to the state in which it exhibited the highest event frequency. 2) Threshold-based selectivity: To determine state specificity more rigorously, Ca²⁺ event rates were compared across states. A neuron was considered selective for a given state (or multiple states) if its firing rate in that state was significantly higher than in the others, with the difference exceeding a noise threshold defined as half the standard error of the mean event rate. Based on these criteria, neurons were classified as single-state selective (e.g., Wake, NREM, or REM), multi-state selective (e.g., Wake & REM), non-selective (unspecific) if no dominant selectivity was observed, or low-activity, if a neuron was either fully inactive or not active enough to be categorized during that recording session.

### Triangular state-space framework of single-neuron activity, selectivity, and variability

Single-neuron activity across wake, NREM, and REM sleep was quantified as the mean Ca²⁺ event frequency per state for each recording session. To enable longitudinal comparison, activity values were normalized for each neuron to its maximal event rate observed across all sessions and states. For each neuron and session, the Euclidean distance between the activity vector and each state vertex was computed. Smaller distances indicate stronger selectivity toward a given state. An absolute selectivity index was derived as the minimum distance across the three states. Then a normalized state selectivity index was calculated as (1):

1 $${\rm{Selectivity}}=1-({\rm{distance}}/\sqrt 2)$$

where √2 represents the maximal possible distance within the triangular space.

Normalized activity vectors and selectivity index of each neuron across sessions were represented in a three-dimensional state space defined by the canonical vertices Wake = [1,0,0], NREM = [0,1,0], and REM = [0,0,1]. Across-session stability was assessed by computing the mean distance to each state vertex across sessions and quantifying variability as the standard error of the distance across sessions. This metric reflects the temporal stability of state selectivity. Chance-level selectivity and variability were estimated by assigning random activity vectors to each neuron independently for each session within the same three-dimensional state space. Distance, selectivity, and variability metrics were recomputed using identical procedures. The mean of the randomized distribution served as the baseline reference for comparison with empirical data.

### Probability of state-selectivity transitions

State-selectivity dynamics across longitudinal recording sessions were evaluated by computing the conditional probability of transitions between state-selective categories for each neuron. For each cell, state identity was assigned independently for every session using the maximal activity assignment method described above. Only valid state labels within the defined state space were included in the analysis. Transitions between consecutive sessions were identified for each neuron by comparing state labels at session *t* and session *t *+ 1. A transition matrix was constructed by counting all observed state-to-state transitions across sessions. Transition counts were normalized by the total number of transitions originating from each initial state to compute the conditional transition probability: *P(next state∣current state)* resulting in a square matrix of size *n* × *n*, where *n* represents the total number of state categories, including wake, NREM, REM, and low-activity states. The transition probability was calculated independently for each neuron. Population-level transition probabilities were obtained by averaging transition matrices across neurons (mean ± SEM).

Deviation from temporal randomness was assessed using a within-neuron permutation control. For each neuron, session order was randomly permuted while preserving overall state composition across sessions. A chance-level reference transition probabilities were computed by permuting the temporal sequence of each neuron’s classification, thereby preserving per-neuron state frequencies while disrupting temporal structure. A second chance-level reference was generated by independently assigning random state labels to each neuron and session, uniformly sampled from the allowable state set. This fully randomized matrix preserves matrix dimensions but eliminates both temporal continuity and original state composition, assuming an (unrealistic) case where each neuron has the same probability of being classified in each state.

### Calcium traces integrals and integral frequency

Single-cell calcium traces were baseline corrected (baseline subtraction), and the integral of the entire trace was computed as the simple sum of all data points belonging to a specific state. The value obtained was divided by the duration of the state to obtain the ‘integral frequency’. Only states with a duration of 20 s or longer were considered to exclude unstable states.

### Network and betweenness analysis

The betweenness is a measure of the centrality of a node in a network, based on an estimation of how ‘disruptive’ to the network the removal of that specific node would be. To compute it, we first make a correlation-based network analysis between each calcium trace. As a pre-processing step for the correlation analysis, white noise, of the same amplitude as the estimated noise of the calcium traces, was summed to the raw calcium traces, to minimize the influence of noise correlations. State-specific traces were built by isolating all the stable states (longer than 20 s) of the same type, and gluing them together into three single traces, one for each state, ending up with 3 × *number of cells* traces. These state-specific traces were then correlated with each other over a sliding time window of length = 60 frames (6 s). Shuffled traces were used to compute a threshold for minimal significant correlation, for each state: this threshold was then used to weed out any non-significant correlation, setting it to zero. The remaining significant correlations were used to construct the upper triangular adjacency matrix for the undirected weighted graph, built via the “graph” Matlab function. Autoconnections, inactive cells, and cells that were isolated were removed from the graph and not considered in the rest of the analysis. Then, we computed the undirected betweenness centrality *C* of a node *u* as (2):

2 $$C(u)=\mathop{\sum }\nolimits_{{\mathrm{s}},{\mathrm{t}}\ne {\mathrm{u}}}(n_{st}(u)/N_{\mathrm{st}})$$

where *n*_*st*_ is the number of shortest paths from *s* to *t* that pass through node *u*, and *N*_*st*_ is the number of shortest paths from *s* to *t*.

### Pharmacology treatment and recording

Diazepam solution (DZP, Valium, 5 mg/ml) was prepared by diluting it in NaCl to a concentration of 2.5 mg/ml. To mitigate the stress associated with intraperitoneal injections, mice underwent a three-day acclimatization prior to the commencement of the experiment, during which they received 0.1 ml of NaCl intraperitoneally each day. This pre-treatment aimed to familiarize the mice with the handling and injection procedures. To explore the effects of DZP on sleep phases in mice, either 0.1 ml NaCl or DZP at the dose of 5 mg/kg, was administered 10 min before the onset of the light phase (ZT0, 8 a.m.) for three consecutive days. EEG and EMG recordings were then continuously taken for 12 h following the initial administrations to monitor changes in sleep patterns. Additionally, calcium imaging targeting POA-Vgat neurons was performed for 30 min at 1-, 4-, and 8-h post-administration each day to evaluate the neuronal activity response to the treatments.

Following this phase, the animals were left undisturbed and handled minimally for at least 3 days to ensure complete metabolism of DZP before beginning treatments in the active phase. Subsequently, a single injection of either 0.1 ml NaCl or DZP (5 mg/kg) was administered 10 min before the start of the night phase (ZT12, 8 p.m.). EEG and EMG were again recorded for 12 h to assess the sleep patterns post-treatment. Concurrently, calcium imaging was conducted 1-h post-treatment for approximately 30 min to monitor the neuronal activities.

For LH-Vgat and LH-Hcrt/OX mice, following injection habituation, a single dose of saline or DZP was administered at ZT0 and ZT12, with a washout period of at least 3 days between time points. Recording procedures were identical to those used in POA-Vgat mice.

### Immunohistochemistry

To assess viral vector expression and confirm accurate anatomical targeting of brain regions, brain slices were imaged after the conclusion of all experiments. Mice were deeply anaesthetized using an intraperitoneal injection of pentobarbital (250 mg/kg, Streuli Pharma). Once fully anesthetized, transcardial perfusion was performed with 5–10 ml of cold saline (0.9% NaCl), followed by 20–25 ml of 4% formaldehyde (Grogg Chemie) to fix the tissues. After perfusion, the brains were removed and stored overnight in formaldehyde at 4 °C for complete fixation. The following day, the brains were transferred to a phosphate-buffered saline (PBS) solution containing 30% sucrose and kept there until they sank, ensuring full cryoprotection before sectioning.

The brains were sectioned into 40 µm slices using a cryostat (Hyrax C 25, Zeiss) and arranged in triplicate sets of 1:3 series, which were collected in PBS. To verify the co-expression of GCaMP6 with MCH or Hcrt/OX, sections were washed in PBST (PBS with 0.1% Triton X-100, Sigma-Aldrich) for 5 × 5 min and incubated for 45 min at room temperature in a blocking solution containing PBST and 4% bovine serum albumin (Sigma-Aldrich). The sections were then incubated with anti-pMCH (goat, 1:500, sc-14509, Santa Cruz Biotechnology) or anti-Orexin A (rabbit, 1:1000, H-003-30, Phoenix Pharmaceuticals) for 24–48 h.

After incubation, the sections were washed again for 5 × 5 min and then incubated for 2 h at room temperature with AlexaFluor555-conjugated secondary antibody (1:1000 dilution, Invitrogen, A21432 for MCH and Hcrt/OX staining). The sections were then washed for 3 × 10 min and mounted on glass slides. Cover slips were applied using Fluoromount (F4680, Sigma-Aldrich). The GCaMP6s fluorescence was easily detected, and no additional staining was necessary.

Slides were imaged using a Nikon-PLAN Fluor 10×/0.3NA objective on a Nikon Eclipse Ti-E fluorescence microscope controlled by Nikon NIS software. Excitation was achieved with a Solar LED lamp using Cy3 (mCherry) and FITC (eGFP) filters. For display purposes, image brightness and contrast were moderately adjusted in Fiji (ImageJ), and figures were compiled using Adobe Illustrator 2020.

### Statistics and reproducibility

All data are presented as means ± standard error of the mean (SEM). Unless otherwise stated, statistical tests were two-sided and an alpha level of 0.05 was used. Statistical analyses were performed in MATLAB (MathWorks, R2019b) and GraphPad Prism 10.4.2.

For comparisons between two groups, two-tailed t-tests were used when data met assumptions for parametric testing. For repeated-measures designs with more than two conditions, one-way repeated-measures ANOVA with Tukey’s post hoc test or two-way repeated-measures ANOVA with Sidak’s correction was applied, as appropriate. For datasets with large sample sizes (e.g., cell-level measurements), normality was assessed and the choice of statistical test was based on the observed distribution. For datasets with small sample sizes (e.g., animal-level measurements), data were assumed to be approximately normally distributed.

No formal power analysis was performed; sample sizes were chosen to be consistent with those commonly used in similar studies. Experimenters were not blinded to experimental conditions during data acquisition or analysis. Significant results are shown in the figures, and sample sizes are provided in the corresponding figure legends. Detailed statistical information and the underlying source data are provided in the Source Data file.

Representative images in Fig. 1a, h, m, r and Fig. 4a were obtained from in vivo miniscope recordings or fluorescence microscopy of brain slices from 6 animals per mouse line, with similar results observed across animals and recording sessions.

### Reporting summary

Further information on research design is available in the Nature Portfolio Reporting Summary linked to this article.

## Supplementary information

Supplementary Information

Peer Review file

Description of Additional Supplementary Files

Supplementary Movie 1

Supplementary Movie 2

Reporting Summary

## Source data

Source Data

## Data Availability

Source data are provided with this paper. The processed datasets used for neuron activity longitudinal tracking have been deposited in Figshare under an open-access license CC BY 4.0. 10.6084/m9.figshare.31441240. Additional raw miniscope imaging, EEG/EMG data are available under restricted access because of their large file size. Access can be requested from the corresponding authors for academic purposes. Source data are provided with this paper.

## Code availability

All the MATLAB scripts used for data processing and statistical analysis, together with a representative sample dataset, are publicly available at the GitHub repository: https://github.com/ZENLabCode and accessible via 10.5281/zenodo.20747987.
