# Supplementary material for: Temporal drift of sleep-wake representations in hypothalamic neuronal ensembles
Source: Nat Commun. 2026 Jul 28;17:9137. doi: 10.1038/s41467-026-75792-1 (PMC13519010; doi:10.1038/s41467-026-75792-1)
Supplement: Supplementary file 1 [file 41467_2026_75792_MOESM1_ESM.pdf]

Temporal drift of sleep-wake representations in hypothalamic neuronal ensembles

Yudong Yan (闫雨冬)[1,2], Niccolò Calcini[1,2,3], Thomas Rusterholz[1,2], Carolina Gutierrez Herrera[1,2] & Antoine Adamantidis[1,2]

Affiliations:

[1] Zentrum für Experimentelle Neurologie, Department of Neurology, Inselspital University Hospital Bern, University of Bern, Bern, Switzerland.

[2] Department of Biomedical Research, University of Bern, Bern, Switzerland.

[3] Present affiliation: Department of Neuroscience, Yale School of Medicine, Yale University, New Haven, CT, USA.

These authors contributed equally: Yudong Yan, Niccolò Calcini.

Correspondence and requests for materials should be addressed to Carolina Gutierrez Herrera (carolina.gutierrez@unibe.ch) or Antoine Adamantidis (antoine.adamantidis@unibe.ch)

Supplementary Figures 1-11

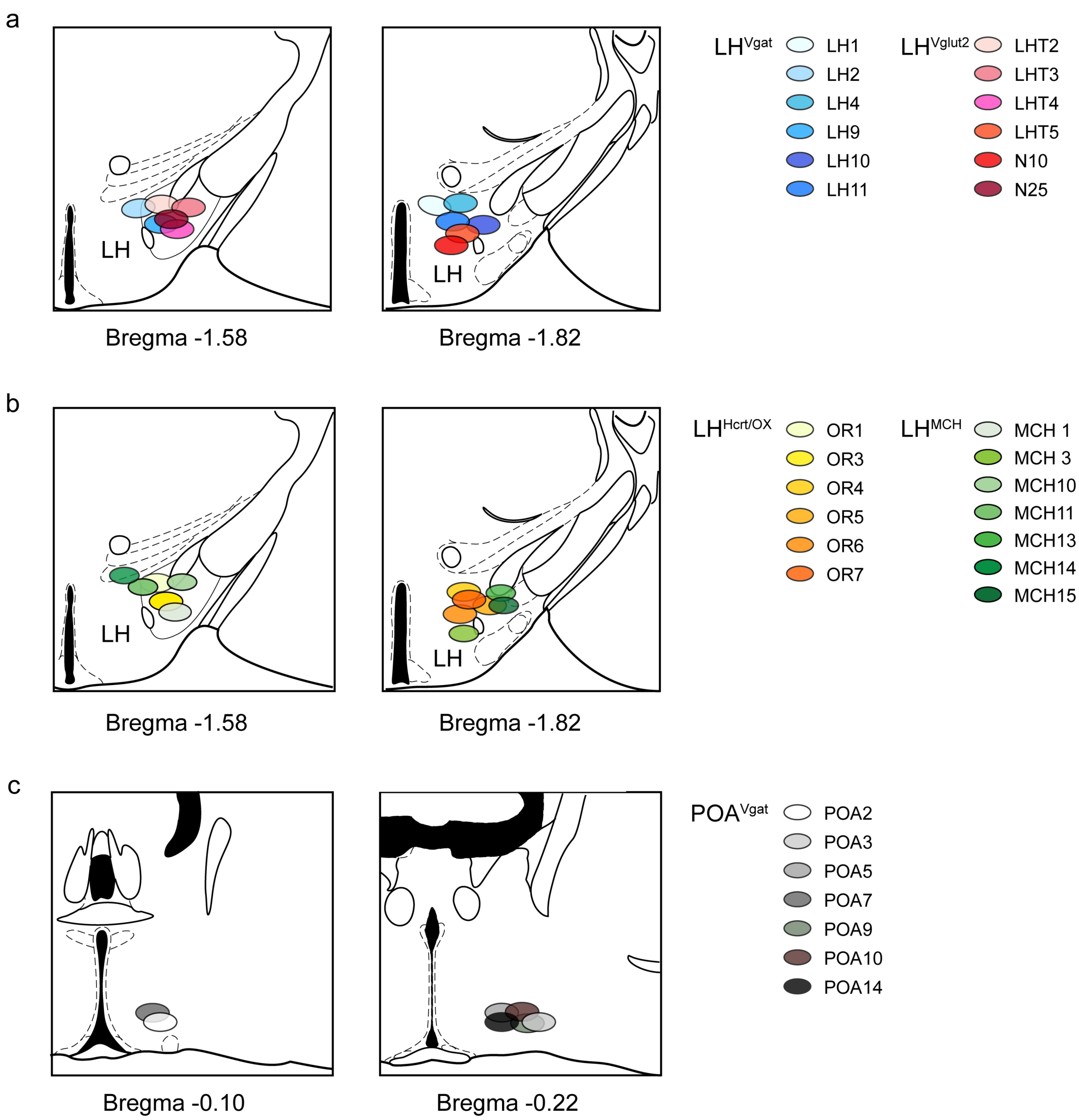

Supplementary Fig. 1: Anatomical verification of GRIN lens implantation. a-b Anatomical verification of GRIN lens placement in the LH targeting LH-Vgat (m = 6), LH-Vglut2 (m = 6), LH-Hcrt/OX (m = 6), and LH-MCH (m = 7). The central placement of the lens spans from anterior-posterior -1.58 mm to -1.82 mm, referenced from Bregma. **c** Anatomical verification of GRIN lens placement in the POA targeting POA-Vgat (m = 7) neurons. The central placement of the lens spans from anterior-posterior -0.1 mm to -0.22 mm, referenced from Bregma.

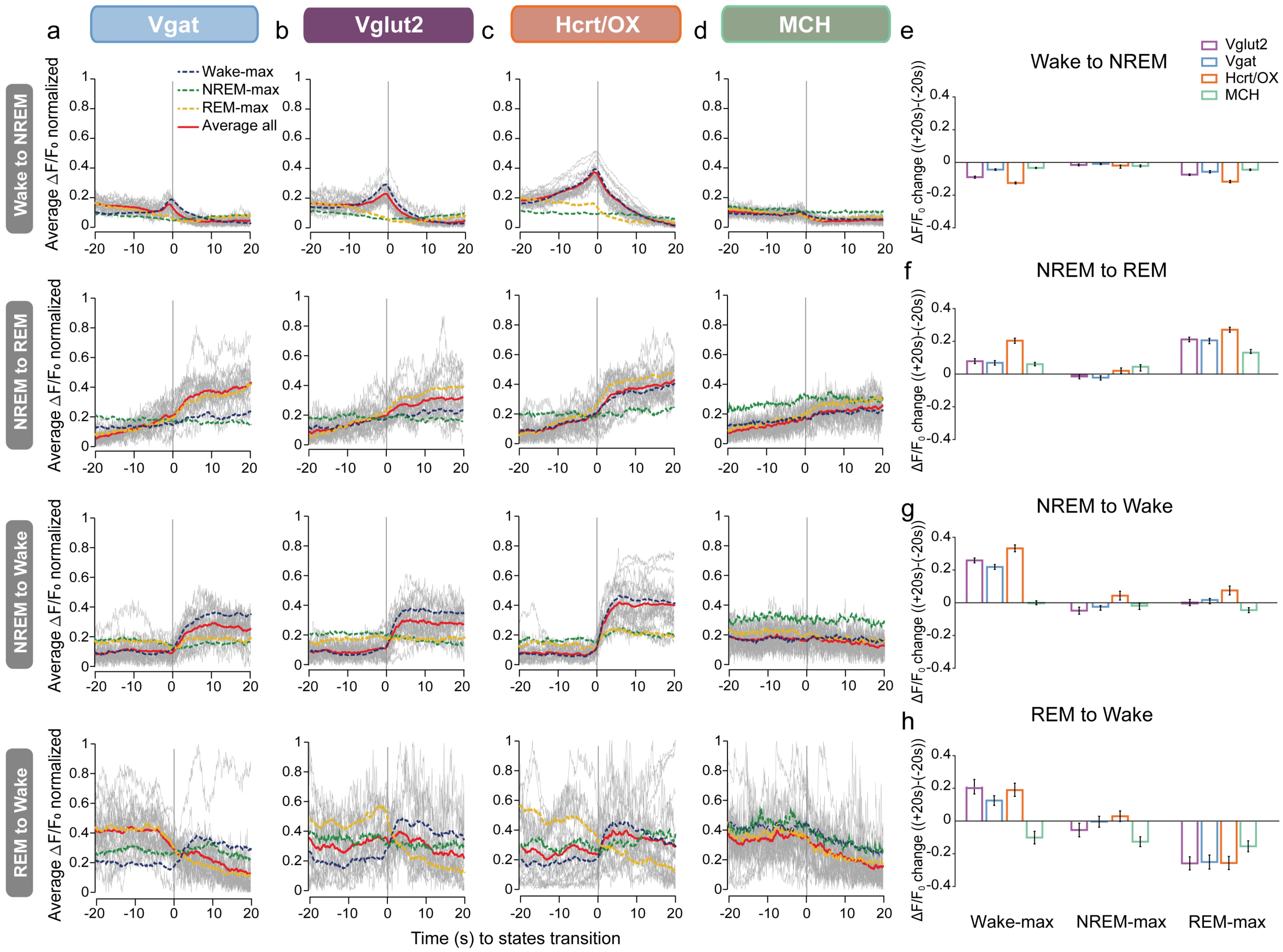

Supplementary Fig. 2: Average calcium transients at sleep-wake state transitions based on neuronal selectivity. a-d Average $\Delta F/F_0$ calcium traces aligned to state transitions for **(a)** LH-Vgat (n = 509 neurons, m = 6 mice), **(b)** LH-Vglut2 (n = 257 neurons, m = 6 mice), **(c)** LH-Hcrt/OX (n = 425 neurons, m = 6 mice), and **(d)** LH-MCH (n = 28 neurons, m = 7 mice) neurons, respectively. Neurons were categorized by state selectivity (wake, NREM, REM) based on their maximal Ca^{2+} event frequency during each recording session and the average $\Delta F/F_0$ within each selectivity cluster was calculated across sessions. Traces span ±20 seconds relative to each transition point. Gray traces represent the average $\Delta F/F_0$ in each recording session (21 sessions in total); red lines indicate the grand average across all sessions, both irrespective of neuron selectivity. **e-h** Quantification (mean ± SEM) of $\Delta F/F_0$ changes from -20 to +20 seconds during **(e)** wake-to-NREM, **(f)** NREM-to-REM, **(g)** NREM-to-wake and **(h)** REM-to-wake transitions. Analyses are performed across neuron clusters with maximal event frequency during wake (wake-max), NREM (NREM-max), and REM (REM-max) sleep for each studied LH subpopulation.

Supplementary Figures

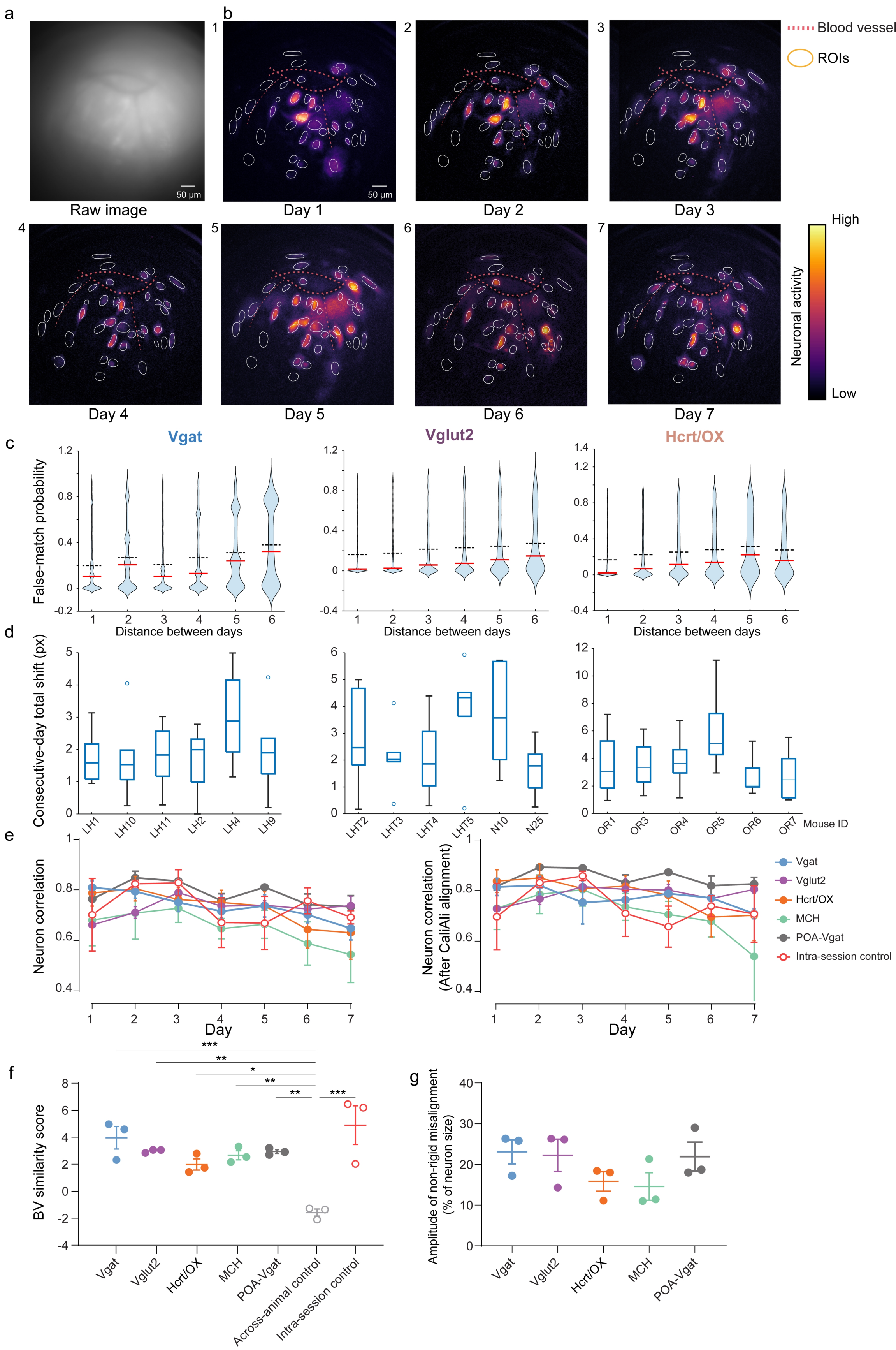

Supplementary Fig. 3: Example ROIs and validation of longitudinal neuron tracking.

a Raw in vivo field of view from an example LH-Vglut2 mouse (N25) recorded with miniscope. Fluorescently labeled neurons and background blood vessels are visible (scale bar: 50 µm). **b** Corresponding field of view (FOV) across 7 recording days after alignment using vascular landmarks. Images show projection maps of neuronal activity from morning recording sessions, scale bar: 50 µm. Color scale represents low to high activity. The same manually defined ROI set was used across sessions to track individual neurons longitudinally. **c-d** Validation of longitudinal ROI stability using the manually defined ROI set. **c** Shuffle-based false-match probability between recording days for LH-Vgat (n = 509 ROIs), LH-Vglut2 (n = 257 ROIs), and LH-Hcrt/OX (n = 425 ROIs) datasets. Red lines and black dashed lines indicate the median and mean, respectively. False-match probability represents the fraction of shuffled ROI comparisons producing equal or greater similarity than the true ROI match. **d** Consecutive-day total image displacement for each Vgat, Vglut2 and Hcrt/OX mouse (as shown in **Supplementary Fig. 1**). Box plots show the median, interquartile range and range; points indicate outliers. Displacement values represent the total rigid translation magnitude in pixels between consecutive recording days. **e-g** Independent neuronal and FOV stability validation using CaliAli (n = 3 mice in each group). **e** Correlation of CaliAli-extracted neuronal components across 7-day recordings before and after inter-session alignment. These neuronal components were defined independently by CaliAli for validation purposes and were not the manually defined ROIs used for downstream $\Delta F/F_0$ extraction. **f** Blood-vessel similarity score used to assess FOV consistency across recording days. Note that animals without clear BV structure have lower BV scores. **g** Estimated amplitude of non-rigid FOV misalignment, expressed relative to neuronal size. Statistical analyses and source data are listed in the Source Data file.

Supplementary Figures

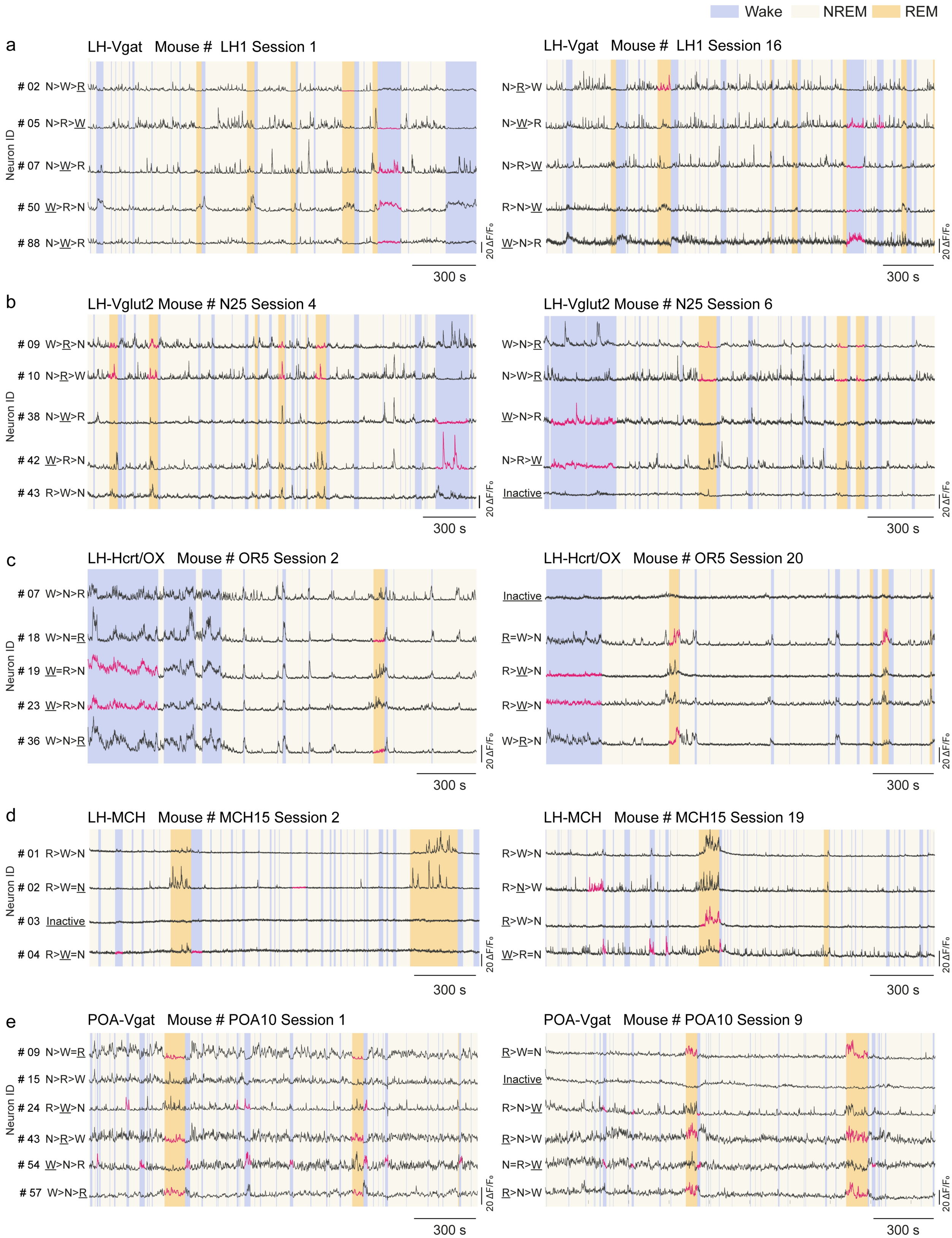

Supplementary Fig. 4: Example neuronal calcium transients across sleep-wake cycles within and across recording sessions. Representative $\Delta F/F_0$ activity profiles across sleep-wake states from example neurons in **a** LH-Vgat, **b** LH-Vglut2, **c** LH-Hcrt/OX, **d** LH-MCH, and **e** POA-Vgat mice. Neuronal calcium activity is shown across multiple sleep stages and sessions, with activity ranking determined independently for each session. The underlined segments highlight the sleep-wake state during which each neuron changed its activity profile in a later session, and the corresponding calcium transients during this state are marked in red.

Supplementary Figures

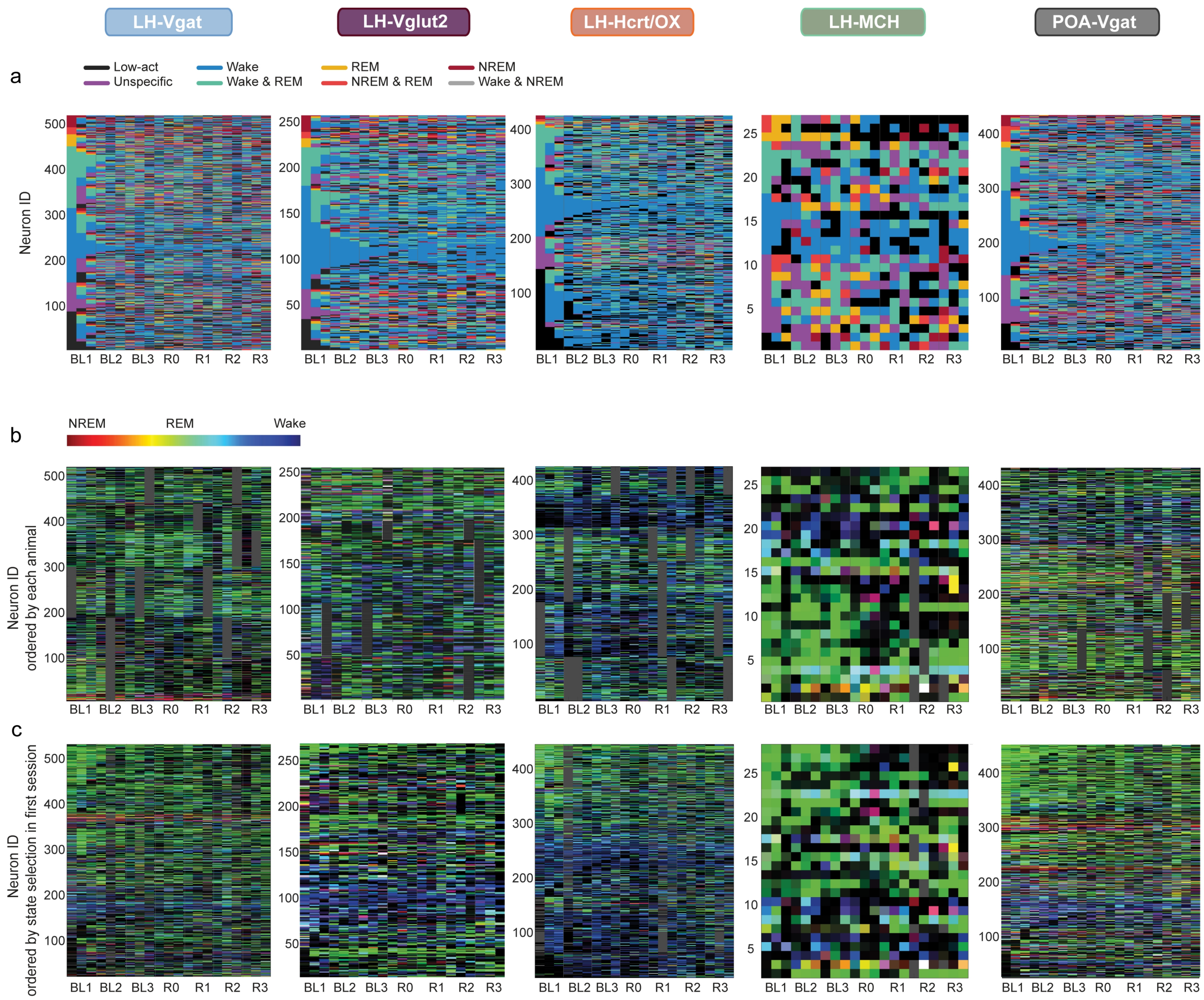

Supplementary Fig. 5: Evolution of single-neuron sleep-wake state specificity across 7 days, shown as multi-state selectivity heatmaps and RGB color-coded maps. a-c Heatmaps showing sleep-state specificity of individual neurons over 7 days (21 recording sessions) in LH-Vgat (n = 509 neurons, m = 6 mice), LH-Vglut2 (n = 257 neurons, m = 6 mice), LH-Hcrt/OX (n = 425 neurons, m = 6 mice), LH-MCH (n = 28 neurons, m = 7 mice) and POA-Vgat (n = 442 neurons, m = 6 mice), respectively. **a** Neurons were classified using a threshold-based method: labeled as Low-activity if their activity was below the 25th percentile in all states; and as selective if activity in one state was significantly higher than in the others, or showed comparable activity across multiple states. Neurons were then categorized as single-state selective (e.g., Wake, NREM, or REM), multi-state selective (e.g., Wake & REM), non-selective (unspecific) or low-activity. **b** RGB heatmaps visualizing the temporal dynamics of neuronal selectivity across sessions, without assigning fixed categories to neurons. Neurons are grouped by animal, and each is represented by a combination of red (NREM), green (REM), and blue (wake) values, scaled to their average event rate per state per session. Mixed colors reflect neuron's activity profile in a given session. Gray segments indicate sessions without REM sleep recorded. **c** RGB heatmaps with neurons ordered by their state selectivity rank, determined by the state with maximal event frequency in the first recording session (matching the order used in **Fig. 2a-d**).

Supplementary Figures

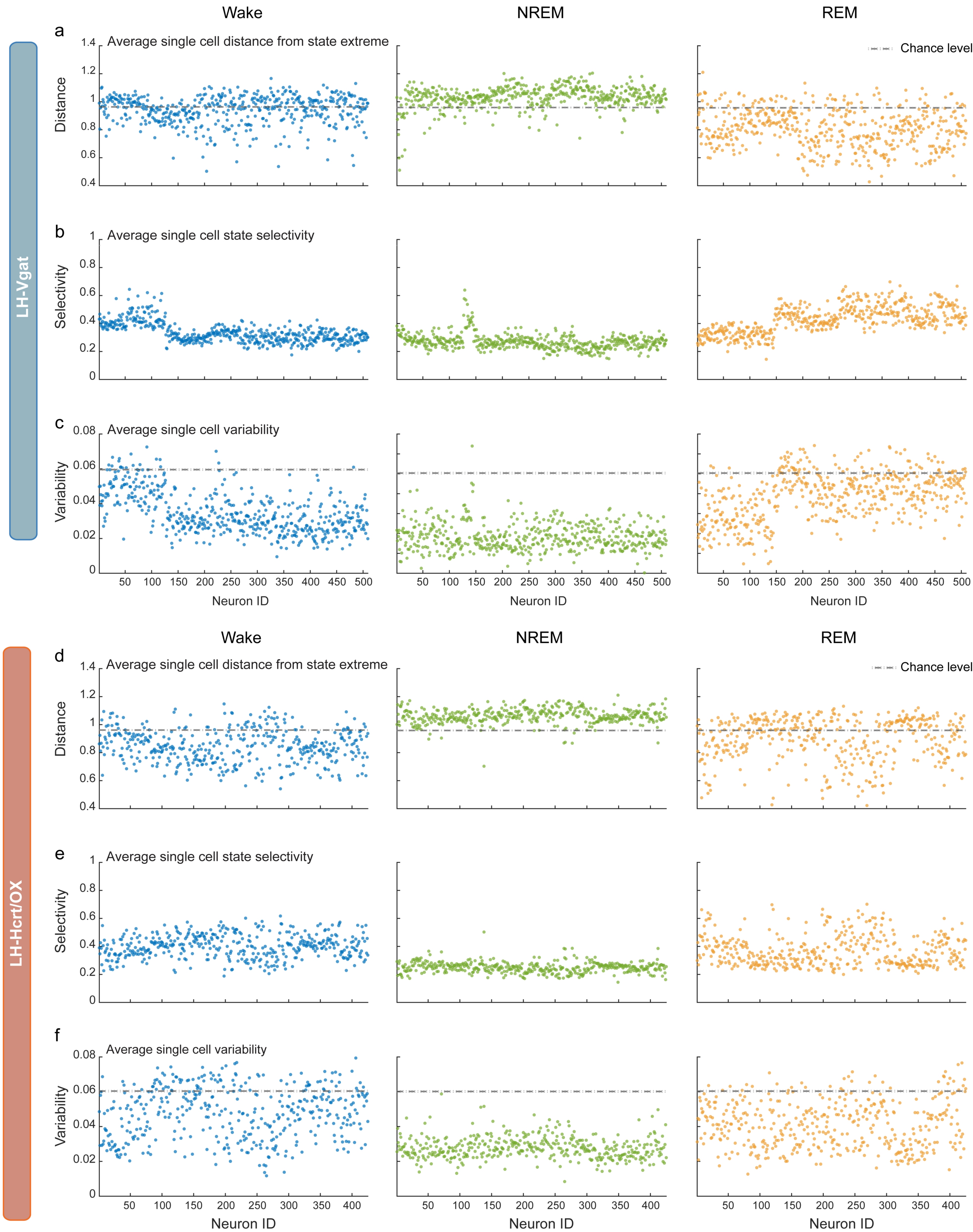

Supplementary Fig. 6: Single-neuron state selectivity and across-session variability in LH-Vgat and LH-Hcrt/OX neurons. This figure provides the quantitative analysis corresponding to Movie S1 and S2, in which each neuron's normalized activity and selectivity index across wake, NREM, and REM states was represented within a triangular state space defined by the canonical extremes. **a, d** Mean distance of individual neurons from each of the "state selectivity vertices" (used in Movie S1) across all sessions. Maximum distance = $\sqrt{2}$. **b, e** State-selectivity index of each neuron, higher values indicate stronger selectivity. **c, f** Across-session variability (SEM) of distance to each state vertex, reflecting the temporal stability of state selectivity. Dashed lines indicate mean ± SEM derived from a control in which neurons' coordinates in the state-selectivity space were randomly assigned for each session, providing a chance-level reference. LH-Vgat: n = 509 neurons from 6 mice; LH-Hcrt/OX: n = 425 neurons from 6 mice.

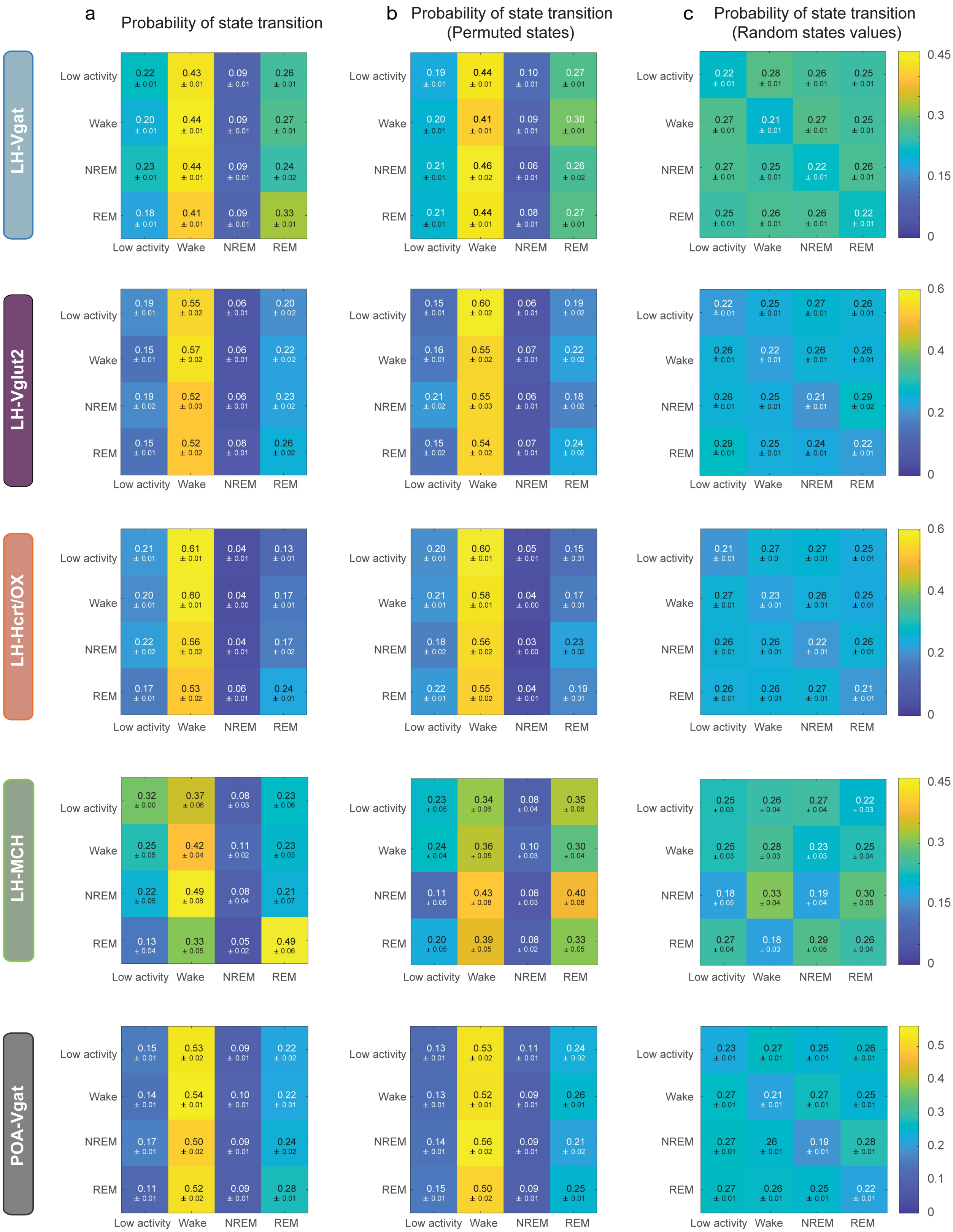
a
Probability of state transition
b
Probability of state transition
(Permuted states)
c
Probability of state transition
(Random states values)
LH-Vgat
LH-Vglut2
LH-Hcrt/OX
LH-MCH
POA-Vgat
Low activity
Wake
NREM
REM
0.45
0.3
0.15
0
0.6
0.4
0.2
0
0.6
0.4
0.2
0
0.45
0.3
0.15
0
0.5
0.4
0.3
0.2
0.1
0

Supplementary Fig. 7: Probability of state-selectivity transitions across longitudinal recordings. Heatmaps show the mean conditional probability of transitioning from one state selectivity (rows) to another state selectivity (columns) between consecutive recording sessions for LH-Vgat (n = 509 neurons, m = 6 mice), LH-Vglut2 (n = 257 neurons, m = 6 mice), LH-Hcrt/OX (n = 425 neurons, m = 6 mice), LH-MCH (n = 28 neurons, m = 7 mice) and POA-Vgat (n = 442 neurons, m = 6 mice) neurons. Values represent mean ± SEM across neurons. **a** Observed transition probabilities calculated using the original longitudinal state labels across recording sessions. **b** Transition probabilities calculated following a within-neuron permutation of session order, which preserves state composition but disrupts temporal continuity. **c** Transition probabilities derived from assigning each neuron a random state selectivity at each recording session, representing a chance-level null model.

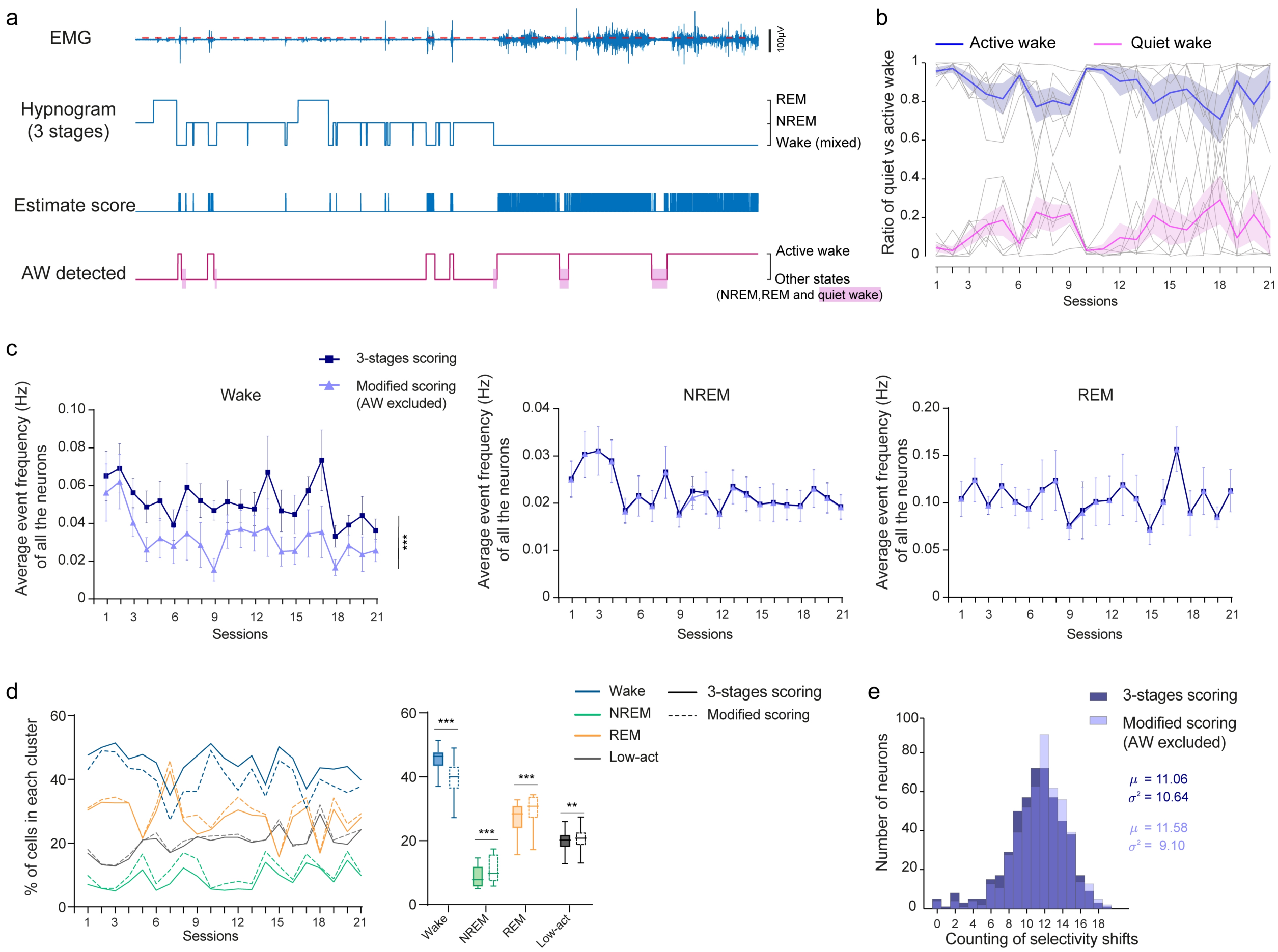

Supplementary Fig. 8: Active wake detection and comparative analysis of neuronal activity during sleep-wake cycles in LH-Vgat animals.

a Representative example of active wake (AW) detection based on EMG signals and hypnogram scoring. AW was identified using an optimized EMG threshold (red dashed line), applied only when the hypnogram indicated wakefulness. An estimated score was assigned during EMG threshold crossings within wake epochs, followed by post-processing to remove or concatenate fragmented segments. Combining the AW detection score with the hypnogram, distinct states were classified: active wake, quiet wake, NREM, and REM sleep. Subsequent analyses compared neuronal activity under conventional 3-stages scoring (wake, NREM, REM) versus modified scoring excluding active wake, to isolate the specific contribution of AW to wake-related activity. **b** Ratio (mean ± SEM) of active wake (blue) versus quiet wake (pink) across 21 recording sessions (n = 6, individual mice shown in gray). **c** Average Ca^{2+} event frequency of neurons during wakefulness, NREM and REM sleep, comparing 3-stages scoring datasets with modified scoring (AW-excluded) dataset. **d** Percentage of neurons assigned to each state-selective cluster (wake, NREM, REM, or low-act) across all 21 recording sessions, compared between the two datasets. **e** Distribution of neurons based on the number of times their state selectivity changed across 21 sessions in each dataset. μ and σ^2 indicate the mean and variance, respectively. **c-e** n = 509 neurons from 6 animals. **P < 0.01, ***P < 0.001. Statistical analyses and source data are listed in the Source Data file.

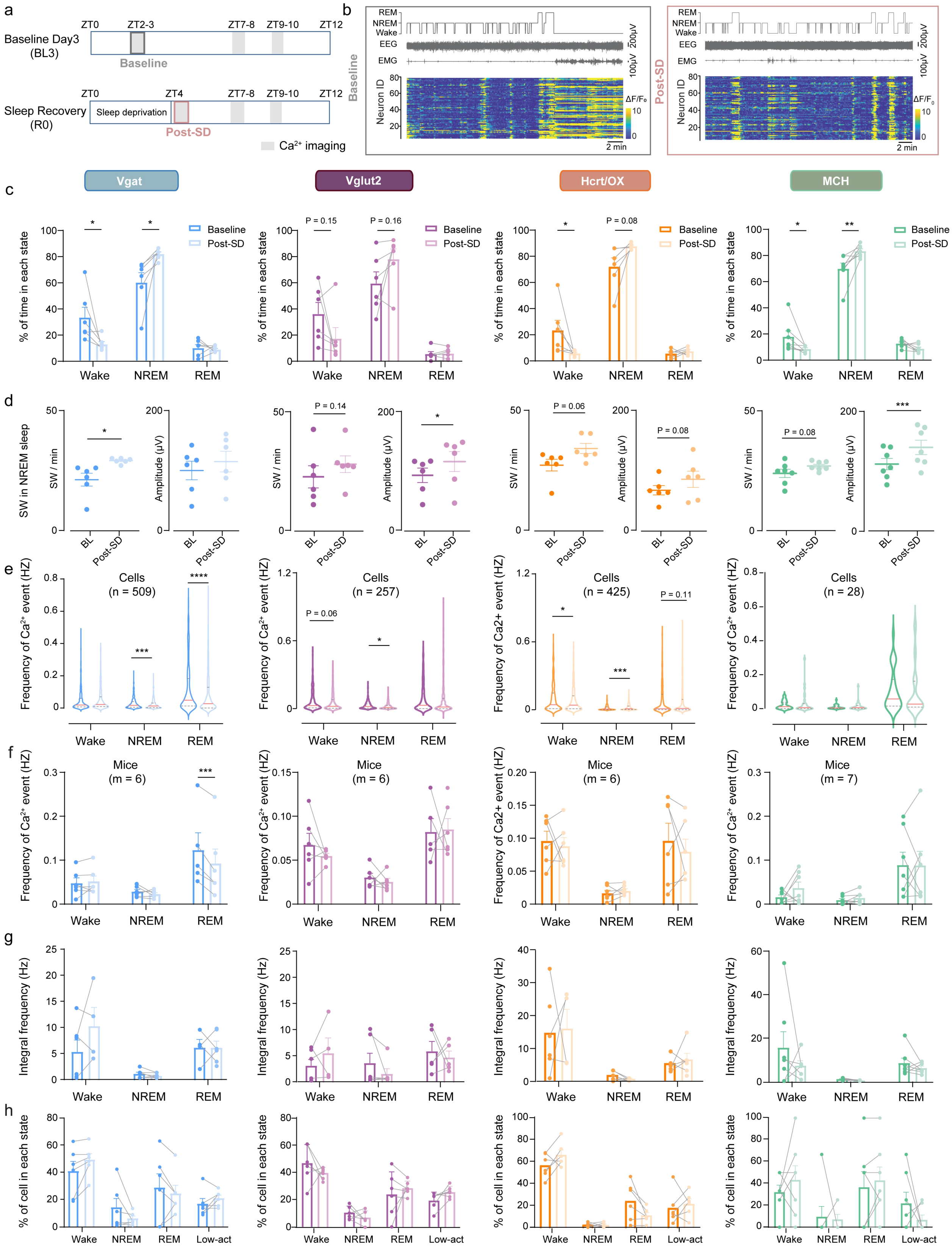

a
Baseline Day3 (BL3)
ZT0 ZT2-3 ZT7-8 ZT9-10 ZT12
Baseline
Sleep Recovery (R0)
ZT0 ZT4 ZT7-8 ZT9-10 ZT12
Sleep deprivation
Post-SD
Ca²⁺ imaging
b
Baseline
Post-SD
REM
NREM
Wake
EEG
EMG
Neuron ID
ΔF/F₀
2 min
c
Vgat
Vglut2
Hcrt/OX
MCH
% of time in each state
Baseline
Post-SD
Wake NREM REM
d
SW in NREM sleep
SW / min
Amplitude (µV)
BL Post-SD
e
Frequency of Ca²⁺ event (HZ)
Cells (n = 509)
Cells (n = 257)
Cells (n = 425)
Cells (n = 28)
f
Mice (m = 6)
Mice (m = 6)
Mice (m = 6)
Mice (m = 7)
g
Integral frequency (Hz)
h
% of cell in each state
Wake NREM REM Low-act

Supplementary Fig. 9: Sleep structure and neuronal activity in the LH after sleep deprivation. a Experimental timeline illustrating calcium imaging recordings during baseline and post-sleep deprivation (post-SD) sessions, extracted from the longitudinal protocol. **b** Representative hypnograms, EEG and EMG traces, along with color-coded neuronal activity during baseline and post-SD recordings. **c** Percentage of time spent in wake, NREM and REM sleep in the baseline and post-SD group in LH-Vgat, LH-Vglut2, LH-Hcrt/OX, and LH-MCH mice. **d** Average (mean ± SEM) frequency and peak-to-peak amplitude of slow waves (SW) during NREM sleep in baseline and post-SD conditions. **e-f** Frequency of Ca^{2+} event during wake, NREM, and REM sleep across baseline and post-SD sessions, averaged per total cells (**e**) and per animal(**f**). **g** Integrated Ca^{2+} event frequency during wakefulness, NREM and REM sleep in baseline and post-SD sessions, averaged per animal. **h** Proportion of neurons that are low-activity or state-selective during wake, NREM, and REM sleep, compared between baseline and post-SD. In bar plots, data are presented as mean ± SEM; dots represent individual animals, and connecting lines indicate paired measurements before and after sleep deprivation. In violin plots, data are presented as median (red solid line) with 25th and 75th percentiles (grey dashed line). *P < 0.05, **P < 0.01, ***P < 0.001. For **c, d, f, g** and **h**, LH-Vgat: n = 6 mice; LH-Vglut2: n = 6 mice; LH-Hcrt/OX: n = 6 mice; LH-MCH: n = 7 mice. For **e**, LH-Vgat: n = 509 neurons; LH-Vglut2: n = 257 neurons; LH-Hcrt/OX: n = 425 neurons; LH-MCH: n = 28 neurons. Statistical analyses and source data are listed in the Source Data file.

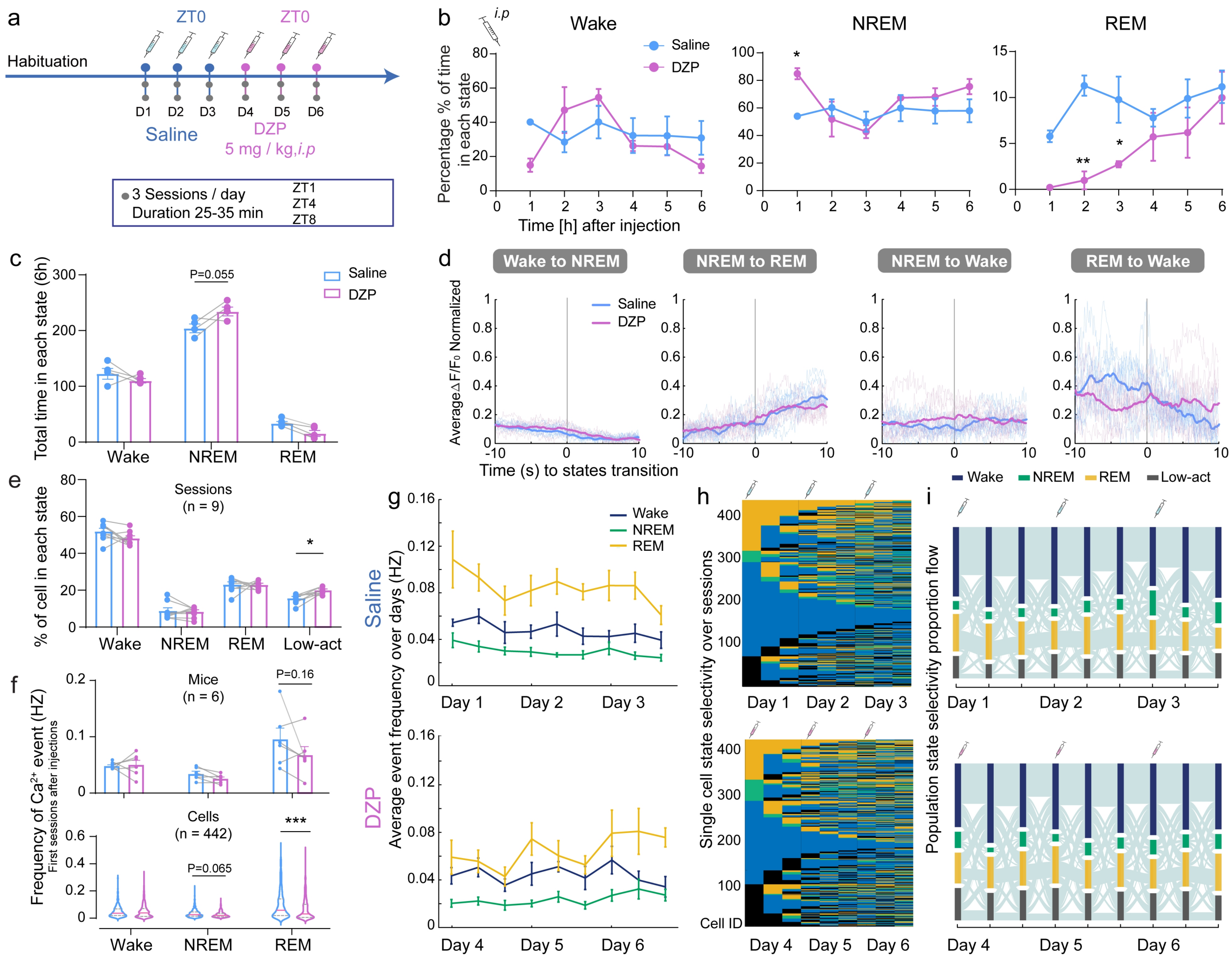

Supplementary Fig. 10: Consecutive DZP injections at ZT0 increase NREM sleep without altering POA-Vgat neuronal activity patterns during NREM sleep. a Schematic of saline and DZP injection protocol and recording timeline. **b** Time course of wake, NREM, and REM sleep percentages over 6 hours following saline or DZP treatment. **c** Cumulative duration of wake, NREM, and REM sleep within 6 hours post-treatment. For **b** and **c**, n = 4 mice; repeated measurements in both conditions. **d** Average calcium traces at state transitions after each treatment (-10 to +10 seconds). **e** Percentage of neurons classified as low-activity or preferentially active during wake, NREM, or REM sleep under each treatment, averaged per session. For **d** and **e**, n = 9 sessions per condition. **f** Ca^{2+} event frequency (Hz) across sleep-wake states, averaged per animal (n = 6) and per neuron (n = 442). **g** Mean ± SEM calcium event frequency (Hz) of POA-Vgat neurons during wake, NREM, and REM sleep in each recording under saline or DZP treatment. **h** Heatmap showing sleep-state specificity of individual POA-Vgat neurons over 3 days in saline and DZP conditions. **i** River plot illustrating the evolution of neuronal state selectivity across days following treatment in both groups. In bar plots, data are presented as mean ± SEM; dots represent individual animals, and connecting lines indicate paired measurements before and after treatment. In violin plots, data are presented as median (red solid line) with 25th and 75th percentiles (grey dashed line). For **g-i**, n = 442 neurons from 6 mice. *P < 0.05, **P < 0.01, ***P < 0.001. Statistical analyses and source data are listed in the Source Data file.

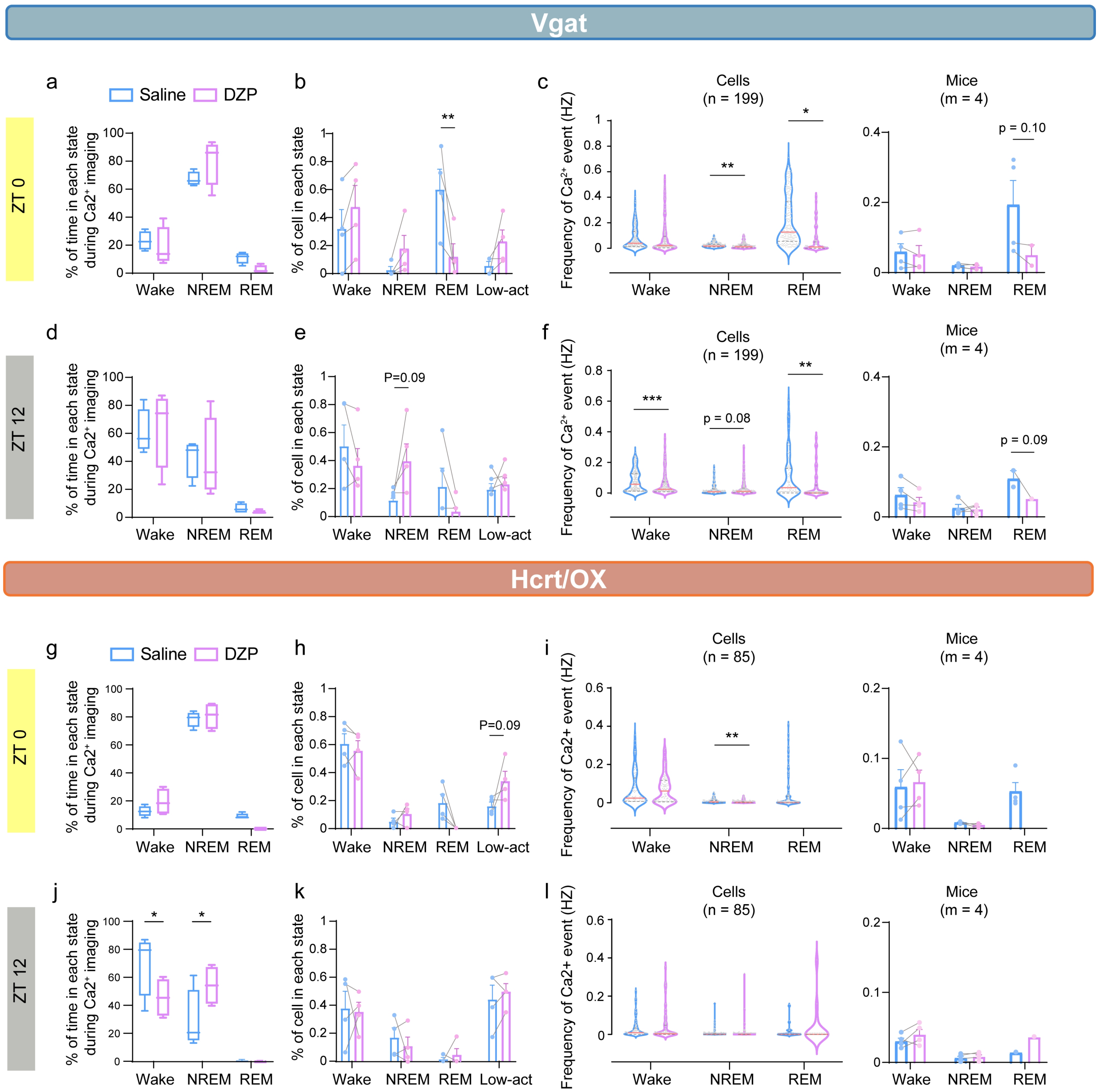

Vgat
a
Saline
DZP
ZT 0
% of time in each state during Ca2+ imaging
Wake
NREM
REM
b
**
% of cell in each state
Low-act
c
Cells (n = 199)
Mice (m = 4)
Frequency of Ca2+ event (HZ)
**
*
p = 0.10
d
ZT 12
e
P=0.09
f
***
p = 0.08
**
p = 0.09
Hcrt/OX
g
h
P=0.09
i
Cells (n = 85)
Mice (m = 4)
Frequency of Ca2+ event (HZ)
**
j
*
*
k
l

Supplementary Fig. 11: Effects of DZP on neuronal activity at ZT0 and ZT12 in LH-Vgat and LH-Hcrt/OX mice. LH-Vgat mice: **a, d** Percentage of time spent in wake, NREM, and REM sleep during calcium imaging following saline or DZP injection at ZT0 (**a**) and ZT12 (**d**). n = 4 animals, repeated measurement in all conditions. **b, e** Proportion of cells classified as wake-, NREM-, REM-selective, or low-activity under saline and DZP conditions at ZT0 (**b**) and ZT12 (**e**). **c, f** Ca^{2+} event frequency (Hz) during wake, NREM, and REM sleep under saline and DZP treatment at ZT0 (**c**) and ZT12 (**f**), averaged per neuron and per animal. Hcrt/OX mice: **g, j** Percentage of time spent in wake, NREM, and REM sleep during calcium imaging following saline or DZP injection at ZT0 (**g**) and ZT12 (**j**). n = 4 animals, repeated measurements in all conditions. **h, k** Proportion of cells classified as wake-, NREM-, REM-selective, or low-activity under saline and DZP conditions at ZT0 (**h**) and ZT12 (**k**). **i, l** Ca^{2+} event frequency (Hz) during wake, NREM, and REM sleep under saline and DZP treatment at ZT0 (**i**) and ZT12 (**l**), averaged per neuron and per animal. In bar plots, data are presented as mean ± SEM; dots represent individual animals, and connecting lines indicate paired measurements before and after treatment. In violin plots, data are presented as median (red solid line) with 25th and 75th percentiles (grey dashed line). LH-Vgat: n = 199 cells from 4 animals; LH-Hcrt/OX: n = 85 neurons from 4 animals. *P < 0.05, **P < 0.01, ***P < 0.001. Statistical analyses and source data are listed in the Source Data file.
