# Supplementary material for: Temporal drift of sleep-wake representations in hypothalamic neuronal ensembles
Source: Nat Commun. 2026 Jul 28;17:9137. doi: 10.1038/s41467-026-75792-1 (PMC13519010; doi:10.1038/s41467-026-75792-1)
Supplement: Supplementary file 2 [file 41467_2026_75792_MOESM2_ESM.pdf]

nature portfolio

Peer Review File

Temporal drift of sleep-wake representations in hypothalamic neuronal ensembles

Corresponding Author: Dr Antoine Adamantidis

This file contains all reviewer reports in order by version, followed by all author rebuttals in order by version.

Version 0:

Reviewer comments:

Reviewer #1

(Remarks to the Author)
I liked this paper. I think the results are interesting and thought-provoking. There is a risk at this stage that the results are overinterpreted. This is clearly an original and potentially important set of observations. But the authors have been quite confident in asserting: "temporal drift ", without adding any nuance. There are several interesting features about these results not discussed by the authors.

GCaMP. I feel everyone in the neuroscience field (including my own group) is building a potential "house of cards", which could collapse, with the assumption that calcium activity as detected by GCaMP always reports accurate spiking of cells. First, just because there is elevated calcium does not mean that neurons will be firing – there may be some second messenger modulation going on, but not firing of cells. Second, looking at this from the other way round, what is the sensitivity threshold for the elevated calcium signal to correspond to spiking? For example, if a sleep-inducing cell in the preoptic area is only firing at e.g. 0.5 Hz, this may be perfectly adequate to induce NREM sleep, but not be picked up by a GCaMP reporter, and the cell would score as negative? This work by Yan et al would be more convincing by looking at real firing rates. Without the advent of superior genetic probes that actually report action potentials, I'm not sure how the authors can do that though. Neuropixel probes? At least some caveats should be added in the Discussion.

Another discussion point: We don't know how many neurons are needed to induce sleep. Although the patterns of the cells shift, maybe it is the smaller number of stable ones that are important for conferring sleep-wake etc? For example, how many MCH cells are actually required to contribute to REM?

Diazepam: I was puzzled by the diazepam results and their importance for what the authors are claiming, that in particular "Diazepam significantly altered the encoding of sleep" (line 251). I don't agree with this statement from the evidence presented. Diazepam will act on many types of neuron in the hypothalamus to enhance GABA's action, increasing chloride flow into the cells via GABA-A receptors. (GABA-A receptors with the critical gamma2 subunits needed for diazepam sensitivity being fairly universally expressed in the brain). Thus, many of the neurons targeted by diazepam in the hypothalamus will not be just sleep-promoting cells. So, the fact that extra cells that were previously inactive or wake-active are now "recruited" does not mean that the sleep-regulatory circuitry is expanded. It just means that all the other kinds of cell regulating the diverse functions of the POA are influenced by diazepam (in addition to the sleep-promoting ones). A second point is that it is intriguing that calcium activity (number of active cells) seems to go up in the POA following diazepam. This is quite surprising, and not discussed. This presumably happens by dis-inhibition of inhibitory cells upstream of the ones that are having an increased calcium activity? After all, some cells have to be inhibited by diazepam, and we would expect to see overall less activity in the POA area? Finally, what happens to the LH activity with diazepam? (it seems likely that diazepam might in part induce sleep by, for example, inhibiting the LH orexin cells?)

Minor point:
Line 48: "Inhibitory neurons in the preoptic area are predominantly active during NREM, and to a lesser extent REM". Ref. 24 (Miracca et al) is cited as showing this. In fact, data in that paper show that all types of neurons in the lateral preoptic hypothalamus, including vgat ones, are more active in REM (e.g. see the vgat neural activity in Fig. 1F of that paper). This is in agreement with Yan et al's current findings.

"Relatively rapid time scale of shifts in state-selective activity patterns suggested that these changes are not sole input-

driven … and may include specific gene expression, protein translation…". Gene expression and translation are relatively slow and not relatively rapid? Maybe qualify, reword, make more precise what is meant by "rapid"?

Happy to sign as: William Wisden

(Remarks on code availability)

Reviewer #2

(Remarks to the Author)
Yan et al. investigated the longitudinal stability of single-cell sleep-state selectivity in genetically defined neuronal populations in the lateral hypothalamus (LH). Using single-cell calcium imaging in freely moving sleeping mice, the authors observed that LH sub-populations show different sleep-state activities and that the selectivity of single neurons shifts over time. However, at the population level, the distribution of state-active neurons remains stable and is robust despite acute disruptions. These findings are consistent in both the LH and POA. Additionally, diazepam was found to increase the activity and NREM sleep selectivity of POA-Vgat neurons.
Major comments:
1. The first major concern regarding the definition of state selectivity arises from the potential conflation of neuronal activity with behavioral intensity within wake.
The method of defining neuronal state specificity based on the highest event rate within a recording session is potentially confounded by the heterogeneity of behavioral substates. For instance, a session with a high proportion of active wakefulness (e.g., exploration, grooming) is likely to have a higher overall wake event frequency in many neurons compared to a session dominated by quiet wakefulness.
Consequently, a neuron that is genuinely selective for "high-arousal" behaviors might be misclassified as "wake-specific" in one session and not in another, purely due to the difference in the animal's behavioral composition during those recordings. This ambiguity directly impacts the interpretation of both "stable" and "switching" neurons reported in the study.
To rule out this alternative explanation, it is essential to perform control analyses. For example, the authors could:
1. Subcategorize wake epochs into active and quiet wake to check if the putative "wake-active" neurons are specifically tuned to one substate.
2. Examine the correlation between behavioral intensity metrics (e.g., velocity during wake) and neuronal event rates within each state and across sessions.
Ensuring that the observed shifts in selectivity are not merely artifacts of varying behavioral dynamics would greatly strengthen the central conclusion about the intrinsic dynamics of hypothalamic populations.

2. Assigning a single state label to each neuron may not fully capture the complexity of neuronal activity across sleep-wake cycles.
The study defines state specificity primarily based on which sleep-wake state has the highest frequency of calcium events. However, a significant question arises: for neurons that exhibit comparable levels of activity in two behavioral states (e.g., wake and REM), is it biologically meaningful or methodologically sound to assign a single, discrete state label based solely on a marginal difference in event rate?
Furthermore, the transition of a neuron's state preference is likely a gradational process rather than an instantaneous switch. The current analytical approach, which assigns a fixed selectivity to each neuron within a recording session, may not adequately capture this dynamism. It would be crucial to consider whether neurons active in multiple states could represent a population that is in the process of transitioning their functional specificity. A more granular analysis—like the RGB map in Extended Data Fig.4 could significantly strengthen the interpretation of the data and provide deeper insights into the dynamic nature of neurons, as posited by the authors' conclusion.

3. To more compellingly demonstrate the activity patterns of neurons, it would be helpful to include representative raw calcium trace examples in each brain states.
For example, the finding that a subset of orexin neurons exhibits activity during NREM and REM sleep is a significant departure from the established model of their exclusive wake-promoting function. Raw trace data would provide the most direct and convincing evidence for this claim:
1. Raw trace of orexin neurons have maximal Ca^{2+} event frequency during each of the three states (Wake, NREM, REM) within a recording session.
2. Examples of a single orexin neuron undergoing a switch in its state preference (e.g., from wake-active to REM-active) across different sessions.

4. The conclusion that sleep deprivation has minimal impact on population-level state selectivity requires further validation with a more robust paradigm.
The authors conclude that acute sleep deprivation (SD) does not significantly alter the overall distribution of state-specific neural populations, a finding that is intriguing. However, as shown in Extended Data Fig.5, the 4-hour SD protocol employed —while sufficient to induce homeostatic sleep pressure—resulted in relatively moderate changes in subsequent sleep architecture and slow-wave activity (SWA). It is therefore plausible that a more prolonged or intense sleep deprivation challenge could overwhelm the proposed stability mechanism and reveal significant reorganization within the hypothalamic and preoptic circuits.
To robustly support the claim of resilience, it would be highly informative to investigate whether the stability of neuronal population distributions holds under conditions of stronger sleep pressure, for example, following 6 or 12 hours of sleep

deprivation. Demonstrating that the system remains stable even under a more severe challenge would greatly strengthen the main conclusion.

5. The characterization of the minority of neurons that appear state-stable across recordings requires deeper investigation to distinguish biological significance from statistical chance.
The study reports a subset of neurons that maintained their state-specificity across long-term imaging. A critical question arises: do these "stable" neurons possess distinct electrophysiological or molecular properties, or could their apparent stability arise simply from stochastic probability?
To robustly address this, it would be highly informative to perform a more rigorous statistical analysis. Specifically, constructing a transition probability matrix for state selectivity at the single-neuron level across sessions would be valuable. Furthermore, comparing the observed prevalence of stable neurons against a null statistical model (e.g., one based on the overall probabilities of state assignment) could determine whether their persistence exceeds what would be expected by chance alone. Such an analysis would significantly strengthen the interpretation of these neurons, either by highlighting a biologically unique subpopulation or by confirming that the observed stability is consistent with random variation.

6. The finding that diazepam (DZP) recruits more NREM sleep-active neurons in the POA should be extended to determine its generality.
The authors present a compelling pharmacological result demonstrating that diazepam increases the proportion of NREM sleep-specific neurons within the POA Vgat population. However, to establish whether this is a general mechanism of DZP action or a phenomenon specific to the POA, it is crucial to replicate this experiment in the lateral hypothalamus (LH). Given that the study extensively characterizes the dynamic nature of various genetically defined cell types in the LH (e.g., hypocretin/orexin-, MCH-, Vglut2-, and Vgat-expressing neurons), testing the effect of DZP on these populations would be highly informative.

Minor comments:
1. Regarding the calcium signal extraction methodology: The authors mention that ROIs were manually defined in Fiji/ImageJ. While this is a valid approach, numerous established, automated, and publicly available pipelines (e.g., CaImAn, CNMF-E) have been developed to perform cell detection and signal extraction in an objective and high-throughput manner. Could the authors please clarify the rationale for choosing manual ROI definition over these automated methods?
2. Regarding the calcium signal processing methods: The manuscript mentions that an exponential curve was fit to the data for bleaching correction. Please could clarify:(1) The specific mathematical function used for this exponential fit (e.g., a single- or multi-exponential function).(2) Whether this fit was performed on the entire recording session or specifically on periods of NREM sleep data.
3. Regarding the analysis of calcium activity: The study primarily utilizes the frequency of calcium events as the key metric for neuronal activity. While event frequency is informative, it does not capture the potential variability in the amplitude or duration of calcium transients, which may also carry biological significance. Have the authors considered complementary measures, such as the area under the curve (AUC or integral) of the calcium transients? Analyzing both frequency and AUC would strengthen the analysis by ensuring that the conclusions are robust to the choice of activity metric.
4. Regarding Figure 3: It would be helpful to clarify why the betweenness centrality analysis was not included for LH-MCH neurons. Was this omission due to an insufficient number of neurons for a robust network analysis?
5. An analysis of single-neuron state transition probabilities would be valuable. Quantifying these transitions after sleep deprivation and DZP administration would provide more direct evidence.
6. Please indicate the statistical significance of the state-specific changes induced by DZP in Figure 5B. Adding p-values or significance notations to the figure would be better.

(Remarks on code availability)

Reviewer #3

(Remarks to the Author)
Yan et al. used a miniature microendoscope with single-cell resolution to record various types of neural activity in the brains of mice that were freely showing sleep/wakefulness over a long period of time, demonstrating that these activity patterns can change over time. Previous studies had suggested that activity patterns remained largely unchanged across sleep/wake states. So the concept of this manuscript is interesting; however, it is unfortunate that there is insufficient evidence to support it. The main issue is that there is no clear evidence on whether the authors reliably and continuously recorded the same neurons or not. In this study, this point should be rigorously addressed and presented with different aspects, yet the current manuscript does not provide sufficient validation of this aspect. This reviewer suggests the following points to verify the importance and credibility of the contents of this manuscript.

Major comments
1) There are various ways to prove that the same neural activity is being recorded.
・Use another protein that emits constant fluorescence as a position marker (Sato et al., 2020, Cell Rep)
・Verify the technique using a system where it is already known that the same neurons remain active over time
・Use Cal-Light, scFLARE2, CaMPARI, KikGR, etc., to confirm post-hoc using optotags or fixed brains
2) The NREM activity of orexin in Figure 2A is very low. The activity of orexin neurons during NREM sleep has been shown to exhibit synchronized activity in data from the Luis deLecea lab (Li et al., Science 2022) and the Akihiro Yamanaka lab (Ito et al., PNAS 2023), so the NREM activity of 2% seems unusual. This is probably because the authors define the maximum

value of activity as "state-active," so NREM-active neurons are almost completely absorbed by wake-active neurons, which may lead to this situation. In other words, even if wake-REM active and wake-NREM active actually exist, an approach that defines and discusses only maximum activity may be a method that hinders a direct understanding of the essence of the phenomenon. When the criteria in the above definition are relaxed or changed, it seems necessary to at least confirm how these activity patterns will change.
3) The selectivity of gene expression using the short orexin/hypocretin promoter with the virus vector is relatively lower compared to the gene-modified mouse line. It is necessary to confirm whether the expression pattern changes across time using histochemistry. It is better to use a gene-modified mouse line, such as the orexin-Cre mouse, instead of a virus vector when the authors would like to claim this type of phenomenon.
4) It is also interesting to confirm whether neural activity patterns change similarly during activities other than sleep/wakefulness, such as eating and drinking. This will clarify whether the changes are limited to sleep/wakefulness or whether other functions also change simultaneously. If changes are observed in other activities as well, the significance of these changes will be further heightened.

Minor comments
1) Figure 1H, the caption indicates Vgat-Cre, this should be Vglut2-Cre.
2) The color of the legend does not match the color in Figure 2 (wake and inactive).
3) Figure 3, it is confusing to change the color of the unspecific between A-D and E-H.
4) The second and third neurons from the top of the recorded MCH neurons in Figure 1 are completely inactive. I think they might be detected as neurons in another session. I would like to see representative data that spans multiple days, rather than just data from this one session. I think that would give us a clearer picture of the actual activity pattern.
5) In Materials and methods, Vglut2-ires-Cre (Strain #:028863), : , typo.
6) In Materials and methods, 0.8 µm.s-1, and 0.1 mg.kg-1, it is better to use "/" which is used in DZP (5 mg/kg).
7) Uniform the writing of OX/Hcrt, VGlut2 neurons, and VGAT neurons throughout the manuscript.

(Remarks on code availability)

Version 1:

Reviewer comments:

Reviewer #1

(Remarks to the Author)
The authors have revised the manuscript convincingly. It will be of broad interest to those dissecting sleep–wake circuitry. The key result, that individual hypothalamic neurons show marked, state dependent changes in activity while the overall sleep–wake ensemble remains stable, is striking and unexpected. It points to a finely tuned balance between genetically anchored circuit architecture and functional flexibility in the neurons governing sleep and wakefulness, perhaps allowing the brain to preserve reliable sleep yet adapt to changing internal and external demands.

Happy to sign as William Wisden

(Remarks on code availability)

Reviewer #2

(Remarks to the Author)
The authors have adequately addressed all my concerns in the revised manuscript. I am happy to recommend acceptance.

(Remarks on code availability)

Reviewer #3

(Remarks to the Author)
This is a revised manuscript by Yan et al. The authors have responded carefully to the previous reviews, and the revised manuscript has been substantially improved. The newly added analyses, including wake substate analysis, threshold-based and category-independent state selectivity analyses, state transition probability analysis, and the diazepam experiment in the LH population, help address several concerns raised by the reviewers and make it clearer that the conclusions do not rely solely on maximal activity assignment.

However, this reviewer's main concern has only been partially resolved. The central claim of this study is that the sleep-wake state selectivity of individual neurons drifts over time based on longitudinal tracking of the same cells. This conclusion requires particularly strong evidence that identical cells were reliably registered across days.

In the revised manuscript, the authors now explain that the miniature microscope remained attached throughout the recording period, focus was fixed, the same field of view was maintained, vascular landmarks were used for alignment, and manual ROIs were defined while viewing all recording sessions. These details are helpful and improve the manuscript. Still, they are not sufficient to quantitatively establish that each ROI corresponds to the same cell across days. Vascular landmarks support field-level stability, but they do not fully exclude mismatches between nearby cells, subtle focal plane shifts, day-to-day signal fluctuations, or errors introduced by the custom ROI extraction and registration pipeline. Therefore, it remains difficult to rule out the possibility that at least part of the reported representational drift reflects technical mismatch rather than biology.

To strengthen this key point, the revised manuscript still requires more direct validation of longitudinal cell registration. In particular, visual examples and field-level alignment using vascular landmarks are helpful, but they do not yet provide a quantitative estimate of registration accuracy or false-match rate across days. At minimum, the authors should provide a more quantitative validation, for example using ROI footprint overlap, centroid displacement, matching confidence, or shuffle/mismatch-based false-match estimates. If feasible, an additional supplementary cross-check using an established longitudinal registration pipeline on a representative subset of the data would further strengthen confidence in the conclusions. A negative-control dataset or population expected to show relatively stable assignment would also be valuable. At present, it remains difficult to determine to what extent the observed remapping reflects a genuine biological phenomenon versus technical mismatch introduced during cross-session registration.

This reviewer also suggests softening the interpretation of the diazepam data. The revised text states that diazepam increased NREM-active neurons in POA-Vgat at ZT12, whereas it "did not induce a comparable effect" in LH. However, the rebuttal notes at least a similar directional, though non-significant, trend in the LH-Vgat data. Without a direct statistical comparison between POA and LH, it would be more appropriate to state that the effect was more clearly observed in POA-Vgat, rather than implying that the effect is absent in LH or specific to POA.

Overall, I find the study interesting and agree that the manuscript has improved considerably. Nevertheless, because the central conclusion depends critically on longitudinal cell registration, additional validation—or at minimum, more quantitative support for registration accuracy—remains necessary.

(Remarks on code availability)

Version 2:

Reviewer comments:

Reviewer #3

(Remarks to the Author)
I have reviewed the second revised version of the manuscript by Yan et al.
The authors have now addressed this reviewer's remaining concern.

In response, the authors have added several useful and quantitative validation analyses. These include a shuffle-based estimate of longitudinal ROI mismatch probability, quantification of consecutive-day field-of-view displacement, and an independent cross-validation using the CaliAli alignment pipeline on a representative subset of the data. The authors also clarify that their analysis used a common ROI set across recording sessions rather than independent segmentation followed by cross-session matching.

Together, these additions substantially strengthen confidence that the reported single-cell drift is unlikely to be explained simply by technical mismatch during longitudinal registration.
I also appreciate the authors' revised wording regarding the diazepam experiments. The manuscript now more appropriately states that the DZP effect was most clearly observed in POA-Vgat neurons, while acknowledging the directional but non-significant trend in LH-Vgat neurons. This is a more balanced interpretation of the data.

Overall, the revised manuscript is substantially improved. The additional analyses address the major concern raised in my previous review, and I have no further substantive concerns.

(Remarks on code availability)

Open Access This Peer Review File is licensed under a Creative Commons Attribution 4.0 International License, which permits use, sharing, adaptation, distribution and reproduction in any medium or format, as long as you give appropriate credit to the original author(s) and the source, provide a link to the Creative Commons license, and indicate if changes were made.
In cases where reviewers are anonymous, credit should be given to 'Anonymous Referee' and the source.
The images or other third party material in this Peer Review File are included in the article's Creative Commons license, unless indicated otherwise in a credit line to the material. If material is not included in the article's Creative Commons license and your intended use is not permitted by statutory regulation or exceeds the permitted use, you will need to obtain permission directly from the copyright holder.
To view a copy of this license, visit https://creativecommons.org/licenses/by/4.0/

REVIEWER COMMENTS

Reviewer #1 (Remarks to the Author):

I liked this paper. I think the results are interesting and thought-provoking. There is a risk at this stage that the results are overinterpreted. This is clearly an original and potentially important set of observations. But the authors have been quite confident in asserting: “temporal drift “, without adding any nuance. There are several interesting features about these results not discussed by the authors.

We thank the reviewer for the supportive and constructive comments. We have carefully addressed the concerns raised and have revised the manuscript to avoid overinterpretation of the findings.

1. GCaMP. I feel everyone in the neuroscience field (including my own group) is building a potential “house of cards”, which could collapse, with the assumption that calcium activity as detected by GCaMP always reports accurate spiking of cells. First, just because there is elevated calcium does not mean that neurons will be firing – there may be some second messenger modulation going on, but not firing of cells. Second, looking at this from the other way round, what is the sensitivity threshold for the elevated calcium signal to correspond to spiking? For example, if a sleep-inducing cell in the preoptic area is only firing at e.g. 0.5 Hz, this may be perfectly adequate to induce NREM sleep, but not be picked up by a GCaMP reporter, and the cell would score as negative? This work by Yan et al would be more convincing by looking at real firing rates. Without the advent of superior genetic probes that actually report action potentials, I’m not sure how the authors can do that though. Neuropixel probes? At least some caveats should be added in the Discussion.

Thank you for raising this important point. We agree that GCaMP-based calcium imaging does not provide a direct one-to-one readout of action potential firing and that calcium signals may reflect subthreshold activity, neuromodulatory influences, or intracellular signaling dynamics in addition to spiking. Furthermore, we acknowledge that low firing rates (e.g., ~0.5 Hz) or brief high-frequency bursts may not always be reliably detected or temporally resolved, depending on signal-to-noise ratio, temporal resolution, and indicator sensitivity. Accordingly, we found many brief calcium transients (new Supplementary Fig. 4) that would be consistent with low activity firing.

Electrophysiological recordings (e.g., Neuropixels) would provide complementary spike-level information. However, chronic longitudinal tracking (i.e., across weeks) of the <u>same</u> neurons at single-cell resolution would be almost impossible in comparison to optical imaging approaches, as performed in this study. While genetically encoded voltage indicators may offer improved temporal precision and a closer approximation to membrane action potential dynamics, technical limitations—particularly signal-to-noise constraints and depth limitations in deep hypothalamic structures—currently restrict the application in long-term in vivo experiments.

Importantly, our conclusions are based on relative changes in state-dependent activity

within the same neurons across sessions, rather than absolute firing rate quantification. Thus, although calcium imaging may not capture all spiking events, the longitudinal comparisons remain internally consistent and informative. These considerations have now been added to the revised Discussion section.

2. Another discussion point: We don't know how many neurons are needed to induce sleep. Although the patterns of the cells shift, maybe it is the smaller number of stable ones that are important for conferring sleep-wake etc? For example, how many MCH cells are actually required to contribute to REM?

This is indeed an interesting conceptual point. Due to technical limitations, we are not able to image or manipulate all neurons within a given LH subpopulation. With miniscope imaging, only neurons within the field of view can be tracked longitudinally, representing a relatively small fraction of the total population in the region (i.e., 600-micron diameter lens). Therefore, our study does not determine how many neurons are sufficient or necessary to induce and maintain specific vigilance states, nor does it establish a causal role for particular subpopulations.

It remains possible that a relatively small subset of stable neurons plays a more important functional role in regulating sleep–wake transitions. Consistent with this idea, our betweenness centrality analysis (Fig. 3) identified a minority of neurons with hub-like properties within the functional network.

In addition, in the probability-of-state-transition analysis (new Supplementary Fig. 7), MCH neurons showed a distinct pattern compared to other neuron types: the REM-selective cluster exhibited a higher probability of remaining in REM compared to the permuted control ($P = 0.49$ vs 0.33), suggesting relatively preserved REM-associated stability. However, because the number of recorded MCH neurons was limited, this tendency should be interpreted cautiously.

3. Diazepam: 1） I was puzzled by the diazepam results and their importance for what the authors are claiming, that in particular "Diazepam significantly altered the encoding of sleep" (line 251). I don't agree with this statement from the evidence presented. Diazepam will act on many types of neuron in the hypothalamus to enhance GABA's action, increasing chloride flow into the cells via GABA-A receptors. (GABA-A receptors with the critical gamma2 subunits needed for diazepam sensitivity being fairly universally expressed in the brain). Thus, many of the neurons targeted by diazepam in the hypothalamus will not be just sleep-promoting cells. So, the fact that extra cells that were previously inactive or wake-active are now "recruited" does not mean that the sleep-regulatory circuitry is expanded. It just means that all the other kinds of cell regulating the diverse functions of the POA are influenced by diazepam (in addition to the sleep-promoting ones).

We agree that diazepam broadly enhances GABA-A receptor–mediated inhibition and is not selective for sleep-promoting neurons. We did not intend to suggest an expansion of sleep-regulatory circuitry, yet there was a transient increase of cells active during NREM sleep. Accordingly, our conclusion refers to a shift in state-

associated activity patterns within recorded POA-Vgat neurons following DZP administration, which should not be interpreted as an expansion of sleep circuits. We have revised the manuscript to clarify that this reflects both local and global pharmacological modulation of network dynamics, rather than selective recruitment of sleep-promoting cells.

2）A second point is that it is intriguing that calcium activity (number of active cells) seems to go up in the POA following diazepam. This is quite surprising, and not discussed. This presumably happens by dis-inhibition of inhibitory cells upstream of the ones that are having an increased calcium activity? After all, some cells have to be inhibited by diazepam, and we would expect to see overall less activity in the POA area?

It is important to note that in our analysis, neurons were classified as "inactive" or "low-activity" if their event frequency fell within the lower 25th percentile across all cells and vigilance states within a session. For these cells, state selectivity could not be estimated due to insufficient activity. Therefore, the observed increase in NREM-active neurons following DZP does not reflect a global increase in absolute firing or calcium activity, but rather a redistribution of neurons across activity-defined categories relative to the session's overall activity distribution.

Moreover, we observed an increased proportion of low-activity neurons following DZP administration at ZT0 in the POA—and to a lesser extent in LH populations—whereas this effect was not evident at ZT12. This pattern suggests that DZP modulates network excitability in a region- and time-dependent manner, rather than uniformly increasing or decreasing neuronal activity.

These points are now discussed in the revised manuscript.

3）Finally, what happens to the LH activity with diazepam? (it seems likely that diazepam might in part induce sleep by, for example, inhibiting the LH orexin cells?)

We have added new experiment with DZP treatment and LH population imaging, targeting LH-Vgat and LH-Hcrt/OX neurons. The new results have been added as Supplementary Fig. 11, described in the revised result section and discussed.

In brief, we found that in LH-Vgat neurons, DZP administration at ZT12 produced a modest, non-significant increase in the proportion of NREM-active neurons, which was less pronounced than the effect observed in POA-Vgat neurons, while LH-Hcrt/OX neurons showed minimal responses at both time points. These results indicate a region-specific effect of DZP in the POA rather than a general effect across hypothalamic populations.

Minor point:
Line 48: "Inhibitory neurons in the preoptic area are predominantly active during NREM, and to a lesser extent REM". Ref. 24 (Miracca et al) is cited as showing this. In fact, data in that paper show that all types of neurons in the lateral preoptic

hypothalamus, including vgat ones, are more active in REM (e.g. see the vgat neural activity in Fig. 1F of that paper). This is in agreement with Yan et al’s current findings.

We agree with the findings from Miracca et al. Our present results confirmed this and we have corrected this in the revised introduction stating that inhibitory cells in the POA are active during both NREM and REM sleep.

“Relatively rapid time scale of shifts in state-selective activity patterns suggested that these changes are not sole input-driven … and may include specific gene expression, protein translation…”. Gene expression and translation are relatively slow and not relatively rapid? Maybe qualify, reword, make more precise what is meant by "rapid"?

It's true the expression here is imprecise. Our intention was to indicate the observed shifts occur on a timescale shorter than large-scale structural neuronal circuit reorganization. We have clarified this point in the discussion.

Happy to sign as: William Wisden

Reviewer #2 (Remarks to the Author):

Yan et al. investigated the longitudinal stability of single-cell sleep-state selectivity in genetically defined neuronal populations in the lateral hypothalamus (LH). Using single-cell calcium imaging in freely moving sleeping mice, the authors observed that LH sub-populations show different sleep-state activities and that the selectivity of single neurons shifts over time. However, at the population level, the distribution of state-active neurons remains stable and is robust despite acute disruptions. These findings are consistent in both the LH and POA. Additionally, diazepam was found to increase the activity and NREM sleep selectivity of POA-Vgat neurons.

We thank the reviewer for the supportive comments and thoughtful suggestions. We have addressed those as described below.

Major comments:

1. The first major concern regarding the definition of state selectivity arises from the potential conflation of neuronal activity behavioral with intensity within wake.
The method of defining neuronal state specificity based on the highest event rate within a recording session is potentially confounded by the heterogeneity of behavioral substates. For instance, a session with a high proportion of active wakefulness (e.g., exploration, grooming) is likely to have a higher overall wake event frequency in many neurons compared to a session dominated by quiet wakefulness.
Consequently, a neuron that is genuinely selective for "high-arousal" behaviors might

be misclassified as "wake-specific" in one session and not in another, purely due to the difference in the animal's behavioral composition during those recordings. This ambiguity directly impacts the interpretation of both "stable" and "switching" neurons reported in the study.

To rule out this alternative explanation, it is essential to perform control analyses. For example, the authors could:

1. Subcategorize wake epochs into active and quiet wake to check if the putative "wake-active" neurons are specifically tuned to one substate.
2. Examine the correlation between behavioral intensity metrics (e.g., velocity during wake) and neuronal event rates within each state and across sessions.

Ensuring that the observed shifts in selectivity are not merely artifacts of varying behavioral dynamics would greatly strengthen the central conclusion about the intrinsic dynamics of hypothalamic populations.

We appreciate this thoughtful comment. Considering the multifunctional roles of LH subpopulations beyond sleep regulation, we agree that distinguishing wake substates is important to exclude potential confounds related to behavioral intensity, thus, consolidate our fingdings. Since velocity or behavioral video was not recorded in parallel with EEG/EMG recordings, we used a 3-stage vigilance scoring combined with EMG amplitude as a proxy for active behavior such as locomotion, an approach commonly used to distinguish active from quiet wake states *(Fraigne JJ, Sleep. 2023)*.

The methods and corresponding results have now been included in the revised manuscript in **Supplementary Fig. 8**. In brief, although excluding active wake reduced overall wake-related activity and redistributed a subset of wake-classified neurons, it did not significantly alter the distribution or the average number of state-selective shifts per neuron compared with the original three-state classification. Importantly, this new analysis did not change the conclusions of our study.

2. Assigning a single state label to each neuron may not fully capture the complexity of neuronal activity across sleep-wake cycles.

The study defines state specificity primarily based on which sleep-wake state has the highest frequency of calcium events. However, a significant question arises: for neurons that exhibit comparable levels of activity in two behavioral states (e.g., wake and REM), is it biologically meaningful or methodologically sound to assign a single, discrete state label based solely on a marginal difference in event rate?

Furthermore, the transition of a neuron's state preference is likely a gradational process rather than an instantaneous switch. The current analytical approach, which assigns a fixed selectivity to each neuron within a recording session, may not adequately capture this dynamism. It would be crucial to consider whether neurons active in multiple states could represent a population that is in the process of transitioning their functional specificity. A more granular analysis—like the RGB map in Extended Data Fig.4 could significantly strengthen the interpretation of the data and provide deeper insights into the dynamic nature of neurons, as posited by the authors' conclusion.

We agreed and we repeated our classification analysis by adding 4 new categories to take into account neurons that exhibit comparable levels of activity instead of assigning neurons to a state depending on its maximum: Wake & REM, NREM & REM,

Wake & NREM, Unspecific.

This new analysis (Supplementary Fig. 5) shows how most of the cells that were clustered as REM sleep active cells, because of their maximal activity in the REM sleep, fit in the new Wake & REM category.

We further added a quantification of each neuron's state preference on a triangular space, where each extreme represents an ideal neuron that is perfectly selective for one of the 3 states, i.e., Wake, REM, NREM. A dynamic, visual example of how each neuron's position evolves in this space at each recording time point is provided in the supplementary Movie S1 and S2.

In addition, we included 3 summary plots (Supplementary fig. 6) that show:
- the average distance of each cell from each of the 3 extremes of the space
- the average state selectivity of each cell (from 0 to 1)
- the average selectivity *variation* between consecutive recording sessions of each cell.

For rigour and easier interpretability, we included a dashed line that represents what the distance and variability would be if neurons had random positions (i.e., chance level).

3. To more compellingly demonstrate the activity patterns of neurons, it would be helpful to include representative raw calcium trace examples in each brain state. For example, the finding that a subset of orexin neurons exhibits activity during NREM and REM sleep is a significant departure from the established model of their exclusive wake-promoting function. Raw trace data would provide the most direct and convincing evidence for this claim:
1. Raw trace of orexin neurons have maximal Ca^{2+} event frequency during each of the three states (Wake, NREM, REM) within a recording session.
2. Examples of a single orexin neuron undergoing a switch in its state preference (e.g., from wake-active to REM-active) across different sessions.

We agree with the reviewer that having some representative raw calcium trace would be very straightforward to observe the single neuron activity profile and selectivity drift across sessions. Thus, we now have added a Supplementary Fig. 4, where we show some representative traces of neuron activities (calcium transients) within a single recording session or across separate recording sessions in all the populations studied.

4. The conclusion that sleep deprivation has minimal impact on population-level state selectivity requires further validation with a more robust paradigm. The authors conclude that acute sleep deprivation (SD) does not significantly alter the overall distribution of state-specific neural populations, a finding that is intriguing. However, as shown in Extended Data Fig.5, the 4-hour SD protocol employed—while sufficient to induce homeostatic sleep pressure—resulted in relatively moderate changes in subsequent sleep architecture and slow-wave activity (SWA). It is therefore plausible that a more prolonged or intense sleep deprivation challenge could overwhelm the proposed stability mechanism and reveal significant reorganization within the hypothalamic and preoptic circuits. To robustly support the claim of resilience, it would be highly informative to investigate whether the stability of neuronal population distributions holds under conditions of

stronger sleep pressure, for example, following 6 or 12 hours of sleep deprivation. Demonstrating that the system remains stable even under a more severe challenge would greatly strengthen the main conclusion.

The 4-hour SD protocol used in our study already produced an expected increase in NREM sleep duration and SWA during sleep recovery. Notably, each recording session lasted approximately 30 minutes during this sleep recovery. Although a 6h sleep deprivation may have further increased those, our time window analysis indicates that the protocol was sufficient to engage physiological sleep homeostasis. Our rationale was to not use a 6-h SD protocol since it requires to touch the animals and eventually activate the physiological stress system and therefore reduce the specificity of our results. In our hands, we doubt we could reliably total sleep deprived a mouse for 12h.

Yet we performed additional analysis. We quantified single-cell event frequency changes across LH subpopulations. We observed heterogeneous, state-dependent modulation at the single-neuron level, with distinct response profiles across neuronal subtypes. When analyzed at the animal level, a consistent reduction in REM-associated activity was specifically observed in LH-Vgat mice, indicating differential sensitivity to SD across the LH subpopulations. In general, even under a protocol that effectively induced homeostatic sleep pressure and measurable neuronal modulation, population-level neuronal activity and neuron clusters remained largely preserved.

Longer SD paradigms may induce stronger sleep pressure during the sleep rebound, even aside from animal welfare considerations, they would also introduce additional confounding factors, such as stress responses and circadian disruption, making interpretation of state-selective reorganization more complex.

5. The characterization of the minority of neurons that appear state-stable across recordings requires deeper investigation to distinguish biological significance from statistical chance.
The study reports a subset of neurons that maintained their state-specificity across long-term imaging. A critical question arises: do these "stable" neurons possess distinct electrophysiological or molecular properties, or could their apparent stability arise simply from stochastic probability?
To robustly address this, it would be highly informative to perform a more rigorous statistical analysis. Specifically, constructing a transition probability matrix for state selectivity at the single-neuron level across sessions would be valuable.
Furthermore, comparing the observed prevalence of stable neurons against a null statistical model (e.g., one based on the overall probabilities of state assignment) could determine whether their persistence exceeds what would be expected by chance alone. Such an analysis would significantly strengthen the interpretation of these neurons, either by highlighting a biologically unique subpopulation or by confirming that the observed stability is consistent with random variation.

We thank the reviewer for this very good suggestion. As suggested, we added a Supplementary Figure 7 that explicitly quantifies the probability of transition between each state plus standard error across longitudinal recordings. We included two comparisons: with neuron state assignments preserved, but with each single assignment permuted in time (this preserves the total number of neurons assigned to

each state), and another simple case where each neuron's assignment is purely random.

The fact that the average probabilities of transition are comparable to the permuted case highlights how the system is, at the population level, in a stable state, consistently with the result shown in Fig.3, despite each cell's high variability.

Aside from statistical analysis, we believe that answering such interesting questions would require other techniques such as molecular profiling of cells sub-populations, or, inputs/outputs circuit activity tagging, which are both challenging and outside the scope of this study.

6. The finding that diazepam (DZP) recruits more NREM sleep-active neurons in the POA should be extended to determine its generality. The authors present a compelling pharmacological result demonstrating that diazepam increases the proportion of NREM sleep-specific neurons within the POA Vgat population. However, to establish whether this is a general mechanism of DZP action or a phenomenon specific to the POA, it is crucial to replicate this experiment in the lateral hypothalamus (LH). Given that the study extensively characterizes the dynamic nature of various genetically defined cell types in the LH (e.g., hypocretin/orexin-, MCH-, Vglut2-, and Vgat-expressing neurons), testing the effect of DZP on these populations would be highly informative.

We have now extended the DZP treatment experiment to two LH populations, specifically in LH-Vgat and LH-Hcrt/OX neurons, and the results have been added as Supplementary Fig. 11.

In LH-Vgat neurons, DZP administration at ZT12 produced a modest, non-significant increase in the proportion of NREM-active neurons, which was less pronounced than the effect observed in POA-Vgat neurons, while LH-Hcrt/OX neurons showed minimal responses at both time points. These results indicate a region-specific effect of DZP in the POA rather than a general effect across hypothalamic populations.

These results are now described and discussed in the revised manuscript.

Minor comments:

1. Regarding the calcium signal extraction methodology: The authors mention that ROIs were manually defined in Fiji/ImageJ. While this is a valid approach, numerous established, automated, and publicly available pipelines (e.g., CaImAn, CNMF-E) have been developed to perform cell detection and signal extraction in an objective and high-throughput manner. Could the authors please clarify the rationale for choosing manual ROI definition over these automated methods?

In this study ROIs were manually defined in Fiji to ensure reliable identification and longitudinal tracking of the same neurons across recording sessions. Because our study required consistent monitoring of individual cells over multiple days within a fixed field of view, manual ROI selection combined with careful cross-session registration allowed precise matching of neuronal identities, particularly in cases of variable signal intensity or closely adjacent cells where automated methods may merge or split ROIs.

We agree that automated pipelines such as PCA-ICA or CNMF-E provide robust and

high-throughput approaches. However, implementing these methods in a longitudinal framework may require concatenating all sessions to generate a consistent ROI set across days. In our dataset, minor field-of-view shifts needed careful spatial correction, and concatenating long recordings substantially increased data size and computational complexity. Given the moderate number of neurons per animal (less than 100 neurons) and the need for accurate longitudinal tracking, manual ROI provided a reliable and controlled approach for this study.

Of note, our previous study used CNMF-E (Oesch et al 2020) and those results were confirmed here, however, such approaches required drastic downsampling (due to the very high number of frames) which decrease temporal resolution and data quality.

2. Regarding the calcium signal processing methods: The manuscript mentions that an exponential curve was fit to the data for bleaching correction. Please could clarify:(1) The specific mathematical function used for this exponential fit (e.g., a single- or multi-exponential function).(2) Whether this fit was performed on the entire recording session or specifically on periods of NREM sleep data.

We have specified in the Methods section that we used a single exponential function fit for the entire recording session.

3. Regarding the analysis of calcium activity: The study primarily utilizes the frequency of calcium events as the key metric for neuronal activity. While event frequency is informative, it does not capture the potential variability in the amplitude or duration of calcium transients, which may also carry biological significance. Have the authors considered complementary measures, such as the area under the curve (AUC or integral) of the calcium transients? Analyzing both frequency and AUC would strengthen the analysis by ensuring that the conclusions are robust to the choice of activity metric.

In addition to calcium event frequency, we performed complementary analyses using the integral of the whole calcium traces as an alternative measure of neuronal activity. Integral-based results are partially presented in the sleep deprivation analysis. We further clustered neurons based on the integral metric and tracked their cluster transitions across sessions, like what we performed in Fig. 2. This analysis revealed a similar drift in single-neuron state preference over time.

An integral-based analysis is more sensitive to global activity fluctuations associated with changes in macroscopic brain states. In particular, due to the fragmented nature of sleep in mice, we find that an event-rate analysis can produce more robust results, as correcting for these state-specific fluctuations would per se introduce some bias.

Furthermore, our decision to use events rates instead of the integrals of the events, was due to the fact that defining the event boundaries, in particular the end of an event, is much more challenging than simply counting the events and their peaks, making an events integral measure less robust. For this reason, we chose event frequency as the primary metric for state-selective analyses, while still reporting an integrals-based analysis as a sanity check, showing that the main conclusions are robust across both

measures.

4. Regarding Figure 3: It would be helpful to clarify why the betweenness centrality analysis was not included for LH-MCH neurons. Was this omission due to an insufficient number of neurons for a robust network analysis?

The betweenness centrality analysis was not performed for LH-MCH neurons due to the comparatively low expression of GCaMP in MCH-Cre mice, which resulted in a limited number of recorded neurons. The number of neurons was insufficient to support a robust and reliable network-level analysis.This has now been clarified in the revised manuscript.

5. An analysis of single-neuron state transition probabilities would be valuable. Quantifying these transitions after sleep deprivation and DZP administration would provide more direct evidence.

We have now included a state transition probability analysis across longitudinal recording sessions (Supplementary Fig. 7), which explicitly quantifies the probability of transitioning between state-selective categories across the 7 recording days.

For the sleep deprivation and acute DZP experiments, recordings consisted of only two sessions (before–after for SD, and saline–DZP for acute treatment). We applied the same transition probability analysis to these datasets. However, given the two-session design, the resulting transition matrices are mathematically equivalent to the proportion of neurons switching between clusters, which is already reported in the Results section. Thus, this analysis did not provide additional information beyond the percentage of neurons in each state-selective category.

For the POA-Vgat multi-day DZP experiment (3 consecutive days of saline and DZP), we additionally performed transition probability analysis across sessions. No significant differences in transition structure were detected between saline and DZP conditions, and both were comparable to permuted controls, indicating that DZP did not impose a structured transition bias beyond changes in overall state occupancy. As this analysis did not alter the interpretation of the results, and to avoid redundancy, we did not include these data in the main manuscript but can provide them upon request.

6. Please indicate the statistical significance of the state-specific changes induced by DZP in Figure 5B. Adding p-values or significance notations to the figure would be better.

Statistical results have been added in the figure; the raw data and statistical information are both included in the Source Data file.

Reviewer #3 (Remarks to the Author):

Yan et al. used a miniature microendoscope with single-cell resolution to record various types of neural activity in the brains of mice that were freely showing sleep/wakefulness over a long period of time, demonstrating that these activity patterns can change over time. Previous studies had suggested that activity patterns remained largely unchanged across sleep/wake states. So the concept of this manuscript is interesting; however, it is unfortunate that there is insufficient evidence to support it. The main issue is that there is no clear evidence on whether the authors reliably and continuously recorded the same neurons or not. In this study, this point should be rigorously addressed and presented with different aspects, yet the current manuscript does not provide sufficient validation of this aspect. This reviewer suggests the following points to verify the importance and credibility of the contents of this manuscript.

We thank the reviewer for the supportive comments and suggestions, which we have addressed below.

Major comments

1) There are various ways to prove that the same neural activity is being recorded.

・Use another protein that emits constant fluorescence as a position marker (Sato et al., 2020, Cell Rep)

・Verify the technique using a system where it is already known that the same neurons remain active over time

・Use Cal-Light, scFLARE2, CaMPARI, KikGR, etc., to confirm post-hoc using optotags or fixed brains

We thank the reviewer for these suggestions and for proposing multiple technical approaches to validate longitudinal cell tracking. In our study, we used a stable and intrinsic anatomical landmark—blood vessel patterns—to identify and align neurons across recording days. After full post-surgical recovery (≥4 weeks) in adult animals, vascular morphology remains unchanged (Cudmore et al. *J Cereb Blood Flow Metab.*, 2017) and provides high-contrast structural features within the field of view. We therefore used blood vessel patterns to register imaging fields and confirm consistent neuron identity across sessions (see new Supplementary Fig. 3, showing representative ROIs across several days within the same FOV containing identifiable BV landmarks). Importantly, vascular morphology assists not only in XY-plane but also in confirming stability along the Z-axis, as vessel contrast and relative depth help verify that lens position and focal plane did not shift across sessions. This approach has now been described in the Methods section of the revised manuscript.

2) The NREM activity of orexin in Figure 2A is very low. The activity of orexin neurons during NREM sleep has been shown to exhibit synchronized activity in data from the Luis deLecea lab (Li et al., Science 2022) and the Akihiro Yamanaka lab (Ito et al., PNAS 2023), so the NREM activity of 2% seems unusual. This is probably because the authors define the maximum value of activity as “state-active,” so

NREM-active neurons are almost completely absorbed by wake-active neurons, which may lead to this situation. In other words, even if wake-REM active and wake-NREM active actually exist, an approach that defines and discusses only maximum activity may be a method that hinders a direct understanding of the essence of the phenomenon. When the criteria in the above definition are relaxed or changed, it seems necessary to at least confirm how these activity patterns will change.

We thank the reviewer for this insightful comment. We may not agree on the term ‘synchronized activity’ here but we do agree on the activity of hcrt/ox neurons outside wakefulness. In fact, we do observe activity of hcrt/ox neurons during NREM sleep, as shown in Fig. 1 and new Supplementary Fig. 4. We agree that when using a maximum event-rate criterion for state assignment, neurons active during NREM may be classified according to their higher activity during REM sleep or wakefulness.

However, our conclusions do not rely solely on this maximum-based classification. To address this concern, we also applied two new complementary analytical approaches, which are now clarified in the revised manuscript:

1. **Threshold-based classification (new Supplementary Fig. 5a)**
   Neurons were categorized as single-state– or multi-state–selective based on relative activity levels across vigilance states, rather than strict maximum activity. This approach captures neurons with substantial activity in multiple states, such as wake–REM, NREM-REM co-active populations.

2. **Category-independent analysis (new Supplementary Fig. 5b,c; new Supplementary Fig. 6 and Movie S1)**
   We analyzed Ca^{2+} event rates across all vigilance states without assigning fixed state labels to individual neurons. This avoids potential bias introduced by categorical definitions and allows direct visualization of state-dependent modulation.

Both analyses consistently demonstrate state-dependent reorganization of neuron activity, while accounting for the fact that most neurons are active across multiple vigilance states.

3) The selectivity of gene expression using the short orexin/hypocretin promoter with the virus vector is relatively lower compared to the gene-modified mouse line. It is necessary to confirm whether the expression pattern changes across time using histochemistry. It is better to use a gene-modified mouse line, such as the orexin-Cre mouse, instead of a virus vector when the authors would like to claim this type of phenomenon.

We understand the comment but we respectfully disagree with it. In most experiments, we used Cre-driver mouse lines to ensure cell-type specificity. However, for the Hcrt/Ox experiments, Hcrt-Cre mice were not available in our laboratory, so we used the AAV-hORX-GCaMP6 virus to target orexin neurons. This strategy has been validated by many groups worldwide including ours (Adamantidis et al 2007, Carter et al 2009, 2011; Burdakov’s group, etc.), who reported 97.4 ± 1.0% specificity and 65.8

± 3.7% penetrance in hypocretin/orexin neurons (Karnani et al., *Prog Neurobiol*. 2020). This promoter has been widely used to image orexin neuron activity.

Historically, there have been few orexin-Cre driver with variable penetrance. By comparison, another study using Hcrt-Cre mice combined with a DIO-GCaMP6 virus reported comparely lower specificity (52.76 ± 26.29%; Tyree et al.,*Commun Biol,* 2023), suggesting that specificity can vary depending on the approach (virus vs cre driver line) and experimental conditions (possibly virus serotypes, promoter, titer, etc.). In our study, we performed post hoc immunohistochemistry with an orexin-A antibody and found that approximately 86.78 ± 1.83% of GCaMP-labeled neurons were orexin-A positive, as expected with other studies and shown in the figure below.

Overall, although labeling specificity can vary across methods, our validation indicates that the specificity in our experiments is comparable to or higher than reported averages and does not affect the reliability of our conclusions.

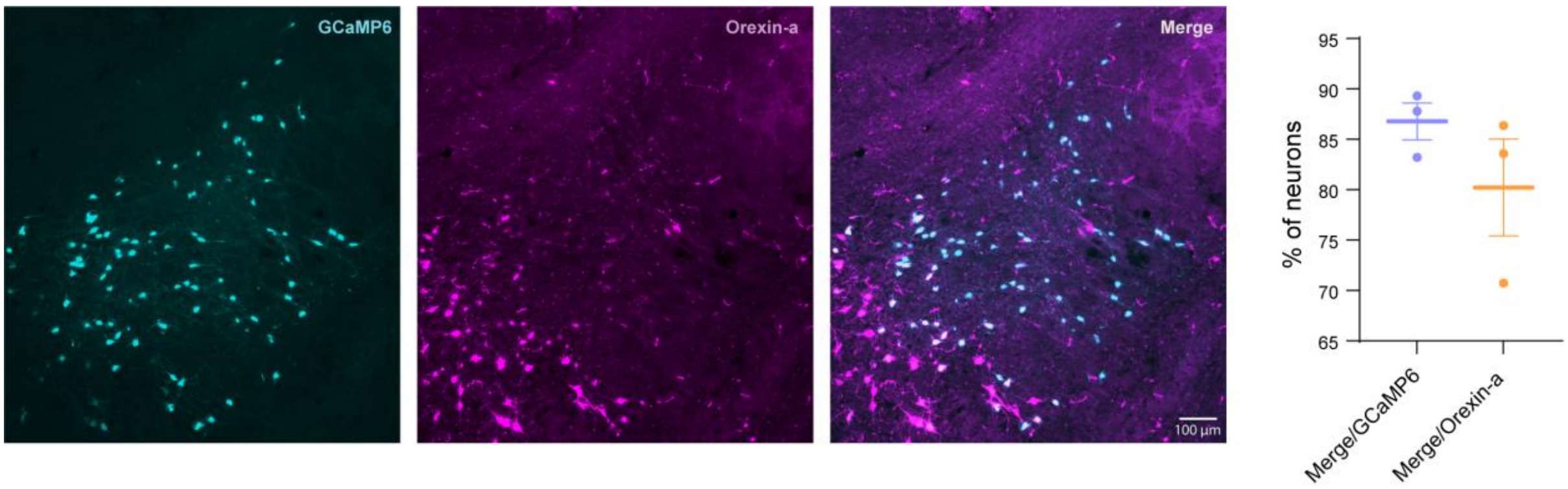

4) It is also interesting to confirm whether neural activity patterns change similarly during activities other than sleep/wakefulness, such as eating and drinking. This will clarify whether the changes are limited to sleep/wakefulness or whether other functions also change simultaneously. If changes are observed in other activities as well, the significance of these changes will be further heightened.

We agree that examining whether similar neuronal activity drift occurs during other behaviors, such as feeding or drinking, would be very interesting. However, we had to limit our ambitions and focus on sleep/wake states. Addressing activity drift across other innate behaviors would require dedicated experimental paradigms beyond the scope of the current work. Notably, similar forms of representational drift have been reported in auditory and visual tasks, suggesting that such dynamics may extend beyond sleep regulation. We have clarified this point in the Discussion and highlighted it as a direction for future investigation.

Minor comments

1) Figure 1H, the caption indicates Vgat-Cre, this should be Vglut2-Cre.

2) The color of the legend does not match the color in Figure 2 (wake and inactive).

3) Figure 3, it is confusing to change the color of the unspecific between A-D and E-H.

4) The second and third neurons from the top of the recorded MCH neurons in Figure 1 are completely inactive. I think they might be detected as neurons in another session. I would like to see representative data that spans multiple days, rather than just data from this one session. I think that would give us a clearer picture of the actual activity pattern.

5) In Materials and methods, Vglut2-ires-Cre (Strain #:028863), : , typo.
6) In Materials and methods, 0.8 µm.s-1, and 0.1 mg.kg-1, it is better to use "/" which is used in DZP (5 mg/kg).
7) Uniform the writing of OX/Hcrt, VGlut2 neurons, and VGAT neurons throughout the manuscript.

We thank the reviewer for pointing out these issues. All typographical and formatting errors have been corrected in the revised submission files. In response to comment 4, we have included Supplementary Fig. 4 showing representative raw calcium traces within and across recording sessions for each neuronal type included in this study.

REVIEWER COMMENTS

Reviewer #1 (Remarks to the Author):

The authors have revised the manuscript convincingly. It will be of broad interest to those dissecting sleep–wake circuitry. The key result, that individual hypothalamic neurons show marked, state dependent changes in activity while the overall sleep–wake ensemble remains stable, is striking and unexpected. It points to a finely tuned balance between genetically anchored circuit architecture and functional flexibility in the neurons governing sleep and wakefulness, perhaps allowing the brain to preserve reliable sleep yet adapt to changing internal and external demands.

Happy to sign as William Wisden

Reviewer #2 (Remarks to the Author):

The authors have adequately addressed all my concerns in the revised manuscript. I am happy to recommend acceptance.

Reviewer #3 (Remarks to the Author):

This is a revised manuscript by Yan et al. The authors have responded carefully to the previous reviews, and the revised manuscript has been substantially improved. The newly added analyses, including wake substate analysis, threshold-based and category-independent state selectivity analyses, state transition probability analysis, and the diazepam experiment in the LH population, help address several concerns raised by the reviewers and make it clearer that the conclusions do not rely solely on maximal activity assignment.

However, this reviewer's main concern has only been partially resolved. The central claim of this study is that the sleep-wake state selectivity of individual neurons drifts over time based on longitudinal tracking of the same cells. This conclusion requires particularly strong evidence that identical cells were reliably registered across days.

In the revised manuscript, the authors now explain that the miniature microscope remained attached throughout the recording period, focus was fixed, the same field of view was maintained, vascular landmarks were used for alignment, and manual ROIs were defined while viewing all recording sessions. These details are helpful and improve the manuscript. Still, they are not sufficient to quantitatively establish that each ROI corresponds to the same cell across days. Vascular landmarks support field-level stability, but they do not fully exclude mismatches between nearby cells, subtle focal plane shifts, day-to-day signal fluctuations, or errors introduced by the custom ROI extraction and registration pipeline. Therefore, it remains difficult to rule out the possibility that at least part of the reported representational drift reflects technical mismatch rather than biology.

1) To strengthen this key point, the revised manuscript still requires more direct

validation of longitudinal cell registration. In particular, visual examples and field-level alignment using vascular landmarks are helpful, but they do not yet provide a quantitative estimate of registration accuracy or false-match rate across days. At minimum, the authors should provide a more quantitative validation, for example using ROI footprint overlap, centroid displacement, matching confidence, or shuffle/mismatch-based false-match estimates. If feasible, an additional supplementary cross-check using an established longitudinal registration pipeline on a representative subset of the data would further strengthen confidence in the conclusions. A negative-control dataset or population expected to show relatively stable assignment would also be valuable. At present, it remains difficult to determine to what extent the observed remapping reflects a genuine biological phenomenon versus technical mismatch introduced during cross-session registration.

We understand and agree with the reviewer that convincing validation of longitudinal neuronal alignment is essential for interpreting the results. We thank the reviewer for suggesting several ways to further evaluate this point. We would first like to clarify that our analysis did not involve independently segmenting ROIs in each session followed by cross-day cell matching. Instead, for each animal, a single common ROI set was defined after inspecting all recording sessions, and this same ROI set was then used to extract dF/F0 signals from every session. This was indicated in the methods section. Therefore, direct measures such as ROI footprint overlap, centroid displacement, or matching confidence between independently detected ROIs are not directly applicable to our analysis. The more relevant question is whether the field of view remained sufficiently stable for the common ROI masks to sample the same fluorescence sources across days.

To address this point, we added new quantitative validation analyses in **Supplementary Fig. 3**. First, we calculated a shuffle-based false-match probability using local image similarity within each ROI footprint across sessions. For each ROI, the true cross-day image correlation was compared with correlations from shuffled ROI pairings, allowing us to estimate the probability that an apparent longitudinal match could occur by chance. As expected, the false-match probability increased with the interval between recording days; however, the mean of the median false-match probability across days remained low in all groups: 0.130 for LH-Vgat, 0.053 for LH-Vglut2 and 0.087 for LH-Hcrt/OX neurons. Second, we quantified consecutive-day FOV displacement from maximum projection images, with mean total shifts of 1.98, 2.69 and 3.72 pixels for LH-Vgat, LH-Vglut2 and LH-Hcrt/OX datasets, respectively.

In addition, we performed an independent cross-validation of neuronal and FOV stability using CaliAli (*Vergara et al., 2025*), a published pipeline for multi-session one-photon imaging alignment. Using the CaliAli alignment-evaluation functions, we quantified neuronal spatial correlations from CaliAli-defined neuronal components and blood-vessel similarity scores across the 7-day recordings. Before CaliAli alignment, neuronal spatial correlations across days mostly ranged between 0.6 and 0.8, indicating substantial preservation of neuronal spatial patterns across longitudinal recordings. Blood-vessel similarity scores in each mouse line were significantly higher than the between-animal negative control and were not significantly different from the

intra-session positive control. Estimated non-rigid misalignment was all below 30% of neuronal size in all groups.

Together, these analyses indicate a stable neuronal and FOV registration across days and reduce the likelihood that the observed drift in single-cell sleep-state selectivity reflects technical mismatch during longitudinal imaging.

2) This reviewer also suggests softening the interpretation of the diazepam data. The revised text states that diazepam increased NREM-active neurons in POA-Vgat at ZT12, whereas it "did not induce a comparable effect" in LH. However, the rebuttal notes at least a similar directional, though non-significant, trend in the LH-Vgat data. Without a direct statistical comparison between POA and LH, it would be more appropriate to state that the effect was more clearly observed in POA-Vgat, rather than implying that the effect is absent in LH or specific to POA.

We thank the reviewer for this suggestion. We have adapted the wording accordingly as follows "*In contrast to POA-Vgat neurons, LH-Vgat neurons showed only a directional but non-significant increase in NREM-active neurons and NREM-associated Ca^{2+} activity at ZT12.*" and "...., *with non-significant trend also observed in LH-Vgat neurons.*".

Overall, I find the study interesting and agree that the manuscript has improved considerably. Nevertheless, because the central conclusion depends critically on longitudinal cell registration, additional validation—or at minimum, more quantitative support for registration accuracy—remains necessary.
