# Supplementary material for: Temporal drift of sleep-wake representations in hypothalamic neuronal ensembles
Source: Nat Commun. 2026 Jul 28;17:9137. doi: 10.1038/s41467-026-75792-1 (PMC13519010; doi:10.1038/s41467-026-75792-1)
Supplement: Supplementary file 3 [file 41467_2026_75792_MOESM3_ESM.pdf]

File name: **Supplementary Movie 1**

Description:
Representation of LH-Vgat neuronal activity dynamics across vigilance states over 21 recording sessions. Each point represents a neuron plotted in a triangular coordinate system, with each vertex corresponding to one vigilance state: Wake, NREM, or REM sleep (n = 509 neurons). The position of each neuron reflects the normalized frequency of calcium events in each state. Five LH-Vgat neurons are randomly selected and color-coded to illustrate the evolution of their state-dependent activity profiles across sessions.

File name: **Supplementary Movie 2**

Description:
Representation of LH-Vgat neuronal selectivity dynamics across vigilance states over 21 recording sessions. Each point represents a neuron plotted in the same triangular coordinate system as in Supplementary Movie 1 (n = 509 neurons). Neuronal positions reflect state selectivity, calculated as the Euclidean distance between a neuron's normalized activity vector and each state axis in a 3D Wake–NREM–REM space. In this plot, proximity to a vertex reflects stronger selectivity for that state. Five LH-Vgat neurons are randomly selected and color-coded to illustrate the evolution of their selectivity profiles across sessions.
