# Supplementary material for: Temporal drift of sleep-wake representations in hypothalamic neuronal ensembles
Source: Nat Commun. 2026 Jul 28;17:9137. doi: 10.1038/s41467-026-75792-1 (PMC13519010; doi:10.1038/s41467-026-75792-1)
Supplement: Supplementary file 4 [file 41467_2026_75792_MOESM6_ESM.pdf]

nature portfolio

Corresponding author(s): Antoine Adamantidis

Last updated by author(s): Mar 1, 2026

Reporting Summary

Nature Portfolio wishes to improve the reproducibility of the work that we publish. This form provides structure for consistency and transparency in reporting. For further information on Nature Portfolio policies, see our Editorial Policies and the Editorial Policy Checklist.

Statistics

For all statistical analyses, confirm that the following items are present in the figure legend, table legend, main text, or Methods section.

n/a	Confirmed	
☐	☒	The exact sample size (*n*) for each experimental group/condition, given as a discrete number and unit of measurement
☐	☒	A statement on whether measurements were taken from distinct samples or whether the same sample was measured repeatedly
☐	☒	The statistical test(s) used AND whether they are one- or two-sided<br>*Only common tests should be described solely by name; describe more complex techniques in the Methods section.*
☐	☒	A description of all covariates tested
☐	☒	A description of any assumptions or corrections, such as tests of normality and adjustment for multiple comparisons
☐	☒	A full description of the statistical parameters including central tendency (e.g. means) or other basic estimates (e.g. regression coefficient) AND variation (e.g. standard deviation) or associated estimates of uncertainty (e.g. confidence intervals)
☐	☒	For null hypothesis testing, the test statistic (e.g. *F*, *t*, *r*) with confidence intervals, effect sizes, degrees of freedom and *P* value noted<br>*Give P values as exact values whenever suitable.*
☒	☐	For Bayesian analysis, information on the choice of priors and Markov chain Monte Carlo settings
☒	☐	For hierarchical and complex designs, identification of the appropriate level for tests and full reporting of outcomes
☒	☐	Estimates of effect sizes (e.g. Cohen's *d*, Pearson's *r*), indicating how they were calculated

Our web collection on statistics for biologists contains articles on many of the points above.

Software and code

Policy information about availability of computer code

Data collection	In this study, we primarily used commercial software for data collection. Calcium imaging data were acquired using Inscopix Data Acquisition Software (IDAS, version 1.4.0) with the nVista 3 miniature microscope. EEG and EMG signals were recorded using SleepScore (ViewPoint Life Sciences V1.9.1) in conjunction with an AM Systems Model 3500 amplifier, and digitized via a National Instruments USB X DAQ device.
Data analysis	Inscopix Data Processing Software (version 1.9.1): Used for motion correction and preprocessing of calcium imaging videos. Fiji (ImageJ, version 1.53t): Used for calcium imaging videos background subtraction, manual ROI selection, and fluorescence trace extraction across sessions. MATLAB (R2019b, MathWorks): Used for sleep stage scoring, calcium trace normalization, calcium event detection in each sleep stages and statistical analysis. MATLAB (R2025b, MathWorks) and CaliAli (V 1.4.8) used for calcium imaging field of view validation. SWA-MATLAB toolbox: Used to detect individual slow waves during NREM sleep. GraphPad Prism (version 10.0): Used for visualization and group-level statistical tests. SleepScore (ViewPoint Life Sciences, version 3.1): Used for sleep stage scoring. These tools are widely used in neuroscience for data preprocessing, event detection, and statistical analysis. Custom MATLAB code were developed in this study, relevant scripts and instructions are available here: https://github.com/ZENLabCode/CalciumImaging.

For manuscripts utilizing custom algorithms or software that are central to the research but not yet described in published literature, software must be made available to editors and reviewers. We strongly encourage code deposition in a community repository (e.g. GitHub). See the Nature Portfolio guidelines for submitting code & software for further information.

Data

Policy information about availability of data

All manuscripts must include a data availability statement. This statement should provide the following information, where applicable:

- Accession codes, unique identifiers, or web links for publicly available datasets
- A description of any restrictions on data availability
- For clinical datasets or third party data, please ensure that the statement adheres to our policy

Source data associate with figures are provided as a Source Data file with the paper. In addition, raw data files for calcium event detection are provided with Figshare DOI: https://doi.org/10.6084/m9.figshare.31441240.

Research involving human participants, their data, or biological material

Policy information about studies with human participants or human data. See also policy information about sex, gender (identity/presentation), and sexual orientation and race, ethnicity and racism.

Reporting on sex and gender	*Use the terms sex (biological attribute) and gender (shaped by social and cultural circumstances) carefully in order to avoid confusing both terms. Indicate if findings apply to only one sex or gender; describe whether sex and gender were considered in study design; whether sex and/or gender was determined based on self-reporting or assigned and methods used. Provide in the source data disaggregated sex and gender data, where this information has been collected, and if consent has been obtained for sharing of individual-level data; provide overall numbers in this Reporting Summary. Please state if this information has not been collected. Report sex- and gender-based analyses where performed, justify reasons for lack of sex- and gender-based analysis.*
Reporting on race, ethnicity, or other socially relevant groupings	*Please specify the socially constructed or socially relevant categorization variable(s) used in your manuscript and explain why they were used. Please note that such variables should not be used as proxies for other socially constructed/relevant variables (for example, race or ethnicity should not be used as a proxy for socioeconomic status). Provide clear definitions of the relevant terms used, how they were provided (by the participants/respondents, the researchers, or third parties), and the method(s) used to classify people into the different categories (e.g. self-report, census or administrative data, social media data, etc.) Please provide details about how you controlled for confounding variables in your analyses.*
Population characteristics	*Describe the covariate-relevant population characteristics of the human research participants (e.g. age, genotypic information, past and current diagnosis and treatment categories). If you filled out the behavioural & social sciences study design questions and have nothing to add here, write "See above."*
Recruitment	*Describe how participants were recruited. Outline any potential self-selection bias or other biases that may be present and how these are likely to impact results.*
Ethics oversight	*Identify the organization(s) that approved the study protocol.*

Note that full information on the approval of the study protocol must also be provided in the manuscript.

Field-specific reporting

Please select the one below that is the best fit for your research. If you are not sure, read the appropriate sections before making your selection.

☒ Life sciences ☐ Behavioural & social sciences ☐ Ecological, evolutionary & environmental sciences

For a reference copy of the document with all sections, see nature.com/documents/nr-reporting-summary-flat.pdf

Life sciences study design

All studies must disclose on these points even when the disclosure is negative.

Sample size	The study did not involve a pre-determined sample-size calculation. Instead, the sample size was selected to balance ethical considerations—minimizing the number of animals used—with the need to obtain statistically meaningful results. This sample size was based on prior published studies using in vivo calcium imaging and polysomnography in freely behaving mice and is consistent with experimental designs in similar longitudinal studies.
Data exclusions	The only excluded data involved animals with poor GRIN lens placement or insufficient virus expression, which led to unstable or low-quality calcium imaging signals. These issues were confirmed through anatomical verification. All other data from animals that completed the experimental protocol were included in the analysis.
Replication	The experiments were replicated using independent biological samples, with individual mice considered the experimental unit. Each mouse was recorded longitudinally across multiple sessions, these repeated recordings were used to assess within-animal longitudinal dynamics, whereas statistical comparisons were performed as described in the manuscript. For pharmacological experiments, control and drug conditions were tested in the same animals using a within-animal design. Individual mice were defined as the biological replicates and experimental units. No technical replicates were used.

Randomization	All animals underwent the same recording protocol to assess longitudinal neuronal activity across sleep-wake states. As the study design did not involve separate control or treatment groups for these recordings, randomization was not applicable. In the drug treatment experiments, a within-subject design was used where each mouse served as its own control, eliminating the need for group allocation.
Blinding	Blinding of investigators to group allocation during data collection and analysis was not feasible due to the longitudinal design of the imaging and pharmacological monitoring protocols. However, this limitation is mitigated by the fact that most analyses were performed using automated or standardized pipelines that did not require subjective interpretation. The outcomes assessed—such as calcium activity, sleep-state classification, and slow wave detection—were based on objective and quantifiable metrics, minimizing the potential for investigator bias. Given the automated nature of the analysis and the lack of subjective decision-making, blinding was not deemed necessary for this study.

Reporting for specific materials, systems and methods

We require information from authors about some types of materials, experimental systems and methods used in many studies. Here, indicate whether each material, system or method listed is relevant to your study. If you are not sure if a list item applies to your research, read the appropriate section before selecting a response.

Materials & experimental systems

n/a	Involved in the study
☐	☒ Antibodies
☒	☐ Eukaryotic cell lines
☒	☐ Palaeontology and archaeology
☐	☒ Animals and other organisms
☒	☐ Clinical data
☒	☐ Dual use research of concern
☒	☐ Plants

Methods

n/a	Involved in the study
☒	☐ ChIP-seq
☒	☐ Flow cytometry
☒	☐ MRI-based neuroimaging

Antibodies

Antibodies used	Primary: anti-pMCH (goat, 1:500, sc-14509, Santa Cruz Biotechnology) and anti-Orexin A (rabbit, 1:1000, H-003-30, Phoenix Pharmaceuticals); Secondary: AlexaFluor555-conjugated secondary antibody (1:1000 dilution, Invitrogen, A21432).
Validation	The primary antibodies used in the experiments are both commercially available, widely used in neuroscience research, and validated for immunohistochemical applications in rodent brain tissue. The anti-pMCH antibody shows specific labeling of pMCH-expressing neurons in the lateral hypothalamus, and the anti-Orexin A antibody selectively detects orexin A without cross-reactivity to orexin B, as documented by the manufacturers and in previously published studies.The secondary antibody, AlexaFluor555-conjugated (1:1000, Invitrogen, A21432), is a well-established fluorophore-labeled antibody with minimal cross-reactivity, validated by the manufacturer for immunofluorescence. Negative controls omitting the primary antibodies were used to confirm the specificity of staining.

Animals and other research organisms

Policy information about studies involving animals; ARRIVE guidelines recommended for reporting animal research, and Sex and Gender in Research

Laboratory animals	Adult C57BL/6JRj mice (Janvier Labs, France; 8–24 weeks; male and female) and transgenic Vgat-ires-Cre (JAX #016962; male and female), Vglut2-ires-Cre (JAX #028863; males), and MCH-ires-Cre (JAX #014099; males) mice were used. Transgenic founders were obtained from The Jackson Laboratory (USA) and subsequently bred in the departmental animal facility at the University of Bern.
Wild animals	N/A
Reporting on sex	Longitudinal imaging experiments were conducted using adult male mice only to reduce potential variability associated with the female estrous cycle in a longitudinal repeated-measures design. In the pharmacological treatment study, both male and female mice were included in approximately equal numbers. Data from males and females were pooled for the primary analyses, and sex was not included as an explicit factor in the statistical models. We did not observe an apparent sex-dependent pattern in the measured outcomes within the available sample; however, the study was not designed or powered to detect small sex-specific effects.
Field-collected samples	N/A
Ethics oversight	All procedures were conducted in accordance with protocols approved by the Veterinary Office of the Canton of Bern, Switzerland (license numbers BE 129/2020, BE122/2023 and BE57/2024). All experiments complied with the Swiss Animal Welfare Act and institutional ethical guidelines, and were designed to minimize animal suffering and reduce the number of animals used.

Note that full information on the approval of the study protocol must also be provided in the manuscript.

Plants

Seed stocks	*Report on the source of all seed stocks or other plant material used. If applicable, state the seed stock centre and catalogue number. If plant specimens were collected from the field, describe the collection location, date and sampling procedures.*
Novel plant genotypes	*Describe the methods by which all novel plant genotypes were produced. This includes those generated by transgenic approaches, gene editing, chemical/radiation-based mutagenesis and hybridization. For transgenic lines, describe the transformation method, the number of independent lines analyzed and the generation upon which experiments were performed. For gene-edited lines, describe the editor used, the endogenous sequence targeted for editing, the targeting guide RNA sequence (if applicable) and how the editor was applied.*
Authentication	*Describe any authentication procedures for each seed stock used or novel genotype generated. Describe any experiments used to assess the effect of a mutation and, where applicable, how potential secondary effects (e.g. second site T-DNA insertions, mosiacism, off-target gene editing) were examined.*
